# Unravelling venetoclax solvate behaviour: insights from crystal structures and computational surface analysis

**DOI:** 10.1107/S2052252525006785

**Published:** 2025-08-28

**Authors:** Eliška Zmeškalová, Tereza Havlůjová, Martin Babor, Marcela Tkadlecová, Jaroslav Havlíček, Tomáš Pekárek, Szymon Tomczak, Luděk Ridvan, Miroslav Šoóš

**Affiliations:** a Department of Chemical Engineering University of Chemistry and Technology in Prague Technická 3 16628Prague 6 Czechia; b Institute of Physics of the Czech Academy of Sciences Na Slovance 2 182 00Prague 8 Czechia; c Zentiva k.s., U Kabelovny 130, 10237Prague 10, Czechia; d Faculty of Science Jan Evangelista Purkyně University in Ústí nad Labem Pasteurova 3632/15 400 96Ústí nad Labem Czechia; e Department of Pharmaceutical Chemistry Poznan University of Medical Sciences 3 Rokietnicka 60-806Poznań Poland; Sun Yat-Sen University, China

**Keywords:** venetoclax, solvates, pharmaceuticals, crystal structures, desolvation, crystal engineering, Cambridge Structural Database, crystal morphology, intermolecular interactions, polymorphism

## Abstract

This study investigates venetoclax solvates and their nonsolvated forms through desolvation. Seven solvates were prepared and their structures solved from single-crystal X-ray diffraction data. Among these, two are cavity solvates while the remaining five are channel solvates, with three being isomorphous. De­solvation experiments led to two solvent-free crystalline polymorphs of venetoclax, forms A and B. Notably, the acetone solvate remained stable and did not convert to a nonsolvated form. Form B has a higher melting point and a faster intrinsic dissolution rate than form A. Advanced computational tools, including *Solvate Analyser* and *CSD-Particle*, provided insights into crystal surface properties and desolvation behaviour. Properties such as lattice energy, molecular interaction energy, attachment energy, surface rugosity and solvent arrangement within the crystal and on the surface were linked to solvate stability and subsequent transformation upon desolvation. A combination of FTIR, and ^13^C and ^15^N solid-state NMR spectroscopies showed that both short- and long-range molecular interactions and arrangements of venetoclax molecules in the desolvated forms closely resembled those in the parent solvates.

## Abstract 2

Seven venetoclax solvates were structurally characterized and studied for their desolvation behaviour, leading to the identification of two solvent-free polymorphs. Advanced crystallographic and computational analyses, including *Solvate Analyser*, *CSD-Particle*, FTIR and solid-state NMR, revealed key factors governing solvate stability and transformation. Notably, the acetone solvate exhibited exceptional stability and the desolvated forms retained the molecular arrangement of their parent solvates.

## 1. Introduction

**Figure 1:**
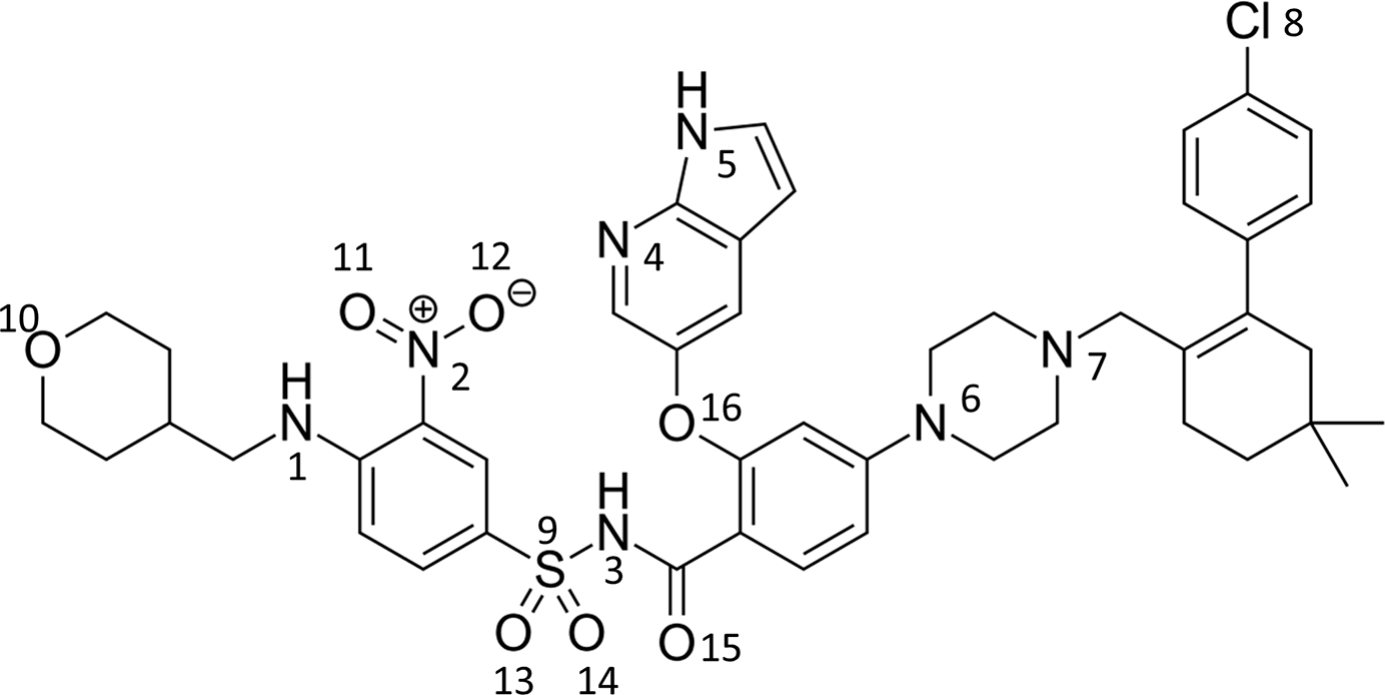
Structure of venetoclax with numbering that is used in the crystal structure discussion.

Venetoclax (Fig. 1 ▸) is a highly selective BCL-2 inhibitor first approved in the United States, and later in the EU, for monotherapy of chronic lymphocytic leukaemia in patients with 17p deletion or TP53 mutation (Salem *et al.*, 2017 ▸; Deeks, 2016 ▸) and later the approval extended to acute myeloid leukaemia and small lymphocytic lymphoma (European Medicines Agency, 2016 ▸). The mechanism of action focuses on triggering and restoring apoptosis in tumour cells by releasing pro-apoptotic proteins from the BCL2 family (Konopleva *et al.*, 2006 ▸). Venetoclax belongs to class IV of the Biopharmaceutical Classification System, indicating low solubility (<4 ng ml^−1^) and low permeability (Emami Riedmaier *et al.*, 2018 ▸). Absolute bioavailability estimation is only 5.4% of the administered dose (Alaarg *et al.*, 2022 ▸).

Drug solubility is crucial for absorption, bioavailability and therapeutic effectiveness. Poor water solubility can lead to poor bioavailability and pose challenges during drug development. Various methods, including chemical and physical modifications, are employed to enhance solubility (Mantri *et al.*, 2017 ▸). Chemical modifications involve salt formation, complexation and hydrophilic group introduction (Skořepová *et al.*, 2017 ▸; Skořepová *et al.*, 2016 ▸; Koehl *et al.*, 2022 ▸; Guillory, 2003 ▸), while physical changes include particle size reduction, carrier dispersion and crystal state modification (Mantri *et al.*, 2017 ▸; Zvoníček *et al.*, 2018 ▸; Skořepová *et al.*, 2014 ▸; Bernstein, 2011 ▸; Brittain, 2009 ▸). Crystal state modifications can include, for example, polymorph or solvate formation.

Polymorphs are distinct crystal structures, exhibiting different molecular arrangements and packing within their crystal lattice while keeping the same chemical composition (Brittain, 2009 ▸). The traditional way to explore polymorphism is by crystallization from various solvents, obtaining the possible forms, and investigating their potential for further use (Hilfiker, 2006 ▸). During crystallization from solution, solvent molecules can be incorporated into the crystal structure and form solvates. These can be classified into different categories based on the variability of the amount of entrapped solvent, such as stoichiometric/non-stoichiometric (Tieger, Kiss, Pokol, Finta, Dušek *et al.*, 2016 ▸; Tieger, Kiss, Pokol, Finta, Rohlíček *et al.*, 2016 ▸), or structure, for example, channel/cavity (Te, 2001 ▸; Price *et al.*, 2006 ▸; Sládková *et al.*, 2015 ▸; Zvoníček *et al.*, 2017 ▸) solvates. They exhibit distinct and, in some cases, beneficial physical and chemical properties compared with their non-solvated counterparts (Byrn, 2017 ▸). However, in general, solvates are not the preferred final forms for formulation, as the presence of organic solvents raises toxicity concerns. Moreover, they may be less stable due to potential desolvation during manufacturing (Caira *et al.*, 2007 ▸), which can lead to changes in the drug’s properties. The desolvation process can produce either nonsolvated forms or hydrates, as water for hydrate formation can be obtained from the natural humidity of the surrounding environment (Bhattacharya & Saha, 2013 ▸; Minkov *et al.*, 2014 ▸; Nicolaï *et al.*, 2007 ▸). On the other hand, in some cases, the use of solvates as intermediates can be advantageous (Bhattacharya & Saha, 2013 ▸), as certain solvated forms can lower processing temperatures, aiding in techniques like hot-melt extrusion, as demonstrated for carbamazepine dihydrate (Williams *et al.*, 2021 ▸).

Up to now, six crystal structures of venetoclax have been reported in the literature. The structure of venetoclax monohydrate was published by Perdih *et al.* (2021 ▸). Venetoclax acetonitrile solvate, venetoclax fumarate acetonitrile solvate, venetoclax oxalate acetonitrile solvate, venetoclax napsylate acetonitrile solvate and venetoclax tosylate were recently published by our group (Havlůjová *et al.*, 2025 ▸), together with the description of ten fully characterized solvent-free salts with dicarboxylic and sulfonic acids. In addition, a lipophilic venetoclax docusate intended for enhanced lipid solubility has been described (Koehl *et al.*, 2022 ▸). To the best of our knowledge, no studies of the pure API crystalline forms have been published.

The aim of this study was to prepare and characterize polymorphs of venetoclax since none had been reported previously, and no crystal structures of pure venetoclax have been reported. After crystallization from various solvents, all our samples turned out to be solvated. Therefore, we have performed desolvation experiments and obtained two solvent-free crystalline forms. Computational methods were employed to understand how the crystal structure influences bulk properties. The *CSD-Particle* module in the *Mercury* software predicts particle shape and surface facets, offering insights into mechanical and chemical properties. *Solvate Analyser* allows the investigation of complex solvated structures, aiding in selecting suitable solvents and understanding their stability. To gain insight into the crystal structures of the solvent-free polymorphs, we performed spectroscopic analyses and compared them with the solvated forms. Together with computational methods, we obtained comprehensive information about the desolvation behaviour of the solvates and how the structures are affected by the transformation to solvent-free forms.

## 2. Materials and methods

### 2.1. Materials

Venetoclax was provided by Zentiva k.s. as an amorphous material. The solvents were obtained from various suppliers and used as supplied without any modifications.

### 2.2. Solvate screening

Seven different solvents, acetone, acetonitrile, 1,4-dioxane, ethyl acetate, isopropyl acetate, methyl ethyl ketone and 2-methyl tetrahydrofuran, were selected based on their polarity (Koehl *et al.*, 2022 ▸) and boiling point (Koehl *et al.*, 2022 ▸). 400 mg of venetoclax was dissolved in the appropriate amount of each solvent (Table 1 ▸) to form a clear solution at room temperature. All solutions were left open in the fume hood until visible precipitation occurred.

### 2.3. Desolvation

All samples were gently homogenized with a mortar and pestle and placed in a thin layer on Petri dishes of appropriate sizes. Based on preliminary experiments, the samples were then dried at 60°C under reduced pressure (20 mBar) for 24 h. The resulting powders were then analysed by nuclear magnetic resonance and X-ray powder diffraction (XRPD). If the drying proved insufficient (stoichiometric ratio of API:solvent ≥ 1:0.1), the procedure was repeated. The samples were then analysed by NMR, DSC, TGA and XRPD.

### 2.4. X-ray powder diffraction

The diffraction patterns were collected with a powder diffractometer device X’PERT PRO MPD PANalytical; X-ray beam Cu *K*α (λ = 1.542 Å), 5–40° 2θ measured range, 45 kV excitation voltage, 40 mA anodic current, 0.016° 2θ step size, 0.3 s time step. Measurement was carried out on a flat sample with an area/thickness ratio equal to 10/0.5 mm. The 2.5° Soller slits with a fixed slit width of 0.6 mm and automatic anti-scatter slits were used to correct the primary beam. The irradiated area of the sample was 10 mm. The secondary optics consisted of 2.5° Soller slits and 5.0 mm anti-scattering slits to correct the secondary beam. The detector used was LYNXEYE_XE_T (1D mode). The *HighScore Plus* software was used to process the diffraction patterns.

### 2.5. Solution ^1^H NMR

Samples were dissolved in d_6_-DMSO and ^1^H NMR spectra were measured by a Bruker Avance Neo 500 NMR spectrometer equipped with a Prodigy probe and a repetition delay of 10 s.

### 2.6. Solid-state NMR

^13^C and ^15^N CP-MAS solid-state NMR spectra were obtained using a Bruker Avance III 400 WB spectrometer equipped with a 4 mm probe and with 13 kHz spinning.

### 2.7. Thermogravimetric analysis

The samples were weighed in an aluminium pan (10 mg). All the measurements were performed on the TGA 6 (PerkinElmer, USA). The range of investigated temperatures was from room temperature (approximately 20°C) to 200°C with a heating rate of 10°C min^−1^.

### 2.8. Differential scanning calorimetry

The samples were weighed in an aluminium pan (10 mg). The pan was covered and the measurement was carried out under a nitrogen gas flow of 50 ml min^−1^. All measurements were performed on the TA Instruments Discovery DSC. The range of investigated temperatures was 0 to 300°C with a heating rate of 10°C min^−1^.

### 2.9. Fourier transform infrared spectroscopy

FTIR spectra were measured with a Nicolet IS50 FT-IR spectrometer using ZnSe single-bounce ATR. The measurement range was 400–4000 cm^−1^ and the resolution was 4 cm^−1^. All spectra shown are normalized and given as percentages, 0% is defined as the smallest value and 100% as the largest value in the dataset.

### 2.10. Intrinsic dissolution rate

The intrinsic dissolution rate (IDR) was determined using a Sirius inForm device (Pion Inc. USA). The 6 mm-diameter IDR discs were prepared by direct compression of approximately 50 mg of venetoclax. The material was compressed at a constant load of 100 kg, relaxed for 1 min and compressed again at the same pressure for another minute. IDR measurements were performed in 50 ml of phosphate buffer solution (pH 6.8) with the addition of 0.1% sodium dodecyl sulfate at a stirring speed of 100 rpm. UV spectra were recorded every 30 s using a probe with an optical path length equal to 20 mm^2^. The absorbance between wavelengths of 300–500 nm was used to evaluate the amount of API released at each time point. The IDR was obtained from the linear fit of first 10 min of the measurement. The first three points were excluded because they usually represent the dissolution of the free powder captured on the disc surface during the preparation.

### 2.11. Single-crystal X-ray diffraction

Single crystals were grown by slow evaporation of the solvent under ambient conditions. The venetoclax solvate analysis was performed at either 95 K or 120 K using a SuperNova diffractometer with a sealed microfocus tube, mirror-collimated Cu *K*α radiation (λ = 1.542 Å) and a CCD detector. Data reduction and absorption correction were carried out with the *CrysAlisPro* software (Baker, 2023 ▸). Structures were solved by charge-flipping methods using the *Superflip* software and refined by full-matrix least squares on squared values using the *Crystals* and *Jana2006* (Petříček *et al.*, 2014 ▸) software. The *MCE* software was used for the visualization of residual electron density maps (Rohlíček & Hušák, 2007 ▸). The hydrogen atoms were all located on a difference map, but those attached to carbon atoms were repositioned geometrically. The hydrogen atoms were initially refined with soft restraints on the bond lengths and angles to regularize their geometry (C—H in the range 0.93–0.98, N—H in the range 0.86–0.89, N—H to 0.86 and O—H = 0.82 Å) and *U*_iso_(H) (in the range 1.2–1.5 × *U*_eq_ of the parent atom), after which the positions were refined with riding constraints (Betteridge *et al.*, 2003 ▸).

Almost all the structures experienced some type of disorder. In one case (IPAC solvate), it was the API molecule that was disordered [disorder in the C(6) ring with occupancies 0.7:0.3]. In all of the other structures, there was a disorder in the solvent positions/conformations. The MEK solvate is a 2:1 solvate and both positions of the solvent were disordered. One was explicitly described with occupancies 0.928:0.072. For the other, this was not possible, and the solvent molecule was described with larger thermal ellipsoids. Dioxane solvate is a 4:1 solvate with one of the solvent positions disordered. The occupancies were 0.566:0.434. The mTHF solvate is a 2:1 solvate, with disorder at one of the solvent sites. Two main positions were successfully modelled with occupancies 0.591:0.409, but it is likely that more overlapped positions were present with very low occupancy. These were impossible to model. To obtain a stable refinement, we restrained the ADPs of this molecule to be similar. The EA solvate is a channel solvate with a heavily disordered solvent, probably in many positions within the channel. All attempts to explicitly model the solvent molecules failed. The *PLATON SQUEEZE* procedure (Spek, 2015 ▸) had to be used. The structure of the acetone solvate was twinned (Cooper *et al.*, 2002 ▸). All of the structures have some residual electron density in the vicinity of the chlorine atom, which is a common occurrence for halogen atoms.

The structures were compared using *CrystalCMP* (Rohlíček *et al.*, 2016 ▸; Rohlíček & Skořepová, 2020 ▸) software.

*CSD-Particle* (Moldovan & Maloney, 2024 ▸; Macrae *et al.*, 2008 ▸): morphologies and lattice energies were determined using the attachment energy method as implemented in *VisualHabit* (Clydesdale *et al.*, 1991 ▸). The calculations employed the Dreiding II force field with Gasteiger charges and a limiting radius of 30 Å. The surface chemistry and topology of the calculated facet morphology were examined by surface analysis (Bryant *et al.*, 2019 ▸).

**Figure 2:**
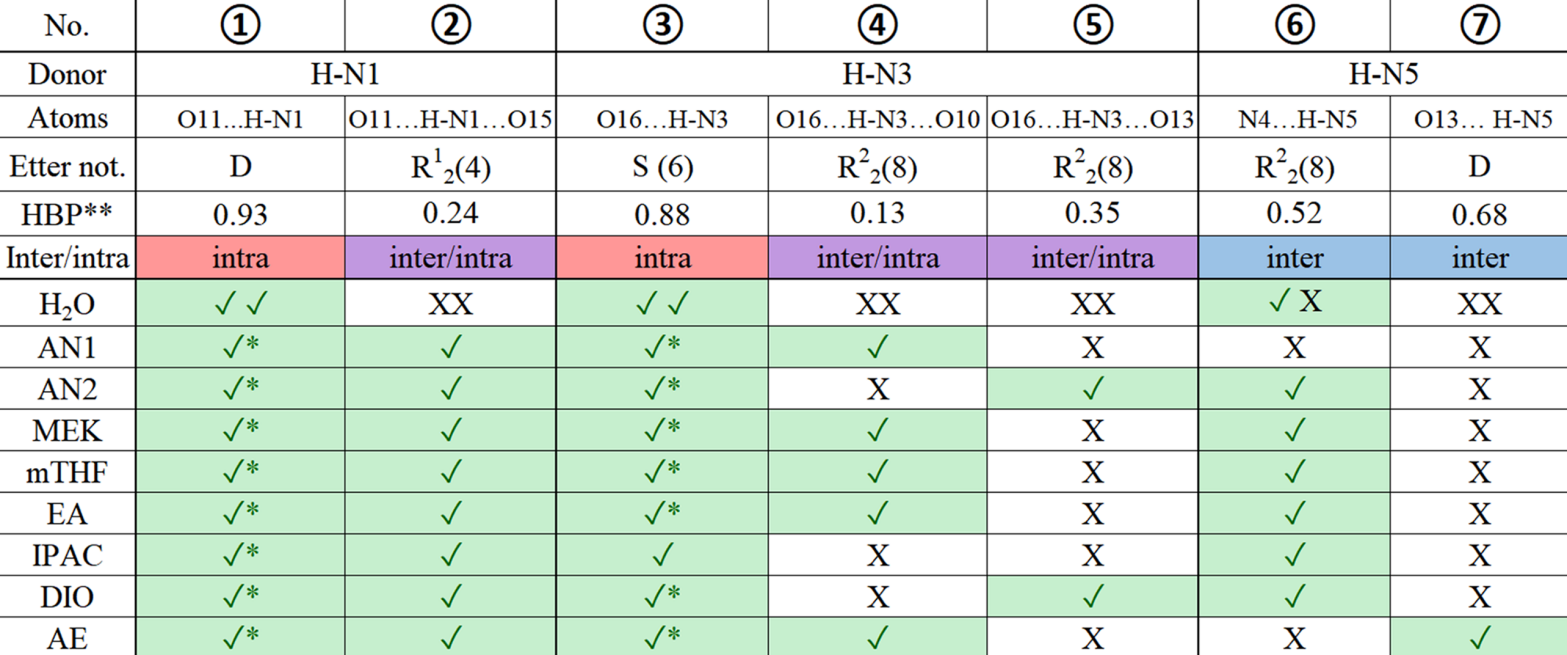
Hydrogen bonds in the venetoclax structures: (tick) present; (tick*) present, but only as a part of 3-centric hydrogen bond; (cross) absent; (HBP**) hydrogen bonding propensity.

*Hydrogen-bond propensity calculations* (Rohlíček *et al.*, 2016 ▸; Rohlíček & Skořepová, 2020 ▸): all seven venetoclax solvate crystal structures (VEN AN2, VEN MEK, VEN mTHF, VEN EA, VEN IPAC, VEN DIO and VEN AE), together with the previously published acetonitrile solvate (VEN AN1), were prepared for hydrogen-bond propensity (HBP) analysis in *Mercury*’s *CSD-Materials* module. In order to simulate the tool’s intended application to polymorph comparison, all solvent molecules were deleted prior to calculation; this poses no risk to our analysis since, in every solvate, the solvent is held only by van der Waals contacts and never participates in any hydrogen bond. Furthermore, any crystallographic disorder was manually modelled out (retaining only the major-occupancy positions) so as to ensure a single, well defined set of donor and acceptor sites. The HBP statistical model was then generated from the CSD (release 2024), using default donor–acceptor definitions and a 0.25 Å tolerance on heavy-atom distances. For each possible synthon in our structures, a propensity score was calculated and tabulated (Fig. 2 ▸). Model performance during training was assessed by receiver–operator characteristic analysis, yielding an area under the curve of 0.8495, indicating good discrimination between observed and non-observed hydrogen-bond patterns. The same logistic regression model (calculated for VEN EA) was used for each structure.

## 3. Results and discussion

**Figure 3:**
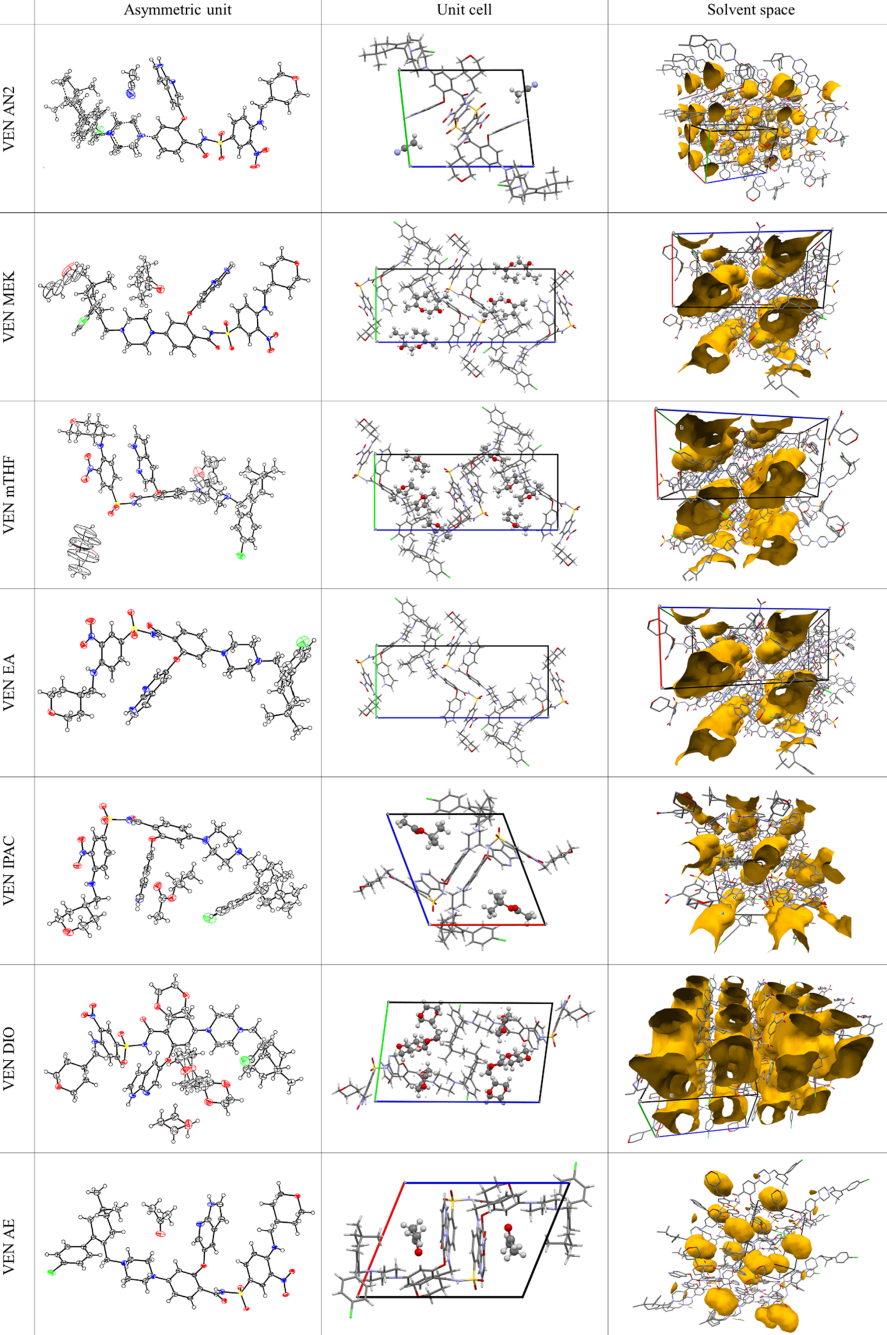
Crystal structures of venetoclax solvates.

As the starting material was amorphous and all the partially evaporated samples contained visible crystals, single-crystal X-ray diffraction was our primary characterization method. However, none of the measured samples contained only venetoclax, instead, we observed respective solvent molecules incorporated into each crystal lattice. Therefore, seven crystal structures of venetoclax solvates were solved. They were VEN AN2 (polymorph 2 of acetonitrile solvate), VEN MEK (methyl ethyl ketone solvate), VEN mTHF (2-methyl tetrahydrofuran solvate), VEN EA (ethyl acetate solvate), VEN IPAC (isopropyl acetate solvate), VEN DIO (dioxane solvate) and VEN AE (acetone solvate). All crystallographic details can be found in Table S1 of the supporting information. Selected parameters are shown in Table 2 ▸. Figures depicting the asymmetric units with the thermal ellipsoids, the unit cell with highlighted solvent molecules and the shapes of the voids in the structures occupied by the solvent are shown in Fig. 3 ▸.

VEN AN2 is a new polymorphic structure of venetoclax acetonitrile solvate. While it is similar to VEN AN1 (Havlůjová *et al.*, 2025 ▸), described previously, in both symmetry and unit-cell parameters, it exhibits a distinct calculated XRPD pattern. Additionally, the conformation of venetoclax and its molecular packing in the polymorphs are different. In VEN AN2, there is one molecule of API and one molecule of solvent in the asymmetric unit. The molecules of AN are bonded with only weak interactions and occupy discrete cavities in the structure. The solvent takes up 7.8% of the volume of the crystal.

Three of the novel solvates, VEN MEK, VEN mTHF and VEN EA, are isomorphous (similarities in VEN conformation and packing will be discussed further in the text). For VEN MEK, VEN mTHF, one molecule of API and two molecules of the solvent are present in the asymmetric unit. For VEN EA, the solvent cannot be modelled explicitly, but we can assume the same ratio based on the theoretical volumes of the molecules. Solvent molecules occupy channels running along the *b* direction in all structures. The solvent occupies between 21.5 and 25.3% of the volume of the crystal in these structures. To support the structural assignment of the VEN EA solvate, additional analysis was performed to estimate its true solvent content, given the extensive disorder that prevented explicit modelling of the ethyl acetate molecule in the crystal structure. Thermogravimetric analysis (TGA), differential scanning calorimetry (DSC) and solution ^1^H NMR were used (see Fig. S10 and Table S3 of the supporting information). TGA indicated a mass loss of 4.1%, corresponding to approximately 0.4 molar equivalents of ethyl acetate. Solution ^1^H NMR measurements suggested a slightly higher value, around 0.5 equivalents. These results consistently place the VEN:EA ratio in the range of 1:0.4–0.5. In contrast, the *PLATON SQUEEZE* procedure identified 202 electrons in the solvent-accessible voids of the unit cell, which would correspond to approximately four molecules of ethyl acetate (48 electrons each) and thus a 1:1 VEN:EA ratio (*Z* = 4). This discrepancy can be attributed to the sample handling conditions: while the single crystal used for SCXRD was flash-cooled directly from the mother liquor to preserve its fully solvated state, the bulk material used for TGA and NMR was filtered and gently dried under nitrogen to remove the adsorbed solvent. The reduced solvent content in the latter reflects the weak interaction between ethyl acetate and the venetoclax framework, which results in a high tendency toward partial desolvation during even minimal handling. These findings confirm that VEN EA is a low-stability solvate with a high desolvation propensity.

In the VEN IPAC solvate, there is one molecule of API and one molecule of solvent in the asymmetric unit. In the structure, solvent molecules occupy channels running along the *a* direction. The solvent takes up 17.1% of the volume of the crystal.

In the VEN DIO solvate there is one molecule of API and four molecules of the solvent in the asymmetric unit. Solvent molecules occupy 2D channels running along the *ab* plane. The solvent takes up 35.5% of the volume of the crystal.

VEN AE solvate has a 1:1 ratio of API to the solvent. VEN AE is a cavity solvate and the solvent takes up 8.1%.

The two previously published structures of venetoclax, monohydrate (VEN H_2_O) (Perdih *et al.*, 2021 ▸) and acetonitrile solvate (VEN AN1) (Havlůjová *et al.*, 2025 ▸) both have a 1:1 ratio of API to solvent. Both structures are cavity solvates, and the solvent takes up 4.5 and 7.9%, for the hydrate and VEN AN1, respectively.

**Figure 4:**
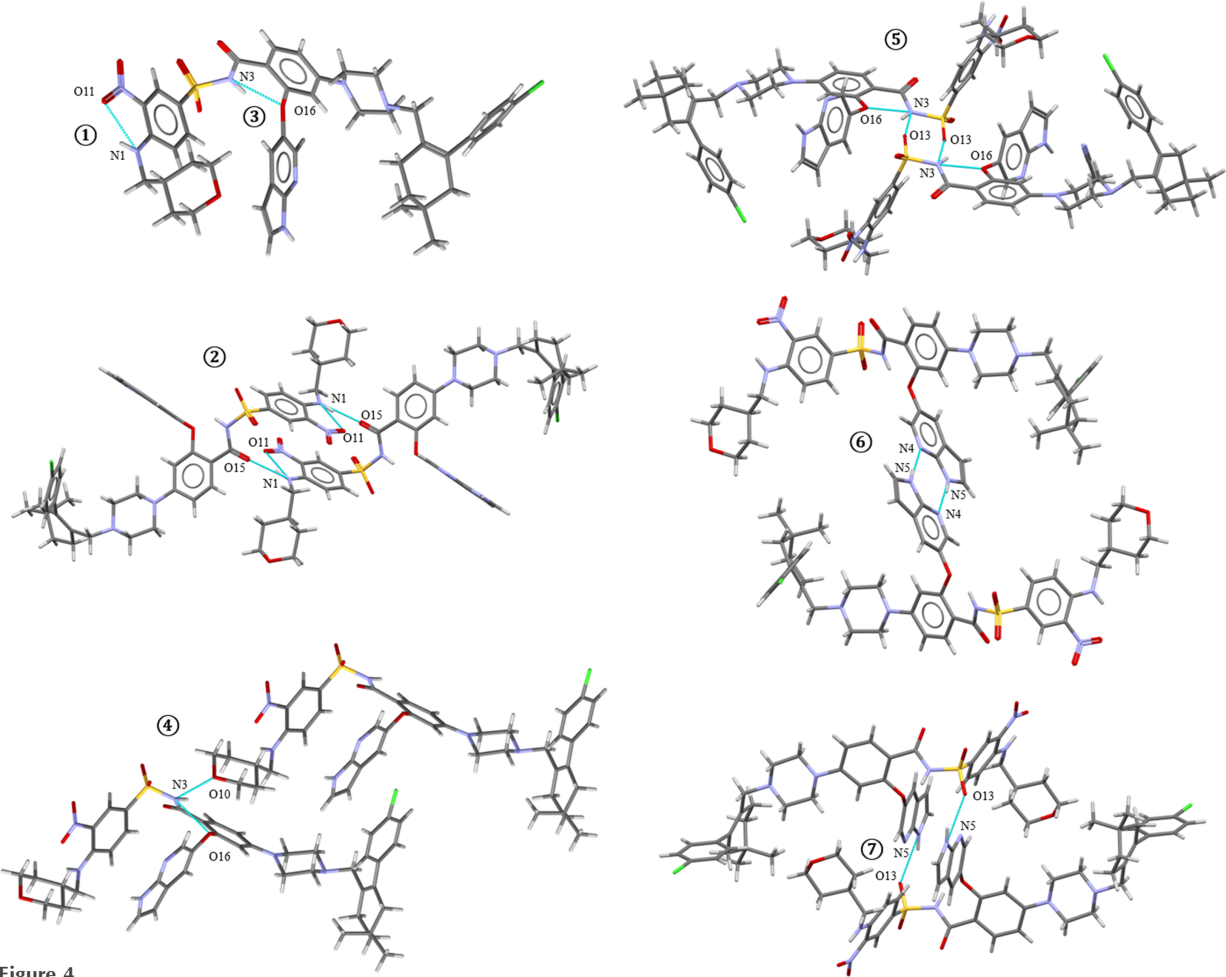
Hydrogen-bonding patterns in the solvates of venetoclax.

Hydrogen-bonding patterns in the structures are very complex due to the high number of both donor and acceptor groups. To analyse the trends in hydrogen bonding among the structures, all possible hydrogen bonds were identified and are listed in Fig. 2 ▸, which also shows hydrogen bonds present in each structure. The table is split into three parts based on the donors. The structure of the hydrate contains two molecules of VEN in the asymmetric unit, therefore, two marks for each type of interaction are present in the table. The corresponding hydrogen-bond patterns are shown in Fig. 4 ▸.

We can see that two intramolecular bonds marked as (1) and (3) are present in all the structures of venetoclax. These hydrogen bonds often form more complex, bifurcated 3-centric interactions by also involving an acceptor from a neighbouring molecule, observed in all structures except the hydrate. In the case of the amine H-N1, there are two options. It can either form an intramolecular hydrogen bonding with O11, marked as (1) in Fig. 4 ▸; or O15 can also contribute to this interaction, resulting in a 3-centric hydrogen bond, marked (2) in Fig. 4 ▸. For the amide H—N3, there are several options. An intramolecular hydrogen bond with ether O16, (3) in Fig. 4 ▸, forms in all structures; however, it is often combined with bonding either to tetrahydropyran O10 or to sulphoxyle O13, resulting in 3-centric hydrogen bonds (4) and (5) in Fig. 4 ▸. The N4⋯H—N5 intermolecular homosynthon of 7-azaindole, (6) in Fig. 4 ▸, is also notable, as it is present in 70% of cases. The most commonly occurring molecular interactions, numbers (2), (5) and (6), will be analysed in more detail. The other possible hydrogen bonds are less universal and appear only in some of the structures. With the exception of water in the hydrate, all solvent molecules are held only by weak interactions.

The three most common hydrogen bonds were analysed with respect to their interaction energy and its components using the *VisualHabit* calculation in *Mercury*, which determines the synthon energies (*i.e.* the energy of interaction between two whole molecules) with constituent parts being the hydrogen-bond energy, van der Waals energy and electrostatic energy. The values corresponding to the structure of VEN AN2 are shown in Table 3 ▸. The N4⋯H—N5 7-azaindole homosynthon, marked (6) in Table 3 ▸, is the strongest hydrogen-bond interaction present in the structure (highest hydrogen-bond energy component). However, the dimers based on the 3-centric hydrogen bonds marked (2) and (5) in Table 3 ▸ represent the two strongest VEN–VEN interactions in the whole structure. While both of these are relatively weak interactions and not typically significant in crystallographic terms, the molecules arrange themselves in such a way that the aromatic parts are able to align and interact strongly with one another. The aromatic interactions contributing to this arrangement include parallel offset π–π stacking of nitrobenzenes in one instance and aromatic interactions between 7-azaindole and nitrobenzene in another instance, characterized by herringbone offset π–π stacking. These interactions play a crucial role in stabilizing the structure by enhancing the overall binding between the molecules, leading to a highly stable crystal lattice.

To complement our structural and energetic characterization of venetoclax solvates, we have used *Mercury*’s *H-Bond Propensity* (HBP) tool to score every possible donor–acceptor interaction (Fig. 2 ▸). In simplified terms, the HBP value for each synthon is connected to its statistical likelihood, based on CSD occurrence, of appearing in the solid state. In our system, the scores span a wide range:

*Intramolecular hydrogen bonds* [(1), (3)]:

H—N1⋯O11 [(1)] carries the highest propensity, 0.93, reflecting its ubiquity and strong geometric preference for forming a five-membered ring.

H—N3⋯O16 [(3)] likewise scores very high, 0.88, consistent with its universal presence in all ten structures.

*Bifurcated, 3-centred hydrogen bonds* [(2), (4), (5)]:

The amine bifurcation O11⋯H—N1⋯O15 [(2)] is very unlikely (0.24), in line with CSD statistics that disfavour secondary acceptor involvement in this synthon.

The 3-centred amide options are both low to moderate: O16⋯H—N3⋯O10 [(4)] 0.13, O16⋯H—N3⋯O13 [(5)] 0.35, indicating only a minority of known structures employ these extended motifs.

*Intermolecular hydrogen bonds of the azaindole donor* [(6), (7)]:

The 7-aza homosynthon N4⋯H—N5 [(6)] scores 0.52, marking it as a reasonably common and favourable intermolecular network.

The alternative synthon O13⋯H—N5 [(7)] is even more probable (0.68), yet curiously observed only in VEN AE.

**Figure 5:**
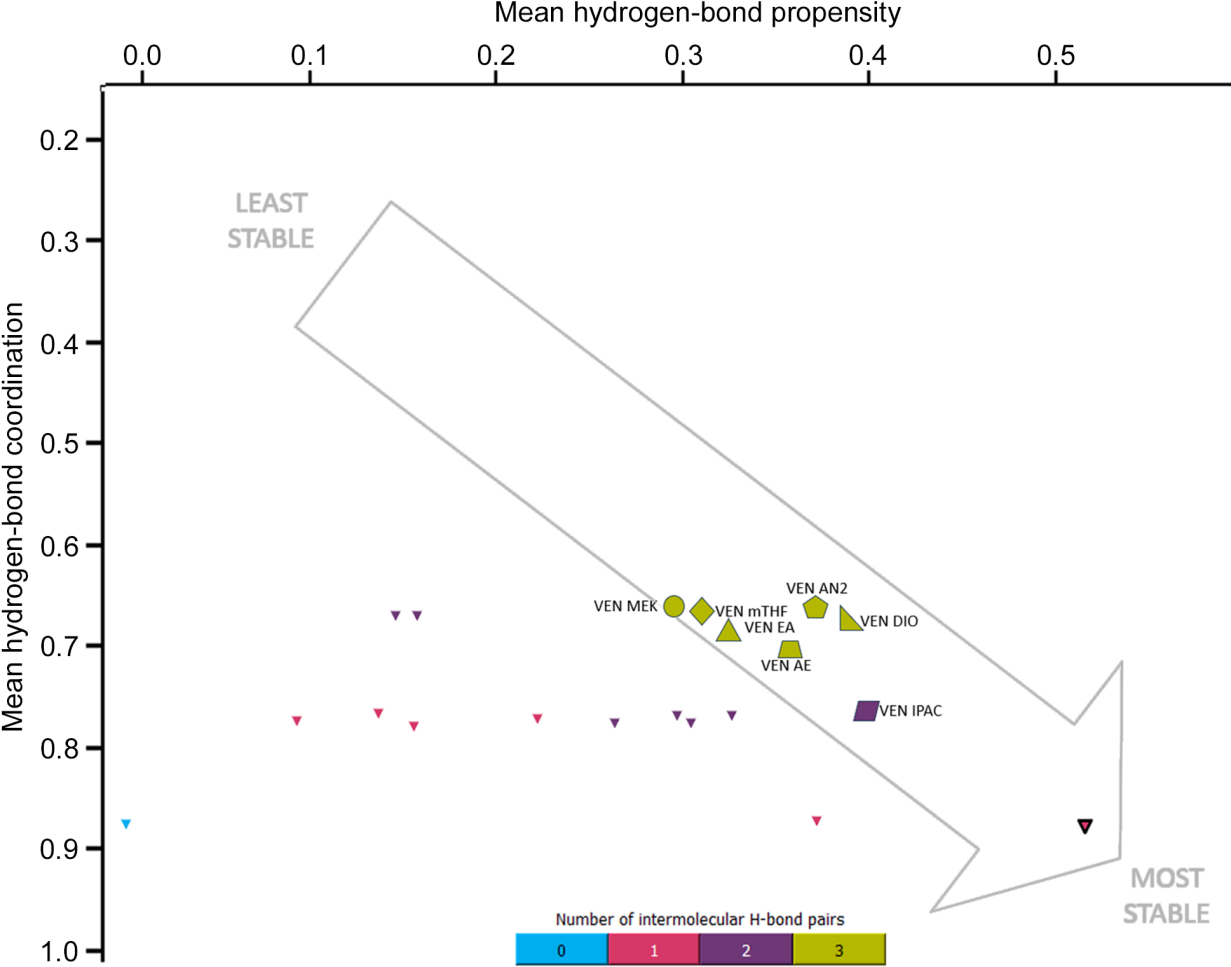
Hydrogen-bond propensity landscape of venetoclax.

The HBP landscape generated in *Mercury*’s *CSD-Materials* module (Fig. 5 ▸) plots the mean HBP on the horizontal axis against the mean hydrogen-bond coordination (HBC) on the vertical axis. Each point corresponds to one of the top-scoring predicted hydrogen-bond networks (smaller triangles) or to an experimentally observed solvate structure (larger symbols, labelled). Networks clustering toward the lower-right corner (high HBP, high HBC) are predicted to be the most favourable in the solid state. The highest-scoring network (HBP = 0.518, HBC = 0.878) involves a single intermolecular N4⋯H—N5 homosynthon (6). In this motif, the other two NH donors form only intramolecular hydrogen bonds and avoid bifurcation. This simple network achieves the best possible coordination because each donor and acceptor participates in exactly one optimal interaction, in line with CSD statistics that disfavour bifurcated hydrogen bonds for these functional groups. Among the seven experimental solvates, six (green shapes) each present three intermolecular hydrogen-bond pairs, two of which are 3-centred bifurcated interactions and, accordingly, plot at more moderate HBP/HBC values. The isopropyl acetate solvate (VEN IPAC, purple parallelogram) is unique: it has only two intermolecular hydrogen-bond pairs (one 3-centred synthon) and no second bifurcation, and it accordingly appears closest of the observed structures to the lower-right ‘most stable’ region. Thus, while our solvates frequently exploit low-probability bifurcated synthons [(2), (4), (5)] to enable favourable π–π stacking and accommodate solvent channels, the HBP analysis highlights that simpler homosynthons – particularly the single N4⋯H—N5 motif – would be intrinsically more favourable if the molecule could adopt that arrangement without compromising other non-covalent interactions.

Fig. S1 shows the calculated XRPD patterns related to the crystal structures of venetoclax solvates, including the previously published monohydrate (VEN-H_2_O) (Perdih *et al.*, 2021 ▸) and VEN AN1 (Havlůjová *et al.*, 2025 ▸). The patterns observed for the isomorphous group containing VEN MEK, VEN EA and VEN mTHF are very similar to each other while the rest differ significantly.

This difference arises from differences in the crystal structures. To explore this, the structures have been compared in two respects: venetoclax conformation and its molecular packing.

**Figure 6:**
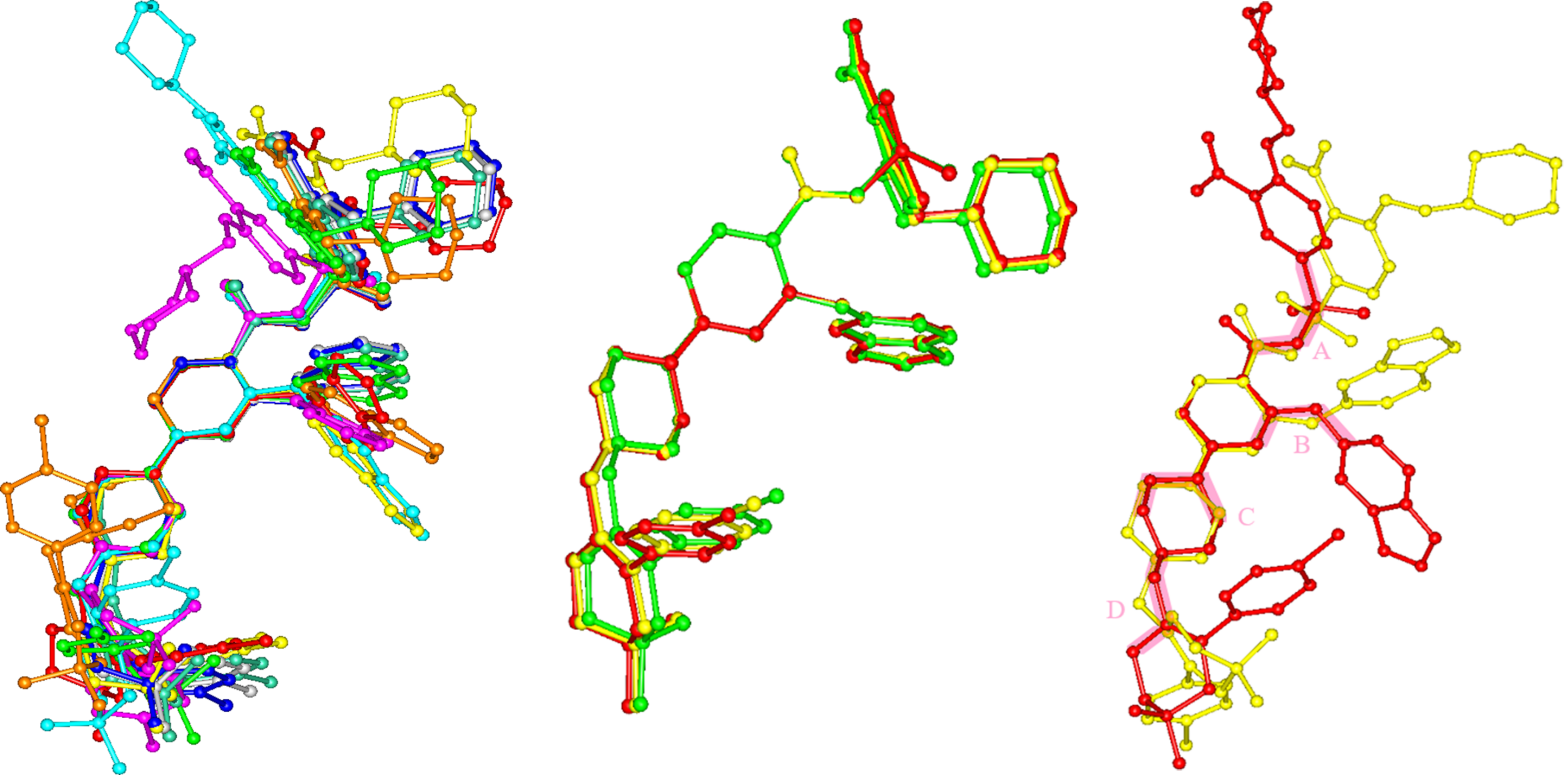
Comparison of the venetoclax conformation in its available structures. Left – all; middle – VEN EA, VEN MEK and VEN mTHF; left – VEN hydrate and VEN EA, with highlighted torsion causing the largest differences in the conformation. Overlay done over the central benzene ring.

All of the available structures (VEN H_2_O, VEN AN1, VEN AN2, VEN MEK, VEN mTHF, VEN EA, VEN IPAC, VEN DIO and VEN AE) were analysed with the help of the *CrystalCMP* (Bryant *et al.*, 2019 ▸; Macrae *et al.*, 2020 ▸) software. It compares the structures and creates similarity dendrograms (Figs. S2 and S3). The intramolecular hydrogen bonds discussed above affect the molecular conformation of venetoclax, basically anchoring some of the torsion angles. Even with this type of stabilization, when all of the structures of venetoclax are overlaid (left in Fig. 6 ▸), we can see that VEN is still very conformationally flexible and the torsion angles unaffected by molecular bonding lead to a variety of conformations.

Three structures that have an almost identical conformation of VEN can be identified (VEN MEK, VEN mTHF, VEN EA; middle in Fig. 6 ▸), not surprisingly, these three have been confirmed as isostructural. The rest are different from each other. The right part of Fig. 6 ▸ showcases the torsions causing the largest difference in the molecular conformations of VEN. It is an overlay of two least-similar conformations based on the PS_*ab*_ similarity score (based on RMSD), in VEN hydrate and VEN EA. Four torsions are highlighted, where there are the most significant differences: A – sulfonyl amide region, B – ether O16 region, C – piperazine region and D – torsion between the piperazine and cyclohexene rings.

**Figure 7:**
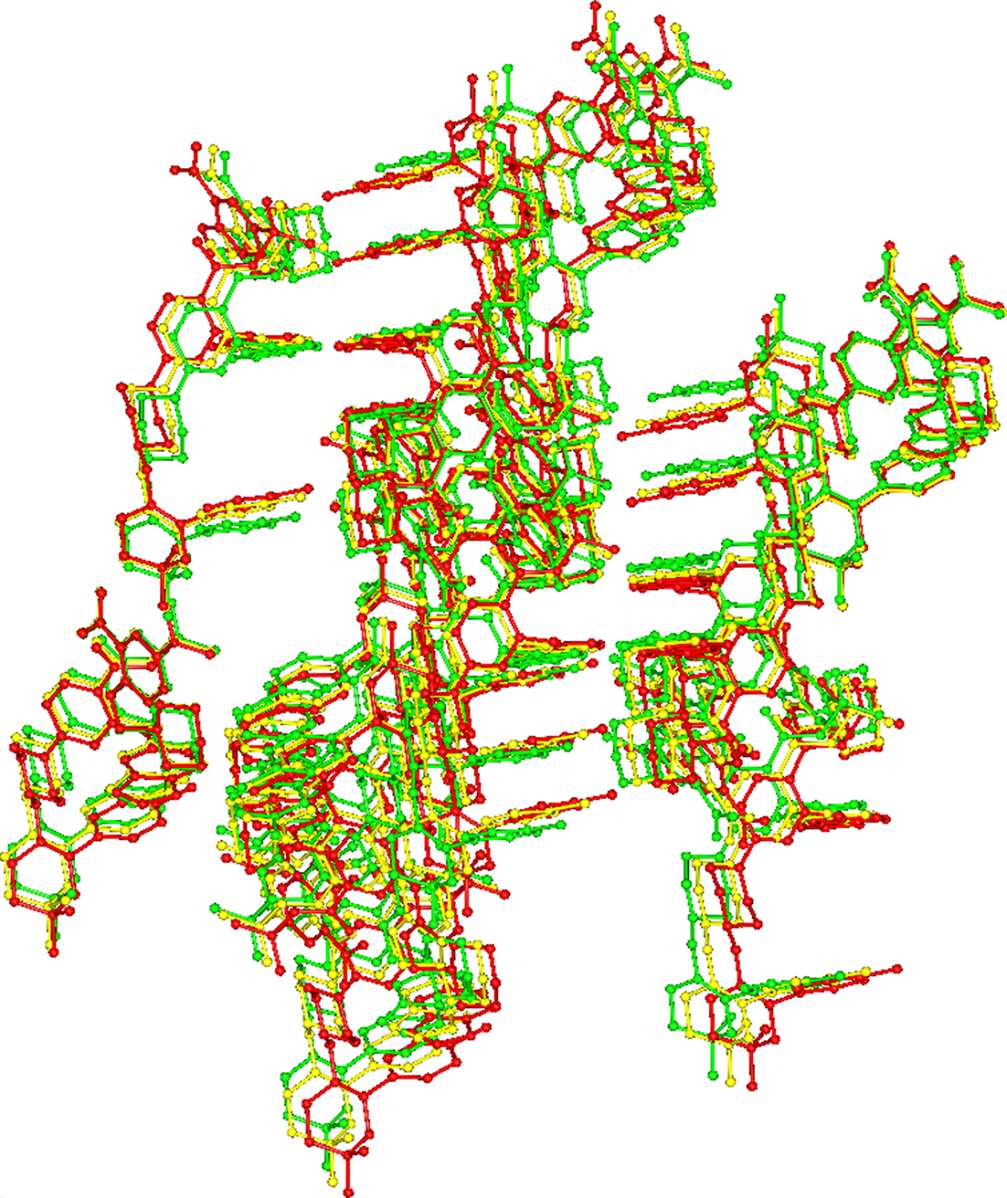
Comparison of the venetoclax molecular packing in VEN EA, VEN MEK and VEN mTHF.

A similar trend can also be observed for the VEN molecular packing. As can be seen in Fig. S1, VEN EA, VEN MEK and VEN mTHF are isostructural with respect to the venetoclax scaffold, and VEN AN1 and VEN AE are similar, but to a lesser degree (Figs. S4–S5). All of the other structures of venetoclax have a distinct packing of the VEN molecules. Fig. 7 ▸ shows the almost perfect overlay of the VEN EA and VEN MEK and VEN mTHF structures.

### 3.1. Desolvation

The second part of our study focused on the desolvation of the prepared solvates. All samples were dried under fairly harsh conditions (20 mBar, 60°C, up to 2 days). Gentler conditions were also tested, but they resulted in only partial desolvation.

The samples were analysed by XRPD, solution ^1^H NMR and thermal methods. Three different outcomes were observed:

(i) No transformation: the acetone (VEN AE) and dioxane (VEN DIO) solvates remained essentially intact under even our harshest drying conditions (20 mbar, 60°C, 48 h), with VEN AE releasing only ∼10% of its solvent and VEN DIO undergoing only partial desolvation. The exceptional robustness of VEN AE likely stems from its cavity-type topology, which physically hinders solvent escape, coupled with its unique HBP profile: HBP analysis shows that VEN AE is the only solvate not exhibiting the 7-azaindole homosynthon [(6), HBP = 0.52], instead adopting a higher-propensity dimeric N5–H⋯O_sulfonyl synthon [(7), HBP = 0.68]. Since the acetone guests engage only in weak van der Waals contacts, this strongly preferred hydrogen-bond motif effectively ‘locks in’ the venetoclax conformation and prevents the molecular reorganization required for complete desolvation.

(ii) Transformation to solvent-free venetoclax form A (VEN A). This is the case of VEN EA, MEK, mTHF, IPAC.

(iii) Transformation to solvent-free venetoclax form B (VEN B). This applies to VEN AN2.

Therefore, two solvent-free crystalline forms of venetoclax were identified. The samples were then characterized by XRPD, ^1^H NMR, TGA/DSC and IDR.

The melting points of the forms VEN A and B are 145.6 and 169.5°C, respectively. The DSC/TGA plots can be found in Fig. S6. ^1^H NMR confirmed that VEN A was fully solvent-free but that VEN B contained traces of acetonitrile (0.09 mol eq.). The transcripts of the spectra are available in the supporting information.

In some respects, the dissolution properties are the most important characteristic of novel pharmaceutical solid forms. Therefore, we have performed IDR measurements. For IDR, the studied powder is compressed into a disc with a known surface. Using this type of analysis, we obtain information about the dissolution rate of the investigated solid form without any potential effect of differing particle sizes. The IDR values of VEN A and B are comparable (11.7 and 12.1 µg min^−1^ cm^−2^). On comparison of post-dissolution discs to the starting material, no phase transitions were observed.

The aim of our work was to prepare non-solvated forms of venetoclax and to describe their crystal structures. However, all recrystallization attempts to create single crystals lead to solvate structures. This left us with the powder samples obtained by desolvation. Unfortunately, their crystallinity is too imperfect for a structure solution from powder data. Most of the described structures of venetoclax have triclinic symmetry, so there is a strong possibility that VEN A and B do too. This makes matters even worse and a standard structure solution from XRPD data is impossible. To bypass this obstacle and to obtain at least some information about the crystal structures of VEN A and B, we have employed a combination of calculations and spectroscopic methods. The next section explores the relationships between the parent solvates (with known structures) and the desolvates.

### 3.2. Desolvation behaviour and probable structure of desol­vates

**Figure 8:**
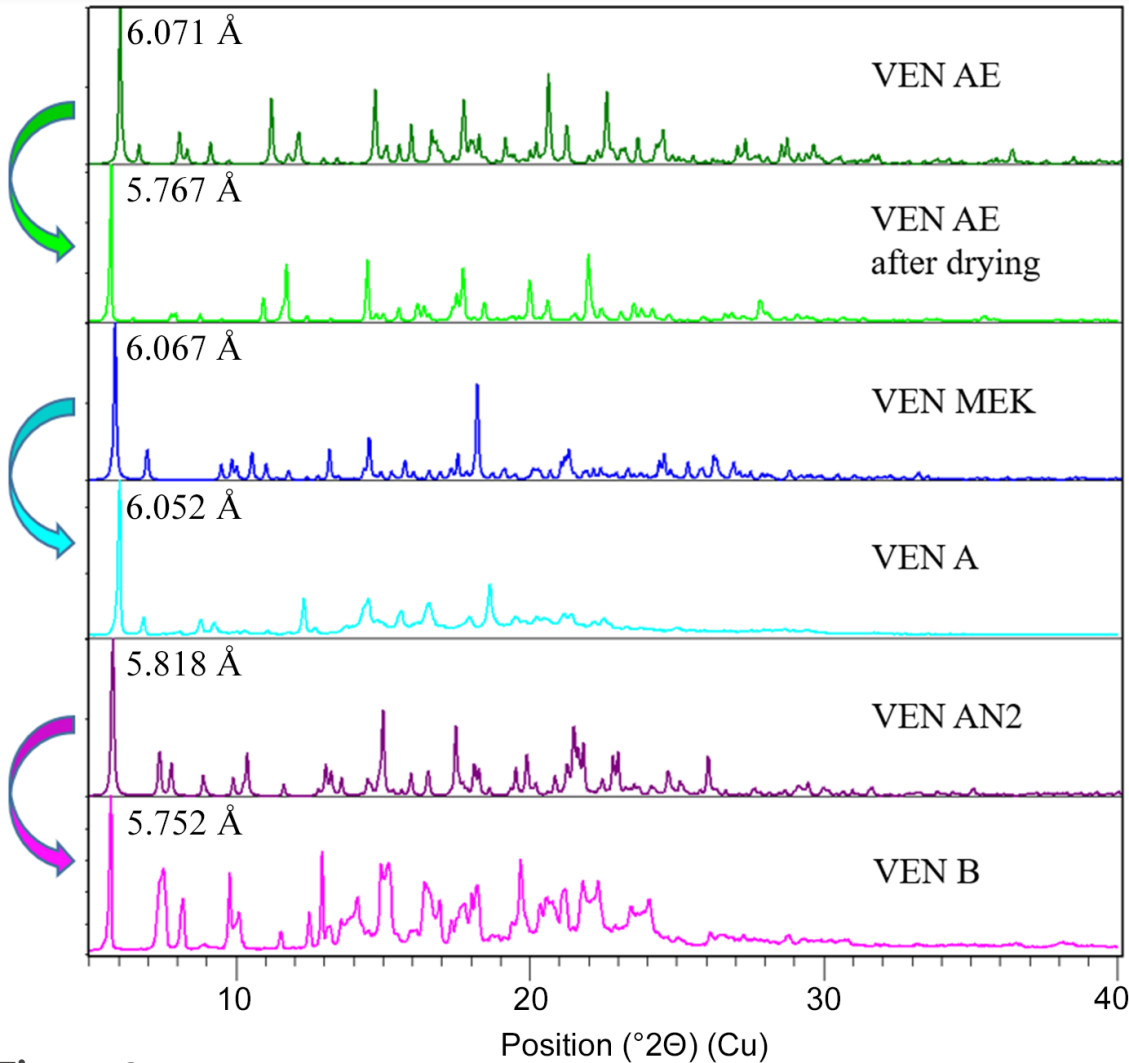
Powder diffractograms of the representative solvates and of the forms after drying. The interplanar distance corresponding to the first strong peak at room temperature is labelled (refer to Table 4 ▸).

As stated above, three types of outcomes were observed after drying the solvates: (i) the solvate was stable and did not desolvate, (ii) desolvation to VEN A, and (iii) desolvation to VEN B. For each of these, a representative crystal structure of a solvate was selected, with VEN AE (acetone solvate), VEN MEK (methyl ethyl ketone solvate) and VEN AN2 (aceto­nitrile solvate polymorph 2) selected for cases (i), (ii) and (iii), respectively. Fig. 8 ▸ shows the XRPD patterns corresponding to these cases.

The crystal structures of the desolvated VEN A and B are not available. Comparison of XRPD patterns shows some structural changes upon desolvation, but the overall molecular arrangement likely remains similar to the parent solvates, as indicated by the visual similarity of the diffractograms. If we consider this to be true, we can analyse the data further by evaluating the interplanar distances. Table 4 ▸ shows the data that refer to the first strong peak (all at room temperature). VEN AE, which partially desolvates upon drying, but does not transform, experiences the largest shrinkage of 5% in the (001) direction. The other two cases exhibit a very small difference, meaning that the VEN molecules probably rearrange/change conformation in such a way that it does not significantly affect the interplanar spacing in the (001) direction.

To understand the structural features related to the desol­vation behaviour of venetoclax solvates, a detailed computational analysis was performed by combining two tool sets within the *Mercury* software, the *Solvate analyser* and the *CSD-Particle* suite.

First, an attachment model of the crystal based on Dreiding synthon energies was created using the *VisualHabit* software. Table 5 ▸ shows the obtained lattice energy as well as the energy of the interaction between venetoclax and solvent molecules (the sum of synthon energies with values above −1 kJ mol^−1^; for MEK divided by 2, since it is a disolvate).

The acetone solvate, VEN AE, has the highest lattice energy and the highest interaction energy between venetoclax and solvent molecules, corresponding to its high stability and resistance to desolvation. The methyl ethyl ketone solvate, VEN MEK, which transforms to VEN A upon desolvation and is a channel solvate, has the lowest lattice energy. The acetonitrile solvate polymorph 2, VEN AN2, which transforms to VEN B upon desolvation and is a discrete cavity solvate, has a medium lattice energy slightly lower than VEN AE. The interaction energies for MEK and AN are comparable.

**Figure 9:**
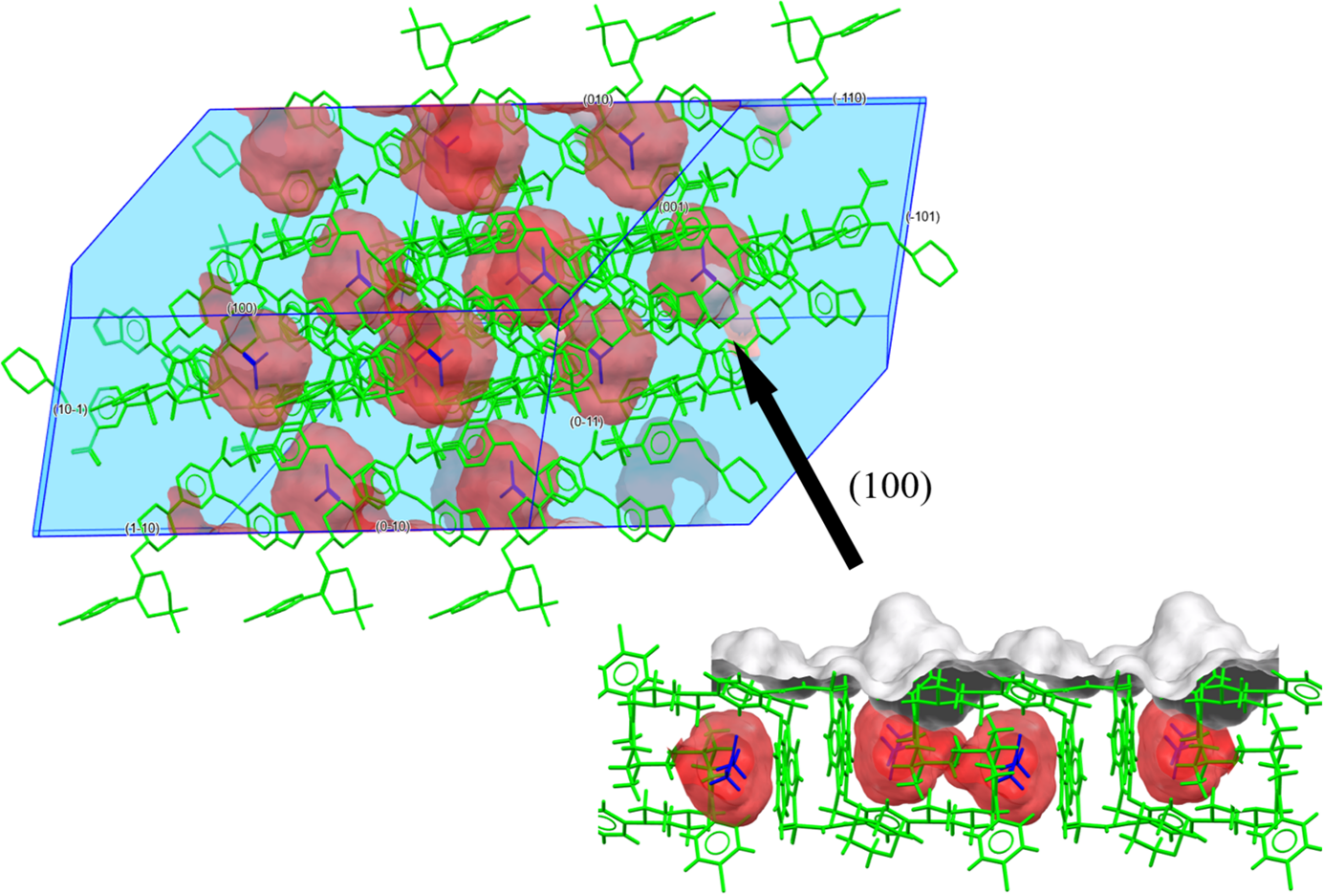
VEN AE: *Solvate Analyser* images for the whole crystal and for the largest crystal face (100).

Next, we analysed the crystal surface properties and solvate behaviour of the three venetoclax solvates. These analyses were conducted using the *Solvate Analyser*, and surface properties were examined using *CSD-Particle*. The largest crystal facet of each solvate was examined, as it provides the most significant contribution to the overall stability and behaviour of the crystal structure. Figs. 9 ▸–11 ▸ ▸ show the calculated crystal shape (obtained in *VisualHabit*) for each of the solvates and the topology of the main crystal surface (obtained in *Analyse Surface*), each of them overlapped with the map of the solvent-occupied space (obtained in *Solvate Analyser*).

The calculated crystal habit of VEN AE is an elongated prism. This solvate remained stable without desolvation upon drying and this stability can be attributed to the strong intermolecular interactions within the crystal lattice, particularly on the largest crystal face (100). The hydrogen-bond acceptor count (0.033) and donor count (0.009) suggest a balanced hydrogen-bonding network, with a moderate aromatic bond count (0.056) reflecting stable π–π stacking interactions. The solvent occupies 8.1% of the crystal space, which is relatively low and contributes to the stability of the solvate. The *Solvate Analyser* images illustrate the cavity-type character of this solvate. The view of the surface molecules provides further evidence of the stability of this solvate, as we can see that each solvent molecule is enclosed within a venetoclax cage, creating a stable, well ordered surface with relatively low rugosity (1.632) and a high attachment energy (−73.936 kJ mol^−1^).

The calculated crystal habit of VEN MEK is a thick needle with solvent channels running along the longest dimension of the crystal. Analysis of the largest crystal face (101) revealed solvent channels running along this face, which likely facilitate the removal of solvent molecules, leading to desolvation for VEN A. The *Solvate Analyser* images show these channels and the alignment along the (011) face where the channels end. The surface properties in Table 6 ▸ indicate a lower attachment energy (−36.057 kJ mol^−1^) compared with VEN AE, suggesting a less stable structure. The hydrogen-bond acceptor count (0.029) and donor count (0.005) are lower, indicating a less extensive hydrogen-bonding network. The lower aromatic bond count (0.034) points to weaker π–π stacking interactions, contributing to the less stable nature of VEN MEK. The solvent occupies 22.9% of the crystal space, which is relatively high and supports the channel-type solvates’ tendency to desolvate easily. The channel-type nature of VEN MEK means that the necessary changes in the conformation and orientation of venetoclax molecules during desolvation could be more minor, facilitating easier desolvation to VEN A.

The calculated crystal habit of VEN AN2 is a block-like prism. The *Solvate Analyser* images illustrate the cavity-type character of this solvate. The largest crystal face (001) displayed significant aromatic interactions. The surface properties from Table 6 ▸ indicate a moderate attachment energy (−66.08 kJ mol^−1^) and the highest rugosity (2.36) among the three solvates, reflecting a complex surface topology. The hydrogen-bond acceptor count (0.054) and donor count (0.013) suggest a highly interactive hydrogen-bonding network. The higher aromatic bond count (0.094) indicates strong π–π stacking interactions, enhancing the stability of the acetonitrile solvates’ surface. The solvent occupies only 7.8% of the crystal space, which is relatively low and supports the cavity-type solvates’ stability. Upon desolvation, this solvate transforms into VEN B, which is more thermodynamically stable than VEN A based on the melting points. From the calculation results and the cavity-type character of the solvate, we can infer that a significant reordering of venetoclax molecules can be expected during desolvation, requiring larger conformational and orientational changes.

**Figure 12:**
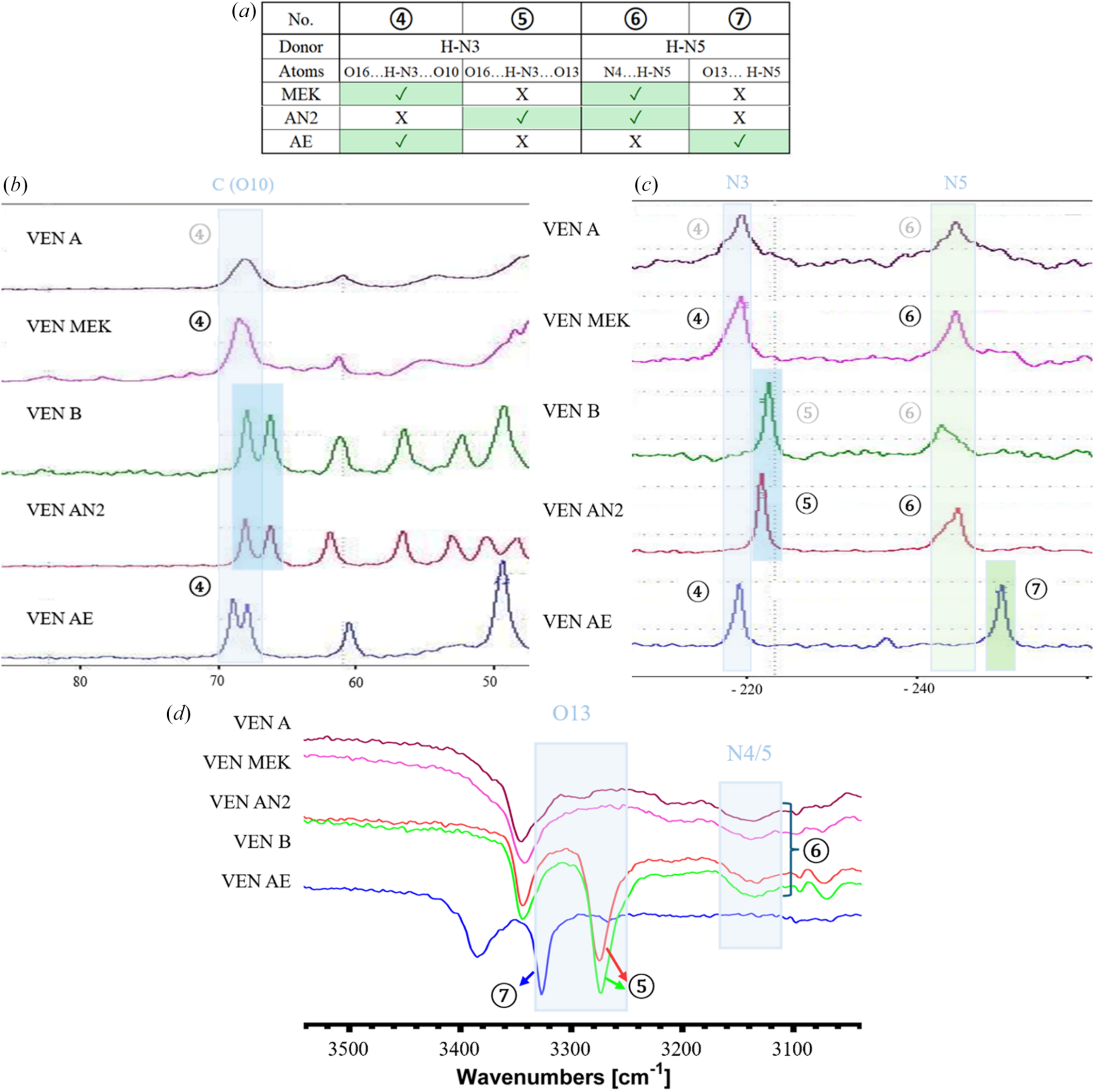
Spectroscopic methods and venetoclax interactions, selected regions: (*a*) hydrogen bonds, where VEN AE, VEN MEK and VEN AN2 differ; (*b*) ^13^C ssNMR; (*c*) ^15^N ssNMR; (*d*) FTIR. Relevant atoms/groups are highlighted and particular hydrogen-bonding types are indicated by circled numbers.

To explore the close-range order and interactions in the solvates and desolvates and their similarities and dissimilarities, solid-state spectroscopic methods were employed. FTIR gives complex information about the functional groups in the molecules and their interactions. Solid-state NMR, namely ^15^N and ^13^C, tells us about the particular atoms in the molecule and about their more or less immediate surroundings. The spectra from all of these three methods for VEN A, VEN MEK, VEN B, VEN AN2 and VEN AE are shown in Figs. 12 ▸ and S7–S9, with highlighted relevant atoms/groups. From all three methods, it is apparent that the desolvated forms are very similar to the parent solvates. VEN A spectra are similar to VEN MEK spectra and VEN B spectra are similar to VEN AN2 spectra. The pairs are distinctly different from each other and also from the VEN AE solvate. To explore the differences and similarities, we have focused on the atoms and functional groups involved in hydrogen bonding, which we know in detail from the crystal structures of the solvates (see Figs. 2 ▸ and 4 ▸ discussed above). Since we know which particular interactions occur in the solvates, we can perhaps deduce information about the interactions and molecular arrangement of venetoclax in the desolvated forms (where we do not have the crystal structures). Fig. 12 ▸ includes (*a*) a table showing which hydrogen-bonding interactions (exhibiting differences between investigated solvate structures) are present in the selected solvate crystal structures marked with circled numbers referencing Fig. 4 ▸, and also the most relevant parts of the spectra from (*b*) ^13^C ssNMR, (*c*) ^15^N ssNMR and (*d*) FTIR.

In ^13^C ssNMR, it is very evident that VEN A and VEN MEK have poor crystallinity compared with the other forms (the spectra are weaker and have much broader peaks). We have focused on the two carbon atoms adjacent to O10 in the tetrahydropyran ring. In solution NMR, these would be equivalent, but due to the anisotropy of the solid state, they appear here as two close/overlapping peaks. O10 accepts a hydrogen bond (4) in VEN MEK and VEN AE, but not in VEN AN2. Indeed, in the spectra, we see a distinct difference in the chemical shifts. If hydrogen bond (4) is present, the peaks are shifted to higher p.p.m. Based on this information, we can say that it is likely that in VEN A, O10 participates in hydrogen bond (4), and in VEN B it does not.

The same trends, but on different hydrogen bonds, can be observed in ^15^N ssNMR. Here, we focused on two nitrogen atoms, N3 and N5, both of which are hydrogen-bond donors. N3-H is a sulphonamide NH involved in either hydrogen bond (4) or hydrogen bond (5), both of which are 3-centric hydrogen bonds, with one of the acceptors being O16 and the other either O10 or O13. There is the same grouping of spectra as for ^13^C ssNMR described above. Based on this information, we can deduce that in VEN A, N3 participates in hydrogen bond (4), while in VEN B, it is hydrogen bond (5). N5 is indole NH involved in either hydrogen bond (6) or hydrogen bond (7). N5-H is a donor in hydrogen bond (6) in VEN MEK and VEN AN2, and in hydrogen bond (7) in VEN AE. This is consistent with the chemical shifts, with only VEN AE having a lower one. Based on this information, we can deduce that, in both VEN A and VEN B, N5 participates in hydrogen bond (6).

FTIR spectroscopy was employed to confirm interactions in the VEN forms. Fig. 12 ▸(*d*) highlights regions which are mostly related to the bond of hydrogen and other atoms, *i.e.ca* 3500–3000 cm^−1^, as hydrogen-bond interactions are connected with the hydrogen atom bond vibration frequency change. In VEN B and VEN AN2, we see a strong signal (5) corresponding to the N—H stretching vibration of N3—H. This band position is strongly influenced by the interaction with O13 and O16, which might be confirmed by vibration band positions and shifts in the region around 1350 cm^−1^. Similarly, other bands highlighted in blue in Fig. 12 ▸(*d*) can be assigned to particular hydrogen atom bands influenced by hydrogen-bond interactions. FTIR spectra confirm results from the crystal structure analysis, *i.e.* in VEN AN2 there are hydrogen-bond interactions (5), and in VEN AE there are hydrogen-bond interactions (7). No hydrogen-bond interactions of O13 were found in VEN MEK. The azaindole N—H stretching vibrations in the region around 3350 cm^−1^ are the same in all forms except VEN AE. This again confirms crystal structure results that VEN MEK and VEN AN2 do exhibit the azaindole homosynthon hydrogen bond (6), in contrast to VEN AE.

All of the information obtained from the chosen spectral techniques is consistent with each other and also with the crystal structure analysis. Spectral techniques have proven that there are the same hydrogen bonds in VEN A as in VEN MEK, and there are the same hydrogen bonds in VEN B as in VEN AN2. The above-mentioned spectral analyses confirm that the crystal structures of the desolvates are very closely related to the structures of the parent solvates.

## 4. Conclusions

Our venetoclax crystallization screening identified seven solvates. Single-crystal X-ray diffraction revealed five of them to be channel solvates and two of them to be cavity solvates. Three of the channel solvates are isomorphous with nearly identical venetoclax molecular packing. Mapping our crystal structures onto an HBP landscape allowed us to rank each synthon’s statistical likelihood and actual coordination quantitatively. Interestingly, six of the seven solvates employ low-propensity bifurcated hydrogen-bond motifs – despite their unfavourable CSD scores – most likely because the slight conformational distortion needed to form them also enhances aromatic π–π stacking. The desolvation study identified two venetoclax forms: A (melting point 145.6°C) and B (169.5°C). IDR measurements showed that form B dissolves slightly faster than form A. In general, the channel solvates desolvated to venetoclax form A, while the two cavity solvates either did not desolvate at all (acetone solvate) or transformed to venetoclax form B (acetonitrile solvate). Because single crystals of forms A and B could not be obtained, we used a combination of computational and spectroscopic methods to gather structural insights. *Solvate Analyser* and *CSD-Particle* provided insights into the crystal surface properties and desolvation behaviour. Seven types of hydrogen-bonding patterns were identified among the solvate structures. Based on the comparison of FTIR, ^13^C ssNMR and ^15^N ssNMR spectra between the solvated and desolvated forms, we can be almost sure that the hydrogen bonds venetoclax molecules experience in the solvate structures remain undisturbed upon desolvation. It showed that both close-range and long-range molecular interactions and arrangements of venetoclax molecules are highly similar between the desolvated forms and the parent solvates. This knowledge led us to our currently ongoing efforts, where we are hoping to solve the crystal structures of solvent-free forms of venetoclax from powder data using the FIDEL refinement, which is a specialized procedure to refine a structure based on a model with significantly deviating unit-cell parameters.

## Supplementary Material

Zip containing all CIFs. DOI: 10.1107/S2052252525006785/yc5050sup1.zip

Supporting figures and tables. DOI: 10.1107/S2052252525006785/yc5050sup2.pdf

CCDC references: 2381780, 2381781, 2381782, 2381783, 2381784, 2381785, 2381786

## Figures and Tables

**Figure 10:**
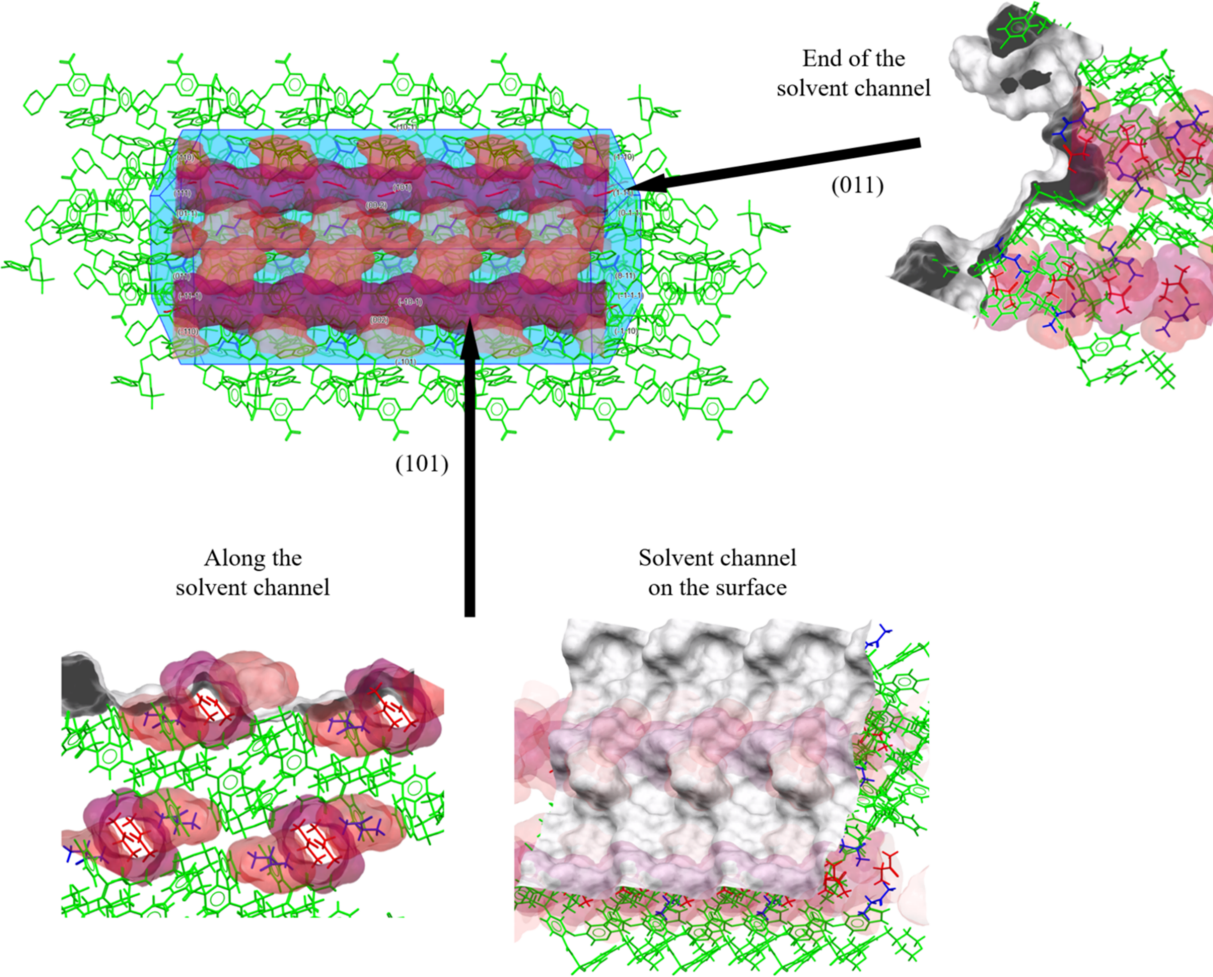
VEN MEK: *Solvate Analyser* images for the whole crystal and for the largest crystal face (101) along which run the solvate channels, and for the face (011), on which the channels end.

**Figure 11:**
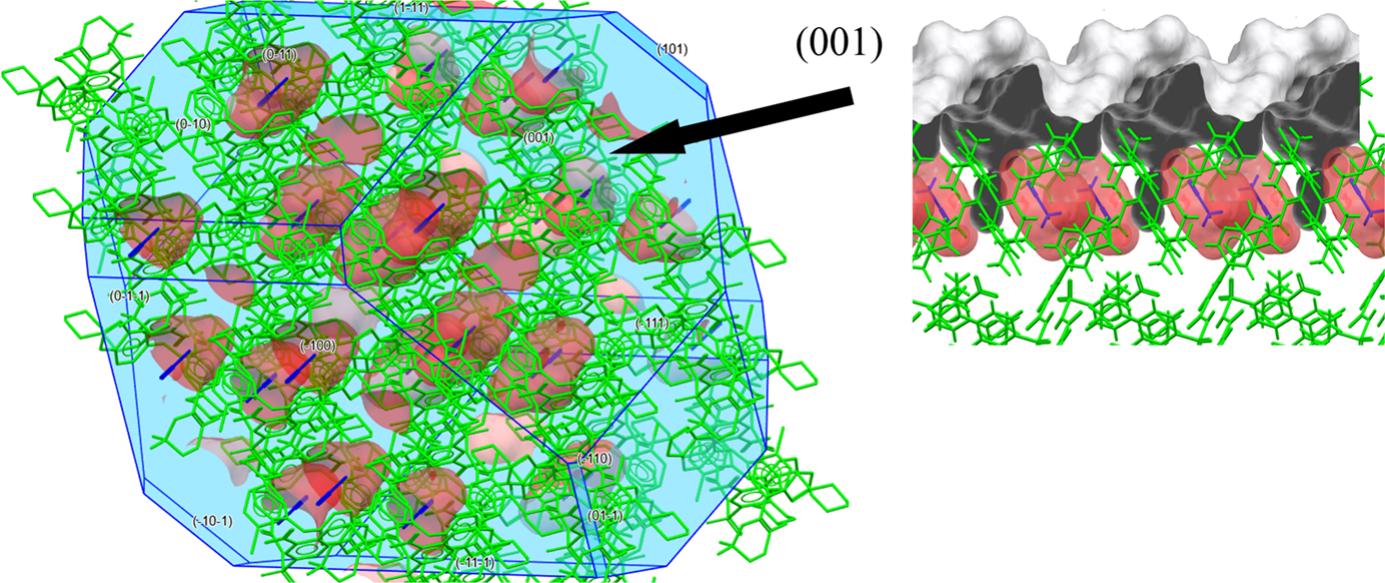
VEN AN2: *Solvate Analyser* images for the whole crystal and for the largest crystal face (001).

**Table 1:** Estimated solubility of venetoclax at room temperature (estimated by the addition of 5 ml aliquots until dissolution, the suspension was stirred for 30 s between each addition)

Solvent	Abbreviation	Estimated solubility (mg ml^−1^)	Polarity index (p′)	Boiling point (°C)
Acetone	AE	5	5.1	56.05
Acetonitrile	AN	2	5.8	81.6
1,4-Dioxane	DIO	5.6	4.8	101.1
Ethyl acetate	EA	3.3	4.4	77.1
Isopropyl acetate	IPAC	2.7	3.9	88.0
Methyl ethyl ketone	MEK	3	4.7	79.6
2-methyl tetrahydrofuran	mTHF	5.5	4.0	80.2

**Table 2:** Selected structural parameters

Crystal data	VEN H_2_O†	VEN AN1‡	VEN AN2	VEN MEK	VEN mTHF	VEN EA	VEN IPAC	VEN DIO	VEN AE
API:solvent ratio	1:1	1:1	1:1	1:2	1:2	NA	1:1	1:4	1:1
Crystal system, space group	Triclinic, *P*1	Triclinic, *P*1	Triclinic, *P*1	Monoclinic, *P*2_1_/*n*	Monoclinic, *P*2_1_/*n*	Monoclinic, *P*2_1_/*n*	Triclinic, *P*1	Triclinic, *P*1	Triclinic, *P*1
Temperature (K)	150	95	95	95	120	120	95	95	95
*a* (Å)	12.6058 (3)	12.2261 (5)	12.4625 (1)	14.2655 (1)	14.6928 (1)	13.7621 (1)	13.5842 (2)	10.6785 (4)	12.0250 (5)
*b* (Å)	13.6947 (3)	14.0822 (6)	13.1131 (2)	12.2567 (1)	12.3178 (1)	12.5121 (1)	13.6070 (2)	13.5301 (5)	13.6215 (6)
*c* (Å)	26.0490 (6)	15.1150 (5)	15.3650 (2)	30.0079 (2)	29.8810 (2)	29.9705 (3)	14.3796 (3)	21.9604 (8)	15.7475 (8)
α (°)	83.7790 (18)	67.388 (4)	93.2334 (11)	90	90	90	105.5089 (15)	82.660 (3)	93.425 (4)
β (°)	87.6244 (18)	72.278 (3)	96.3237 (10)	92.7510 (4)	93.2176 (6)	92.5017 (8)	109.2253 (16)	88.791 (3)	110.937 (5)
γ (°)	81.3877 (18)	74.731 (3)	113.9359 (12)	90	90	90	92.2405 (15)	80.515 (3)	102.318 (4)
*V* (Å^3^)	4418.55 (17)	2256.41 (17)	2266.90 (5)	5240.77 (7)	5399.43 (7)	5155.79 (8)	2394.59 (8)	3103.8 (2)	2327.4 (2)
*V*_solv_ (%)	4.5	7.9	7.8	22.9	25.3	21.5	17.1	35.5	8.1

† Perdih *et al.* (2021 ▸).

‡ Havlůjová *et al.* (2025 ▸).

**Table 3:** Interaction energies of the three most common hydrogen-bonding synthons

	Hydrogen-bond pattern		
	(2)	(5)	(6)
Interaction energy (kJ mol^−1^)	−38.561	−40.541	−30.069
Hydrogen-bond energy (kJ mol^−1^)	−7.19	−8.165	−25.391
van der Waals energy (kJ mol^−1^)	−29.337	−32.51	−3.577
Electrostatic energy (kJ mol^−1^)	−2.035	0.133	−1.101

**Table 4:** Shift of the first strong peak in XRPD on drying

	VEN AE	VEN AE (after drying)	VEN MEK	VEN A	VEN AN2	VEN B
First strong peak	(001)	(001)	(002)	NA	(001)	NA
Interplanar distance at room temperature (Å)	6.071	5.767	6.067	6.052	5.818	5.752
Difference (%)	5.01		0.25		1.13	

**Table 5:** Energy calculation results

	VEN AE	VEN MEK	VEN AN2
Lattice energy (kJ mol^−1^)	−197.89	−147	−189.959
VEN, solvent interaction energy (kJ mol^−1^)	−35.066	−30.768	−29.191

**Table 6:** Particle surface properties of the largest crystal face

	VEN AE	VEN MEK	VEN AN2
Miller indices	(100)	(101)	(001)
Percentage facet area (all equiv. faces)	28.854	32	30.948
Attachment energy (kJ mol^−1^)	−73.936	−36.057	−66.08
Rugosity	1.632	1.533	2.36
Hydrogen-bond acceptors (count Å^−2^)	0.033	0.029	0.054
Hydrogen-bond donors (count Å^−2^)	0.009	0.005	0.013
Hydrogen-bond donors unsatisfied (count Å^−2^)	0.005	0.005	0.007
Aromatic bonds (count Å^−2^)	0.056	0.034	0.094
