# Supplementary material for: Unravelling venetoclax solvate behaviour: insights from crystal structures and computational surface analysis
Source: IUCrJ. 2025 Aug 28;12(Pt 5):595–609. doi: 10.1107/S2052252525006785 (PMC12403167; doi:10.1107/S2052252525006785)

checkCIF/PLATON report

Structure factors have been supplied for datablock(s) I

THIS REPORT IS FOR GUIDANCE ONLY. IF USED AS PART OF A REVIEW PROCEDURE FOR PUBLICATION, IT SHOULD NOT REPLACE THE EXPERTISE OF AN EXPERIENCED CRYSTALLOGRAPHIC REFEREE.

No syntax errors found. CIF dictionary Interpreting this report

Datablock: I

Bond precision: C-C = 0.0042 A Wavelength=1.54180

Cell:	a=10.6785(4)	b=13.5301(5)	c=21.9604(8)
	alpha=82.660(3)	beta=88.791(3)	gamma=80.515(3)
Temperature:	95 K		

	Calculated	Reported
Volume	3103.8(2)	3103.8(2)
Space group	P -1	P -1
Hall group	-P 1	?
Moiety formula	C45 H50 Cl N7 O7 S, 4(C4 H8 O2)	C61 H82.00 Cl1 N7 O15 S1
Sum formula	C61 H82 Cl N7 O15 S	C61 H82.00 Cl1 N7 O15 S1
Mr	1220.85	1220.88
Dx,g cm-3	1.306	1.306
Z	2	2
Mu (mm-1)	1.451	1.451
F000	1300.0	1300.0
F000'	1305.32	
h,k,lmax	13,16,27	13,16,27
Nref	12710	12204
Tmin,Tmax	0.893,0.967	0.860,0.970
Tmin'	0.841	

Correction method= # Reported T Limits: Tmin=0.860 Tmax=0.970
AbsCorr = MULTI-SCAN

Data completeness= 0.960 Theta(max)= 74.609

R(reflections)= 0.0567(9486) wR2(reflections)= 0.1734(12204)

S = 0.989 Npar= 815

The following ALERTS were generated. Each ALERT has the format
test-name_ALERT_alert-type_alert-level.
Click on the hyperlinks for more details of the test.

Alert level C

```
DIFMN02_ALERT_2_C  The minimum difference density is < -0.1*ZMAX*0.75
            _refine_diff_density_min given =     -1.280
            Test value =     -1.275
DIFMN03_ALERT_1_C  The minimum difference density is < -0.1*ZMAX*0.75
            The relevant atom site should be identified.
PLAT029_ALERT_3_C _diffrn_measured_fraction_theta_full value Low .      0.960 Why?
PLAT041_ALERT_1_C Calc. and Reported SumFormula    Strings  Differ     Please Check
              Calc: C61 H82 Cl N7 O15 S
              Rep.: C61 H82.00 Cl1 N7 O15 S1
PLAT042_ALERT_1_C Calc. and Reported MoietyFormula Strings  Differ     Please Check
              Calc: C45 H50 Cl N7 O7 S, 4(C4 H8 O2)
              Rep.: C61 H82.00 Cl1 N7 O15 S1
PLAT220_ALERT_2_C NonSolvent   Resd 1  C   Ueq(max)/Ueq(min) Range        3.4 Ratio
PLAT250_ALERT_2_C Large U3/U1 Ratio for <U(i,j)> Tensor(Resd    2)        3.0 Note
PLAT250_ALERT_2_C Large U3/U1 Ratio for <U(i,j)> Tensor(Resd    3)        2.3 Note
PLAT250_ALERT_2_C Large U3/U1 Ratio for <U(i,j)> Tensor(Resd    5)        2.4 Note
PLAT340_ALERT_3_C Low Bond Precision on  C-C Bonds ..............    0.00421 Ang.
PLAT410_ALERT_2_C Short Intra H...H Contact  H191     ..H221     .       1.95 Ang.
                                                 x,y,z  =      1_555 Check
PLAT906_ALERT_3_C Large K Value in the Analysis of Variance ......      2.924 Check
PLAT911_ALERT_3_C Missing FCF Refl Between Thmin & STh/L=    0.600         72 Report
               -4 14  0,   8 14  0,   6 15  0,  -6-15  1,  -7-14  1,   4-14  1,
               -9-13  1,   6 15  1,   4-14  2,  -8-13  2,  -9-12  3, -10-11  4,
                3-14  5, -10-11  5, -11 -9  5,   3-13  9,   3  5 12,   2  6 12,
                3  3 13,   3  4 13,   2  5 13,   3  5 13,  10  9 13,   2 15 13,
                3  2 14,   3  3 14,   2  4 14,   9 10 14,   1 14 14,  10 -1 15,
                9  9 15,   8 10 15,   9 10 15,   8 11 15,   1 14 15,   2 14 15,
                8  9 16,   9  9 16,   7 10 16,   8 10 16,   7 11 16,   7 12 16,
                1 14 16,   2 14 16,  -4 -4 17,  -3 -4 17,   9 -2 17,   9  8 17,
                8 10 17,   7 11 17,   6 12 17,  -4 -4 18,  -3 -3 18,   9  7 18,
                5 12 18,  -5 -3 19,  -4 -3 19,   6 11 19,  -6 -3 20,  -7 -2 20,
               -6 -2 20,  -8 -1 20,  -8  0 20,  -7 -1 21,  -3  4 25,  -2  4 25,
               -1  4 25,   0  4 25,  -2  5 25,  -1  5 25,   0  4 26,   1  4 26,
PLAT934_ALERT_3_C Number of (Iobs-Icalc)/Sigma(W) > 10 Outliers ..          1 Check
               12  1  6,
```

Alert level G

```
PLAT002_ALERT_2_G Number of Distance or Angle Restraints on AtSite         22 Note
PLAT154_ALERT_1_G The s.u.'s on the Cell Angles are Equal ..(Note)      0.003 Degree
PLAT175_ALERT_4_G The CIF-Embedded .res File Contains SAME Records          1 Report
PLAT177_ALERT_4_G The CIF-Embedded .res File Contains DELU Records          1 Report
PLAT178_ALERT_4_G The CIF-Embedded .res File Contains SIMU Records          1 Report
PLAT188_ALERT_3_G A Non-default SIMU Restraint Value has been used     0.0100 Report
PLAT302_ALERT_4_G Anion/Solvent/Minor-Residue Disorder (Resd    2)        67% Note
PLAT398_ALERT_2_G Deviating  C-O-C   Angle From 120 for O62      .      109.3 Degree
PLAT398_ALERT_2_G Deviating  C-O-C   Angle From 120 for O65      .      109.8 Degree
PLAT398_ALERT_2_G Deviating  C-O-C   Angle From 120 for O74      .      109.5 Degree
PLAT398_ALERT_2_G Deviating  C-O-C   Angle From 120 for O77      .      109.5 Degree
PLAT398_ALERT_2_G Deviating  C-O-C   Angle From 120 for O80      .      109.8 Degree
```

```
PLAT398_ALERT_2_G Deviating  C-O-C   Angle From 120 for O83      .      109.4 Degree
PLAT720_ALERT_4_G Number of Unusual/Non-Standard Labels .........         16 Note
              H7301   H7302   H7211   H7212   H6901   H6902   H7001   H7002
              H7311   H7312   H7201   H7202   H6911   H6912   H7011   H7012
PLAT769_ALERT_4_G CIF Embedded Explicitly Supplied Scattering Data     Please Note
PLAT790_ALERT_4_G Centre of Gravity not Within Unit Cell: Resd.  #          3 Note
              C4 H8 O2
PLAT790_ALERT_4_G Centre of Gravity not Within Unit Cell: Resd.  #          4 Note
              C4 H8 O2
PLAT860_ALERT_3_G Number of Least-Squares Restraints ............        120 Note
PLAT910_ALERT_3_G Missing # of FCF Reflection(s) Below Theta(Min).          1 Note
                0  0  1,
PLAT912_ALERT_4_G Missing # of FCF Reflections Above STh/L=  0.600        432 Note
PLAT960_ALERT_3_G Number of Intensities with I  <   - 2*sig(I) ...          6 Check
PLAT969_ALERT_5_G The 'Henn et al.' R-Factor-gap value ..........       2.43 Note
              Predicted wR2: Based on SigI**2  7.15 or SHELX Weight 18.14
```

---

  0 **ALERT level A** = Most likely a serious problem - resolve or explain
  0 **ALERT level B** = A potentially serious problem, consider carefully
 14 **ALERT level C** = Check. Ensure it is not caused by an omission or oversight
 22 **ALERT level G** = General information/check it is not something unexpected

  4 ALERT type 1 CIF construction/syntax error, inconsistent or missing data
 13 ALERT type 2 Indicator that the structure model may be wrong or deficient
  9 ALERT type 3 Indicator that the structure quality may be low
  9 ALERT type 4 Improvement, methodology, query or suggestion
  1 ALERT type 5 Informative message, check

It is advisable to attempt to resolve as many as possible of the alerts in all categories. Often the minor alerts point to easily fixed oversights, errors and omissions in your CIF or refinement strategy, so attention to these fine details can be worthwhile. In order to resolve some of the more serious problems it may be necessary to carry out additional measurements or structure refinements. However, the purpose of your study may justify the reported deviations and the more serious of these should normally be commented upon in the discussion or experimental section of a paper or in the "special_details" fields of the CIF. checkCIF was carefully designed to identify outliers and unusual parameters, but every test has its limitations and alerts that are not important in a particular case may appear. Conversely, the absence of alerts does not guarantee there are no aspects of the results needing attention. It is up to the individual to critically assess their own results and, if necessary, seek expert advice.

Publication of your CIF in IUCr journals

A basic structural check has been run on your CIF. These basic checks will be run on all CIFs submitted for publication in IUCr journals (*Acta Crystallographica*, *Journal of Applied Crystallography*, *Journal of Synchrotron Radiation*); however, if you intend to submit to *Acta Crystallographica Section C* or *E* or *IUCrData*, you should make sure that full publication checks are run on the final version of your CIF prior to submission.

Publication of your CIF in other journals

Please refer to the *Notes for Authors* of the relevant journal for any special instructions relating to CIF submission.

PLATON version of 06/01/2024; check.def file version of 05/01/2024

Datablock I - ellipsoid plot

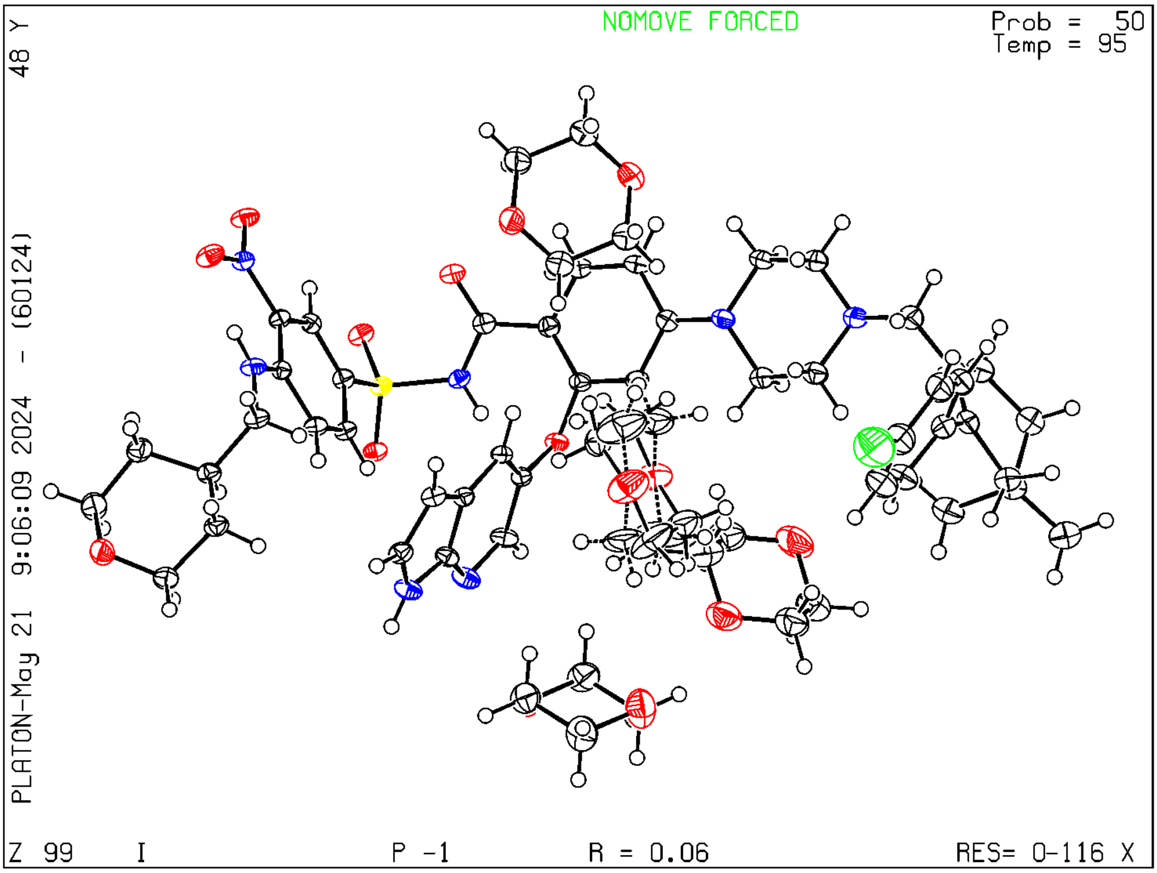

Supplement: Supplementary file 1 [file m-12-00595-sup1.zip › str for CCDC/ven diox final/checkcif.pdf]
