# Supplementary material for: Unravelling venetoclax solvate behaviour: insights from crystal structures and computational surface analysis
Source: IUCrJ. 2025 Aug 28;12(Pt 5):595–609. doi: 10.1107/S2052252525006785 (PMC12403167; doi:10.1107/S2052252525006785)

# checkCIF/PLATON report

Structure factors have been supplied for datablock(s) I

THIS REPORT IS FOR GUIDANCE ONLY. IF USED AS PART OF A REVIEW PROCEDURE FOR PUBLICATION, IT SHOULD NOT REPLACE THE EXPERTISE OF AN EXPERIENCED CRYSTALLOGRAPHIC REFEREE.

No syntax errors found.    CIF dictionary    Interpreting this report

# Datablock: I

---

Bond precision: C-C = 0.0025 A    Wavelength=1.54180

| | | | |
|---|---|---|---|
| Cell: | a=13.7621(1) | b=12.5121(1) | c=29.9705(3) |
| | alpha=90 | beta=92.5017(8) | gamma=90 |
| Temperature: | 120 K | | |

| | Calculated | Reported |
|---|---|---|
| Volume | 5155.79(8) | 5155.79(8) |
| Space group | P 21/n | P 21/n |
| Hall group | -P 2yn | ? |
| Moiety formula | C45 H50 Cl N7 O7 S [+ solvent] | C45 H50 Cl1 N7 O7 S1 |
| Sum formula | C45 H50 Cl N7 O7 S [+ solvent] | C45 H50 Cl1 N7 O7 S1 |
| Mr | 868.43 | 868.45 |
| Dx,g cm-3 | 1.119 | 1.119 |
| Z | 4 | 4 |
| Mu (mm-1) | 1.445 | 1.445 |
| F000 | 1832.0 | 1832.0 |
| F000' | 1839.99 | |
| h,k,lmax | 16,15,35 | 16,14,35 |
| Nref | 9288 | 9247 |
| Tmin,Tmax | 0.674,0.881 | 0.650,0.880 |
| Tmin' | 0.504 | |

Correction method= # Reported T Limits: Tmin=0.650 Tmax=0.880
AbsCorr = MULTI-SCAN

Data completeness= 0.996    Theta(max)= 67.553

R(reflections)= 0.0403( 8031) wR2(reflections)= 0.1095( 9246)

S = 0.961 Npar= 550

---

The following ALERTS were generated. Each ALERT has the format
**test-name_ALERT_alert-type_alert-level.**
Click on the hyperlinks for more details of the test.

---

## Alert level C

```
PLAT410_ALERT_2_C Short Intra H...H Contact  H161     ..H541     .      1.95 Ang.
                                                      x,y,z  =     1_555 Check
PLAT911_ALERT_3_C Missing FCF Refl Between Thmin & STh/L=    0.599        42 Report
              -13  9  1,  15  6  2, -15  6  5,  -5 14  7,  -7 13 10,  15  4 10,
               14  6 11, -13  8 12, -10 11 12,  -2 14 12, -11 10 13,   4 13 15,
               14  4 15, -14  5 16,  -4 13 16, -13  6 18,   5 12 18,  -5 12 19,
                4 12 19,  -4 12 20,  10  8 20,   9  8 22,  12  2 23, -10  7 24,
               -9  8 24,   1 11 24,   8  8 24,  -7  9 25, -11  4 26,   2 10 26,
               -8  7 27,   1  9 28,   2  9 28,   1  8 30,   7  3 31,  -4  6 32,
               -4  5 33,   4  4 33,  -6  0 34,  -3  4 34,   1  3 35,   3  0 35,
PLAT934_ALERT_3_C Number of (Iobs-Icalc)/Sigma(W) > 10 Outliers ..         1 Check
              -13  9  3,
```

---

## Alert level G

```
PLAT007_ALERT_5_G Number of Unrefined Donor-H Atoms .............          3 Report
              H231  H331  H441
PLAT041_ALERT_1_G Calc. and Reported SumFormula    Strings  Differ     Please Check
              Calc: C45 H50 Cl N7 O7 S
              Rep.: C45 H50 Cl1 N7 O7 S1
PLAT042_ALERT_1_G Calc. and Reported MoietyFormula Strings  Differ     Please Check
              Calc: C45 H50 Cl N7 O7 S
              Rep.: C45 H50 Cl1 N7 O7 S1
PLAT142_ALERT_4_G s.u. on b - Axis Small or Missing .............    0.00010 Ang.
PLAT606_ALERT_4_G Solvent Accessible VOID(S) in Structure ........         ! Info
PLAT769_ALERT_4_G CIF Embedded Explicitly Supplied Scattering Data     Please Note
PLAT860_ALERT_3_G Number of Least-Squares Restraints .............       760 Note
PLAT869_ALERT_4_G ALERTS Related to the Use of SQUEEZE  Suppressed         ! Info
PLAT909_ALERT_3_G Percentage of I>2sig(I) Data at Theta(Max) Still       77% Note
PLAT960_ALERT_3_G Number of Intensities with I  <   - 2*sig(I) ...         8 Check
PLAT969_ALERT_5_G The 'Henn et al.' R-Factor-gap value ..........       5.88 Note
              Predicted wR2: Based on SigI**2  1.86 or SHELX Weight 11.74
```

---

0 **ALERT level A** = Most likely a serious problem - resolve or explain
0 **ALERT level B** = A potentially serious problem, consider carefully
3 **ALERT level C** = Check. Ensure it is not caused by an omission or oversight
11 **ALERT level G** = General information/check it is not something unexpected

2 ALERT type 1 CIF construction/syntax error, inconsistent or missing data
1 ALERT type 2 Indicator that the structure model may be wrong or deficient
5 ALERT type 3 Indicator that the structure quality may be low
4 ALERT type 4 Improvement, methodology, query or suggestion
2 ALERT type 5 Informative message, check

---

It is advisable to attempt to resolve as many as possible of the alerts in all categories. Often the minor alerts point to easily fixed oversights, errors and omissions in your CIF or refinement strategy, so attention to these fine details can be worthwhile. In order to resolve some of the more serious problems it may be necessary to carry out additional measurements or structure refinements. However, the purpose of your study may justify the reported deviations and the more serious of these should normally be commented upon in the discussion or experimental section of a paper or in the "special_details" fields of the CIF. checkCIF was carefully designed to identify outliers and unusual parameters, but every test has its limitations and alerts that are not important in a particular case may appear. Conversely, the absence of alerts does not guarantee there are no aspects of the results needing attention. It is up to the individual to critically assess their own results and, if necessary, seek expert advice.

**Publication of your CIF in IUCr journals**

A basic structural check has been run on your CIF. These basic checks will be run on all CIFs submitted for publication in IUCr journals (*Acta Crystallographica*, *Journal of Applied Crystallography*, *Journal of Synchrotron Radiation*); however, if you intend to submit to *Acta Crystallographica Section C* or *E* or *IUCrData*, you should make sure that full publication checks are run on the final version of your CIF prior to submission.

**Publication of your CIF in other journals**

Please refer to the *Notes for Authors* of the relevant journal for any special instructions relating to CIF submission.

---

**PLATON version of 06/01/2024; check.def file version of 05/01/2024**

**Datablock I** - ellipsoid plot

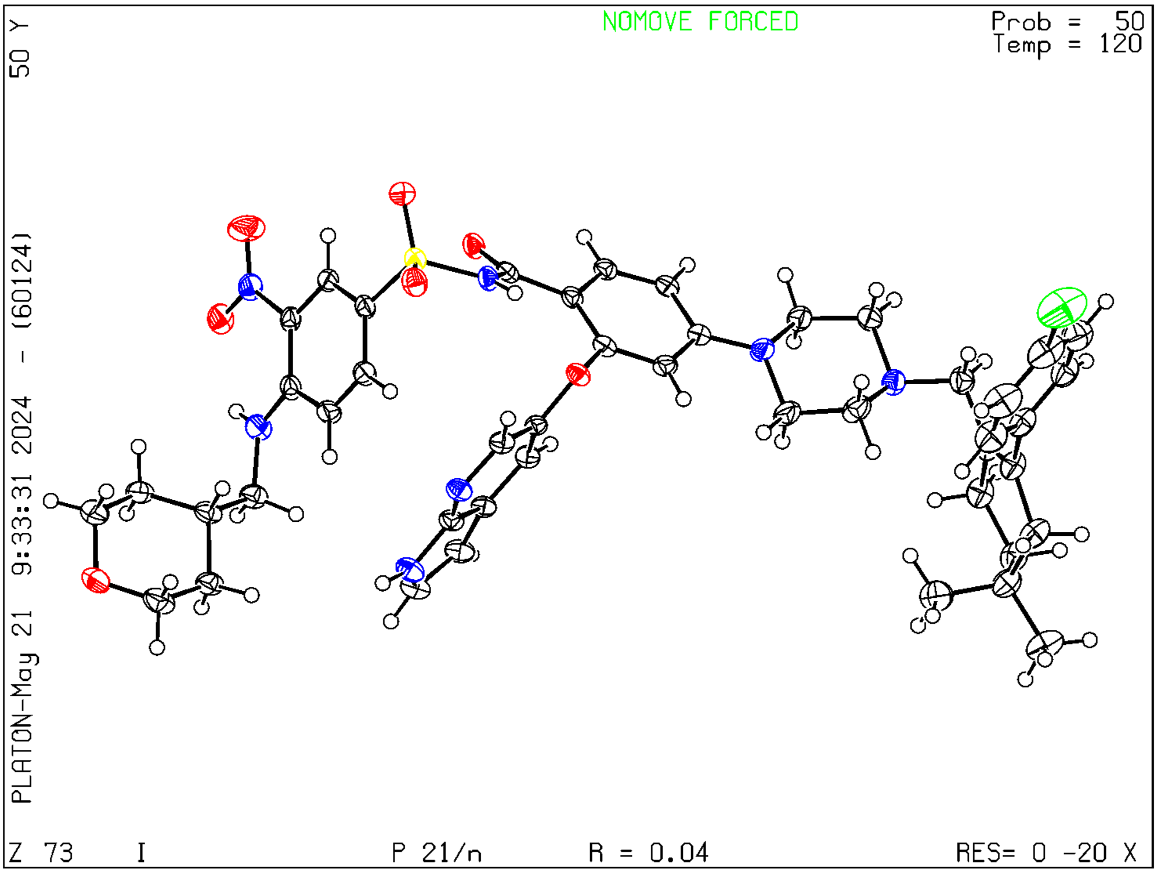

Supplement: Supplementary file 1 [file m-12-00595-sup1.zip › str for CCDC/ven EtAc final/checkcif.pdf]
