# Supplementary material for: Unravelling venetoclax solvate behaviour: insights from crystal structures and computational surface analysis
Source: IUCrJ. 2025 Aug 28;12(Pt 5):595–609. doi: 10.1107/S2052252525006785 (PMC12403167; doi:10.1107/S2052252525006785)

checkCIF/PLATON report

Structure factors have been supplied for datablock(s) I

THIS REPORT IS FOR GUIDANCE ONLY. IF USED AS PART OF A REVIEW PROCEDURE FOR PUBLICATION, IT SHOULD NOT REPLACE THE EXPERTISE OF AN EXPERIENCED CRYSTALLOGRAPHIC REFEREE.

No syntax errors found. CIF dictionary Interpreting this report

Datablock: I

Bond precision: C-C = 0.0037 A Wavelength=1.54180

Cell:	a=13.5842(2)	b=13.6070(2)	c=14.3796(3)
	alpha=105.5089(15)	beta=109.2253(16)	gamma=92.2405(15)
Temperature:	95 K		

	Calculated	Reported
Volume	2394.59(8)	2394.59(8)
Space group	P -1	P -1
Hall group	-P 1	?
Moiety formula	C44.70 H49.10 Cl N7 O7 S, C5 H10 O2, 0.3(C H3)	C50 H60.00 Cl1 N7 O9 S1
Sum formula	C50 H60 Cl N7 O9 S	C50 H60.00 Cl1 N7 O9 S1
Mr	970.56	970.58
Dx,g cm-3	1.346	1.346
Z	2	2
Mu (mm-1)	1.645	1.645
F000	1028.0	1028.0
F000'	1032.36	
h,k,lmax	17,17,18	17,17,18
Nref	10044	9882
Tmin,Tmax	0.814,0.976	0.750,0.980
Tmin'	0.713	

Correction method= # Reported T Limits: Tmin=0.750 Tmax=0.980
AbsCorr = MULTI-SCAN

Data completeness= 0.984 Theta(max)= 76.450

R(reflections)= 0.0544(8421) wR2(reflections)= 0.1610(9882)

S = 0.865 Npar= 643

The following ALERTS were generated. Each ALERT has the format
test-name_ALERT_alert-type_alert-level.
Click on the hyperlinks for more details of the test.

Alert level C

```
PLAT041_ALERT_1_C Calc. and Reported SumFormula    Strings  Differ     Please Check
              Calc: C50 H60 Cl N7 O9 S
              Rep.: C50 H60.00 Cl1 N7 O9 S1
PLAT042_ALERT_1_C Calc. and Reported MoietyFormula Strings  Differ     Please Check
              Calc: C44.70 H49.10 Cl N7 O7 S, C5 H10 O2, 0.3(C H3)
              Rep.: C50 H60.00 Cl1 N7 O9 S1
PLAT220_ALERT_2_C NonSolvent   Resd 1  C   Ueq(max)/Ueq(min) Range        3.4 Ratio
PLAT222_ALERT_3_C NonSolvent Resd 1  H   Uiso(max)/Uiso(min) Range        4.1 Ratio
PLAT230_ALERT_2_C Hirshfeld Test Diff for    C57      --C60       .       5.7 s.u.
PLAT303_ALERT_2_C Full Occupancy Atom H541      with # Connections       1.30 Check
PLAT413_ALERT_2_C Short Inter XH3 .. XHn     H603     ..H631      .      2.13 Ang.
                                                 1-x,1-y,-z  =      2_665 Check
```

Alert level G

```
PLAT002_ALERT_2_G Number of Distance or Angle Restraints on AtSite         13 Note
PLAT230_ALERT_2_G Hirshfeld Test Diff for    C54      --C55       .      12.5 s.u.
PLAT230_ALERT_2_G Hirshfeld Test Diff for    C55      --C57       .       7.0 s.u.
PLAT230_ALERT_2_G Hirshfeld Test Diff for    C57      --C58       .       8.3 s.u.
PLAT230_ALERT_2_G Hirshfeld Test Diff for    C57      --C56       .       6.2 s.u.
PLAT300_ALERT_4_G Atom Site Occupancy of C55        Constrained at        0.7 Check
PLAT300_ALERT_4_G Atom Site Occupancy of C58        Constrained at        0.7 Check
PLAT300_ALERT_4_G Atom Site Occupancy of C56        Constrained at        0.3 Check
PLAT300_ALERT_4_G Atom Site Occupancy of H551       Constrained at        0.7 Check
PLAT300_ALERT_4_G Atom Site Occupancy of H552       Constrained at        0.7 Check
PLAT300_ALERT_4_G Atom Site Occupancy of H581       Constrained at        0.7 Check
PLAT300_ALERT_4_G Atom Site Occupancy of H582       Constrained at        0.7 Check
PLAT300_ALERT_4_G Atom Site Occupancy of H583       Constrained at        0.7 Check
PLAT300_ALERT_4_G Atom Site Occupancy of H561       Constrained at        0.3 Check
PLAT300_ALERT_4_G Atom Site Occupancy of H562       Constrained at        0.3 Check
PLAT300_ALERT_4_G Atom Site Occupancy of C61        Constrained at        0.3 Check
PLAT300_ALERT_4_G Atom Site Occupancy of H611       Constrained at        0.3 Check
PLAT300_ALERT_4_G Atom Site Occupancy of H612       Constrained at        0.3 Check
PLAT300_ALERT_4_G Atom Site Occupancy of H613       Constrained at        0.3 Check
PLAT301_ALERT_3_G Main Residue  Disorder .............(Resd    1)         3% Note
PLAT302_ALERT_4_G Anion/Solvent/Minor-Residue Disorder (Resd    3)       100% Note
PLAT304_ALERT_4_G Non-Integer Number of Atoms in ..... (Resd    1)     109.80 Check
PLAT304_ALERT_4_G Non-Integer Number of Atoms in ..... (Resd    3)       1.20 Check
PLAT367_ALERT_2_G Long?  C(sp?)-C(sp?) Bond  C57      - C59       .      1.51 Ang.
PLAT412_ALERT_2_G Short Intra XH3 .. XHn     H541     ..H582      .      2.10 Ang.
                                                      x,y,z  =      1_555 Check
PLAT413_ALERT_2_G Short Inter XH3 .. XHn     H591     ..H613      .      1.98 Ang.
                                                      x,y,z  =      1_555 Check
PLAT413_ALERT_2_G Short Inter XH3 .. XHn     H603     ..H613      .      1.66 Ang.
                                                      x,y,z  =      1_555 Check
PLAT432_ALERT_2_G Short Inter X...Y Contact  C6       ..C61       .      3.07 Ang.
                                                      x,y,z  =      1_555 Check
PLAT432_ALERT_2_G Short Inter X...Y Contact  C7       ..C61       .      3.18 Ang.
                                                      x,y,z  =      1_555 Check
PLAT432_ALERT_2_G Short Inter X...Y Contact  C54      ..C61       .      2.68 Ang.
```

```
                                                      x,y,z  =      1_555 Check
PLAT432_ALERT_2_G Short Inter X...Y Contact  C57      ..C61      .       1.80 Ang.
                                                      x,y,z  =      1_555 Check
PLAT432_ALERT_2_G Short Inter X...Y Contact  C59      ..C61      .       2.38 Ang.
                                                      x,y,z  =      1_555 Check
PLAT432_ALERT_2_G Short Inter X...Y Contact  C60      ..C61      .       2.31 Ang.
                                                      x,y,z  =      1_555 Check
PLAT432_ALERT_2_G Short Inter X...Y Contact  C56      ..C61      .       2.47 Ang.
                                                      x,y,z  =      1_555 Check
PLAT432_ALERT_2_G Short Inter X...Y Contact  C56      ..C56      .       3.05 Ang.
                                                  -x,1-y,-z  =      2_565 Check
PLAT769_ALERT_4_G CIF Embedded Explicitly Supplied Scattering Data     Please Note
PLAT773_ALERT_2_G Check long C-C Bond in CIF: C57      --C61             1.80 Ang.
PLAT860_ALERT_3_G Number of Least-Squares Restraints .............         12 Note
PLAT912_ALERT_4_G Missing # of FCF Reflections Above STh/L=  0.600        161 Note
PLAT960_ALERT_3_G Number of Intensities with I  <   - 2*sig(I) ...          4 Check
PLAT969_ALERT_5_G The 'Henn et al.' R-Factor-gap value ..........       5.35 Note
              Predicted wR2: Based on SigI**2  3.01 or SHELX Weight 19.25
```

```
  0 ALERT level A = Most likely a serious problem - resolve or explain
  0 ALERT level B = A potentially serious problem, consider carefully
  7 ALERT level C = Check. Ensure it is not caused by an omission or oversight
 41 ALERT level G = General information/check it is not something unexpected

  2 ALERT type 1 CIF construction/syntax error, inconsistent or missing data
 22 ALERT type 2 Indicator that the structure model may be wrong or deficient
  4 ALERT type 3 Indicator that the structure quality may be low
 19 ALERT type 4 Improvement, methodology, query or suggestion
  1 ALERT type 5 Informative message, check
```

It is advisable to attempt to resolve as many as possible of the alerts in all categories. Often the minor alerts point to easily fixed oversights, errors and omissions in your CIF or refinement strategy, so attention to these fine details can be worthwhile. In order to resolve some of the more serious problems it may be necessary to carry out additional measurements or structure refinements. However, the purpose of your study may justify the reported deviations and the more serious of these should normally be commented upon in the discussion or experimental section of a paper or in the "special_details" fields of the CIF. checkCIF was carefully designed to identify outliers and unusual parameters, but every test has its limitations and alerts that are not important in a particular case may appear. Conversely, the absence of alerts does not guarantee there are no aspects of the results needing attention. It is up to the individual to critically assess their own results and, if necessary, seek expert advice.

Publication of your CIF in IUCr journals

A basic structural check has been run on your CIF. These basic checks will be run on all CIFs submitted for publication in IUCr journals (*Acta Crystallographica*, *Journal of Applied Crystallography*, *Journal of Synchrotron Radiation*); however, if you intend to submit to *Acta Crystallographica Section C* or *E* or *IUCrData*, you should make sure that full publication checks are run on the final version of your CIF prior to submission.

Publication of your CIF in other journals

Please refer to the *Notes for Authors* of the relevant journal for any special instructions relating to CIF submission.

PLATON version of 06/01/2024; check.def file version of 05/01/2024

Datablock I - ellipsoid plot

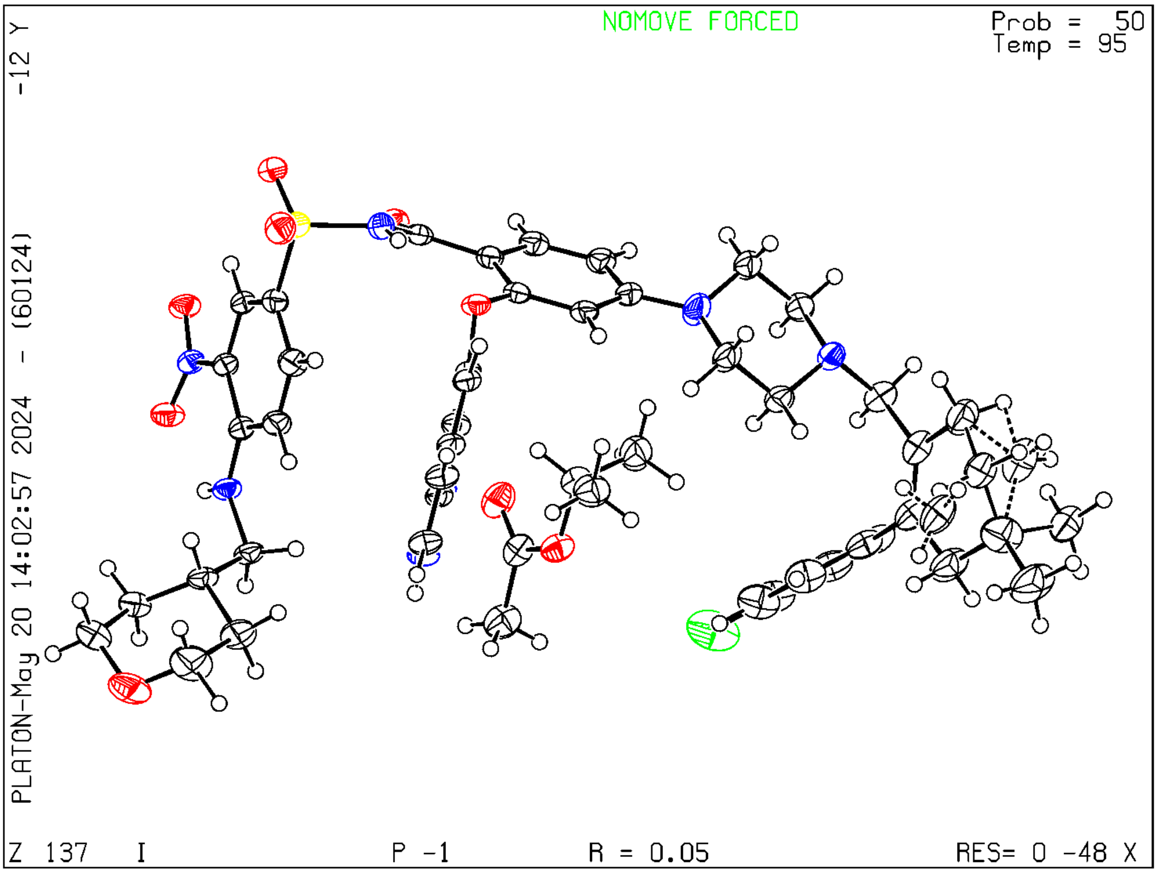

Supplement: Supplementary file 1 [file m-12-00595-sup1.zip › str for CCDC/ven iPrAc final/checkcif (1).pdf]
