# Supplementary material for: Unravelling venetoclax solvate behaviour: insights from crystal structures and computational surface analysis
Source: IUCrJ. 2025 Aug 28;12(Pt 5):595–609. doi: 10.1107/S2052252525006785 (PMC12403167; doi:10.1107/S2052252525006785)

checkCIF/PLATON report

Structure factors have been supplied for datablock(s) I

THIS REPORT IS FOR GUIDANCE ONLY. IF USED AS PART OF A REVIEW PROCEDURE FOR PUBLICATION, IT SHOULD NOT REPLACE THE EXPERTISE OF AN EXPERIENCED CRYSTALLOGRAPHIC REFEREE.

No syntax errors found. CIF dictionary Interpreting this report

Datablock: I

Bond precision:	C-C = 0.0030 A	Wavelength=1.54180	
Cell:	a=14.2655(1)	b=12.2567(1)	c=30.0079(2)
	alpha=90	beta=92.7510(4)	gamma=90
Temperature:	95 K		

	Calculated	Reported
Volume	5240.77(7)	5240.77(7)
Space group	P 21/n	P 21/n
Hall group	-P 2yn	?
Moiety formula	C45 H50 Cl N7 O7 S, 2(C4 H8 O)	C53 H66 Cl1 N7 O9 S1
Sum formula	C53 H66 Cl N7 O9 S	C53 H66 Cl1 N7 O9 S1
Mr	1012.64	1012.67
Dx,g cm-3	1.283	1.283
Z	4	4
Mu (mm-1)	1.524	1.524
F000	2152.0	2152.0
F000'	2160.93	
h,k,lmax	17,15,37	17,15,37
Nref	10594	10519
Tmin,Tmax	0.813,0.868	0.660,0.870
Tmin'	0.479	

Correction method= # Reported T Limits: Tmin=0.660 Tmax=0.870
AbsCorr = MULTI-SCAN

Data completeness= 0.993 Theta(max)= 73.644

R(reflections)= 0.0534(9895) wR2(reflections)= 0.1566(10519)

S = 0.997 Npar= 668

The following ALERTS were generated. Each ALERT has the format
test-name_ALERT_alert-type_alert-level.
Click on the hyperlinks for more details of the test.

Alert level C

```
DIFMX02_ALERT_1_C  The maximum difference density is > 0.1*ZMAX*0.75
            The relevant atom site should be identified.
PLAT041_ALERT_1_C Calc. and Reported SumFormula    Strings  Differ     Please Check
              Calc: C53 H66 Cl N7 O9 S
              Rep.: C53 H66 Cl1 N7 O9 S1
PLAT042_ALERT_1_C Calc. and Reported MoietyFormula Strings  Differ     Please Check
              Calc: C45 H50 Cl N7 O7 S, 2(C4 H8 O)
              Rep.: C53 H66 Cl1 N7 O9 S1
PLAT097_ALERT_2_C Large Reported Max.  (Positive) Residual Density       1.54 eA-3
PLAT260_ALERT_2_C Large Average Ueq of Residue Including        O3      0.188 Check
PLAT309_ALERT_2_C Single Bonded Oxygen (C-O > 1.3 Ang) ..........          O3 Check
PLAT362_ALERT_2_C Short  C(sp3)-C(sp2) Bond  C9       - C63      .       1.36 Ang.
PLAT410_ALERT_2_C Short Intra H...H Contact  H141     ..H521     .       1.92 Ang.
                                                       x,y,z  =      1_555 Check
PLAT737_ALERT_1_C D...A   Calc  2.9105(19), Rep    2.911(4) ......         2.1 s.u.-R
              N40      -O28             1_555   2_556 ........     #    2 Check
PLAT737_ALERT_1_C D...A   Calc  2.7673(19), Rep    2.767(4) ......         2.1 s.u.-R
              N29      -O16             1_555   1_555 ........     #    4 Check
PLAT737_ALERT_1_C D...A   Calc  2.9461(19), Rep    2.946(4) ......         2.1 s.u.-R
              N29      -O45             1_555   1_565 ........     #    5 Check
```

Alert level G

```
PLAT002_ALERT_2_G Number of Distance or Angle Restraints on AtSite         13 Note
PLAT007_ALERT_5_G Number of Unrefined Donor-H Atoms .............           3 Report
              H211  H291  H401
PLAT083_ALERT_2_G SHELXL Second Parameter in WGHT  Unusually Large       5.74 Why ?
PLAT142_ALERT_4_G s.u. on b - Axis Small or Missing .............    0.00010 Ang.
PLAT143_ALERT_4_G s.u. on c - Axis Small or Missing .............    0.00020 Ang.
PLAT145_ALERT_4_G s.u. on beta     Small or Missing .............     0.0004 Degree
PLAT175_ALERT_4_G The CIF-Embedded .res File Contains SAME Records          2 Report
PLAT177_ALERT_4_G The CIF-Embedded .res File Contains DELU Records          2 Report
PLAT178_ALERT_4_G The CIF-Embedded .res File Contains SIMU Records          2 Report
PLAT188_ALERT_3_G A Non-default SIMU Restraint Value has been used     0.0100 Report
PLAT188_ALERT_3_G A Non-default SIMU Restraint Value has been used     0.0100 Report
PLAT302_ALERT_4_G Anion/Solvent/Minor-Residue Disorder (Resd    2)        60% Note
PLAT769_ALERT_4_G CIF Embedded Explicitly Supplied Scattering Data     Please Note
PLAT860_ALERT_3_G Number of Least-Squares Restraints ............        1128 Note
PLAT912_ALERT_4_G Missing # of FCF Reflections Above STh/L=  0.600         75 Note
PLAT960_ALERT_3_G Number of Intensities with I  <   - 2*sig(I) ...          3 Check
PLAT969_ALERT_5_G The 'Henn et al.' R-Factor-gap value ..........        7.29 Note
              Predicted wR2: Based on SigI**2  2.15 or SHELX Weight 16.23
```

0 **ALERT level A** = Most likely a serious problem - resolve or explain
0 **ALERT level B** = A potentially serious problem, consider carefully
11 **ALERT level C** = Check. Ensure it is not caused by an omission or oversight
17 **ALERT level G** = General information/check it is not something unexpected

6 ALERT type 1 CIF construction/syntax error, inconsistent or missing data

```
7 ALERT type 2 Indicator that the structure model may be wrong or deficient
4 ALERT type 3 Indicator that the structure quality may be low
9 ALERT type 4 Improvement, methodology, query or suggestion
2 ALERT type 5 Informative message, check
```

It is advisable to attempt to resolve as many as possible of the alerts in all categories. Often the minor alerts point to easily fixed oversights, errors and omissions in your CIF or refinement strategy, so attention to these fine details can be worthwhile. In order to resolve some of the more serious problems it may be necessary to carry out additional measurements or structure refinements. However, the purpose of your study may justify the reported deviations and the more serious of these should normally be commented upon in the discussion or experimental section of a paper or in the "special_details" fields of the CIF. checkCIF was carefully designed to identify outliers and unusual parameters, but every test has its limitations and alerts that are not important in a particular case may appear. Conversely, the absence of alerts does not guarantee there are no aspects of the results needing attention. It is up to the individual to critically assess their own results and, if necessary, seek expert advice.

Publication of your CIF in IUCr journals

A basic structural check has been run on your CIF. These basic checks will be run on all CIFs submitted for publication in IUCr journals (*Acta Crystallographica*, *Journal of Applied Crystallography*, *Journal of Synchrotron Radiation*); however, if you intend to submit to *Acta Crystallographica Section C* or *E* or *IUCrData*, you should make sure that full publication checks are run on the final version of your CIF prior to submission.

Publication of your CIF in other journals

Please refer to the *Notes for Authors* of the relevant journal for any special instructions relating to CIF submission.

PLATON version of 06/01/2024; check.def file version of 05/01/2024

Datablock I - ellipsoid plot

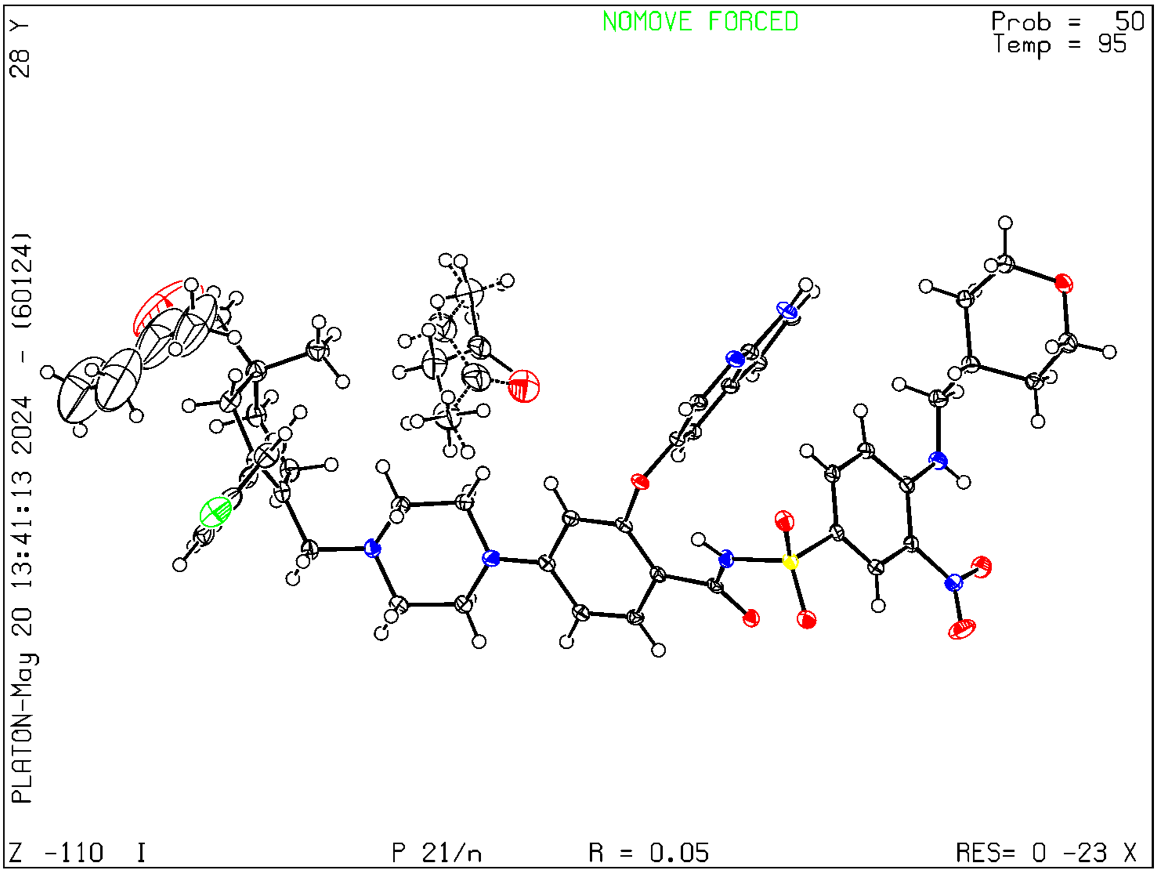

Supplement: Supplementary file 1 [file m-12-00595-sup1.zip › str for CCDC/ven MEK final/checkcif.pdf]
