# Supplementary material for: Unravelling venetoclax solvate behaviour: insights from crystal structures and computational surface analysis
Source: IUCrJ. 2025 Aug 28;12(Pt 5):595–609. doi: 10.1107/S2052252525006785 (PMC12403167; doi:10.1107/S2052252525006785)
Supplement: Supplementary file 2 [file m-12-00595-sup2.pdf]

IUCrJ

Volume 12 (2025)

Supporting information for article:

Unravelling venetoclax solvate behaviour: insights from crystal structures and computational surface analysis

Eliška Zmeškalová, Tereza Havlůjová, Martin Babor, Marcela Tkadlecová, Jaroslav Havlíček, Tomáš Pekárek, Szymon Tomczak, Luděk Ridvan and Miroslav Šoóš

Supporting Information

Unravelling Venetoclax Solvate Behaviour: Insights from Crystal Structures and Computational Surface Analysis

Eliška Zmeškalová*[1,2], Tereza Havlůjová*[1,3], Martin Babor[4], Marcela Tkadlecová[3], Jaroslav Havlíček[3], Tomáš Pekárek[3], Szymon Tomczak[5], Luděk Ridvan[3], Miroslav Šoóš[1]

Table S1: Crystallographic details

**Crystal data**	**ven_AN2**	**ven_MEK**	**ven_mTHF**	**ven_EA**	**ven_IPAC**	**ven_DIO**	**ven_AE**
Chemical formula	$C_{45}H_{50}ClN_7O_7S·C_2H_3N$	$C_{53}H_{66}ClN_7O_9S$	$C_{55}H_{69}ClN_7O_9S$	$C_{45}H_{50}ClN_7O_7S$	$C_{50}H_{60}ClN_7O_9S$	$C_{61}H_{82}ClN_7O_{15}S$	$C_{48}H_{56}ClN_7O_8S$
M_r	909.51	1012.67	1039.71	868.45	970.58	1220.88	926.53
Crystal system, space group	Triclinic, *P*-1	Monoclinic, $P2_1/n$	Monoclinic, $P2_1/n$	Monoclinic, $P2_1/n$	Triclinic, *P*-1	Triclinic, *P*-1	Triclinic, *P*-1
Temperature (K)	95	95	120	120	95	95	95
a, *b*, *c* (Å)	12.4625 (1), 13.1131 (2), 15.3650 (2)	14.2655 (1), 12.2567 (1), 30.0079 (2)	14.6928 (1), 12.3178 (1), 29.8810 (2)	13.7621 (1), 12.5121 (1), 29.9705 (3)	13.5842 (2), 13.6070 (2), 14.3796 (3)	10.6785 (4), 13.5301 (5), 21.9604 (8)	12.0250 (5), 13.6215 (6), 15.7475 (8)
α, β, γ (°)	93.2334 (11), 96.3237 (10), 113.9359 (12)	90, 92.7510 (4), 90	90, 93.2176 (6), 90	90, 92.5017 (8), 90	105.5089 (15), 109.2253 (16), 92.2405 (15)	82.660 (3), 88.791 (3), 80.515 (3)	93.425 (4), 110.937 (5), 102.318 (4)
V (Å^3)	2266.90 (5)	5240.77 (7)	5399.43 (7)	5155.79 (8)	2394.59 (8)	3103.8 (2)	2327.4 (2)
Z	2	4	4	4	2	2	2
Radiation type	Cu Kα	Cu *K*α	Cu *K*α	Cu *K*α	Cu *K*α	Cu *K*α	Cu *K*α
μ (mm^{-1})	1.68	1.52	1.49	1.45	1.65	1.45	1.65
Crystal size (mm)	0.19 × 0.12 × 0.07	0.48 × 0.11 × 0.09	0.26 × 0.19 × 0.13	0.44 × 0.26 × 0.09	0.21 × 0.10 × 0.02	0.12 × 0.07 × 0.02	0.13 × 0.08 × 0.02
Diffractometer	Oxford Diffraction SuperNova	Oxford Diffraction SuperNova	Oxford Diffraction SuperNova	Oxford Diffraction SuperNova	Oxford Diffraction SuperNova	Oxford Diffraction SuperNova	Oxford Diffraction SuperNova
Absorption correction	Multi-scan CrysAlis PRO, (Agilent, 2011)	Multi-scan *CrysAlis PRO* (Rigaku Oxford Diffraction, 2017)	Multi-scan *CrysAlis PRO* (Rigaku Oxford Diffraction, 2017)	Multi-scan *CrysAlis PRO* (Rigaku Oxford Diffraction, 2017)	Multi-scan *CrysAlis PRO* (Rigaku Oxford Diffraction, 2017)	Multi-scan *CrysAlis PRO* (Rigaku Oxford Diffraction, 2017)	Multi-scan *CrysAlis PRO* (Rigaku Oxford Diffraction, 2017)
T_{min}, T_{max}	0.40, 0.89	0.66, 0.87	0.79, 0.82	0.65, 0.88	0.75, 0.98	0.86, 0.97	0.65, 0.96
No. of measured, indep. and observed [$I > 2.0σ(I)$] reflections	80456, 8998, 8362	78127, 10519, 9895	101939, 9722, 8904	90391, 9247, 8031	41714, 9882, 8421	27655, 12204, 9486	31486, 8525, 5262
R_{int}	0.034	0.047	0.030	0.037	0.028	0.052	0.198
$(\sin θ/λ)_{max}$ (Å^{-1})	0.622	0.622	0.600	0.599	0.631	0.625	0.622
$R[F^2 > 2σ(F^2)]$, $wR(F^2)$, S	0.040, 0.103, 1.00	0.053, 0.157, 1.00	0.067, 0.197, 0.98	0.040, 0.110, 0.96	0.054, 0.161, 0.87	0.057, 0.173, 0.99	0.056, 0.148, 0.89

No. of reflections	8998	10519	9722	9246	9882	12204	8523
No. of parameters	788	668	677	550	643	815	586
No. of restraints	374	1128	148	760	12	120	0
H-atom treatment	H atoms treated by a mixture of independent and constrained refinement	H-atom parameters constrained	H-atom parameters constrained	H-atom parameters constrained	H atoms treated by a mixture of independent and constrained refinement	H atoms treated by a mixture of independent and constrained refinement	H-atom parameters not refined
$\Delta\rho_{max}$, $\Delta\rho_{min}$ (e Å^{-3})	0.40, −0.61	1.54, −1.16	1.31, −0.72	0.29, −0.43	1.04, −1.26	0.81, −1.28	0.60, −0.61

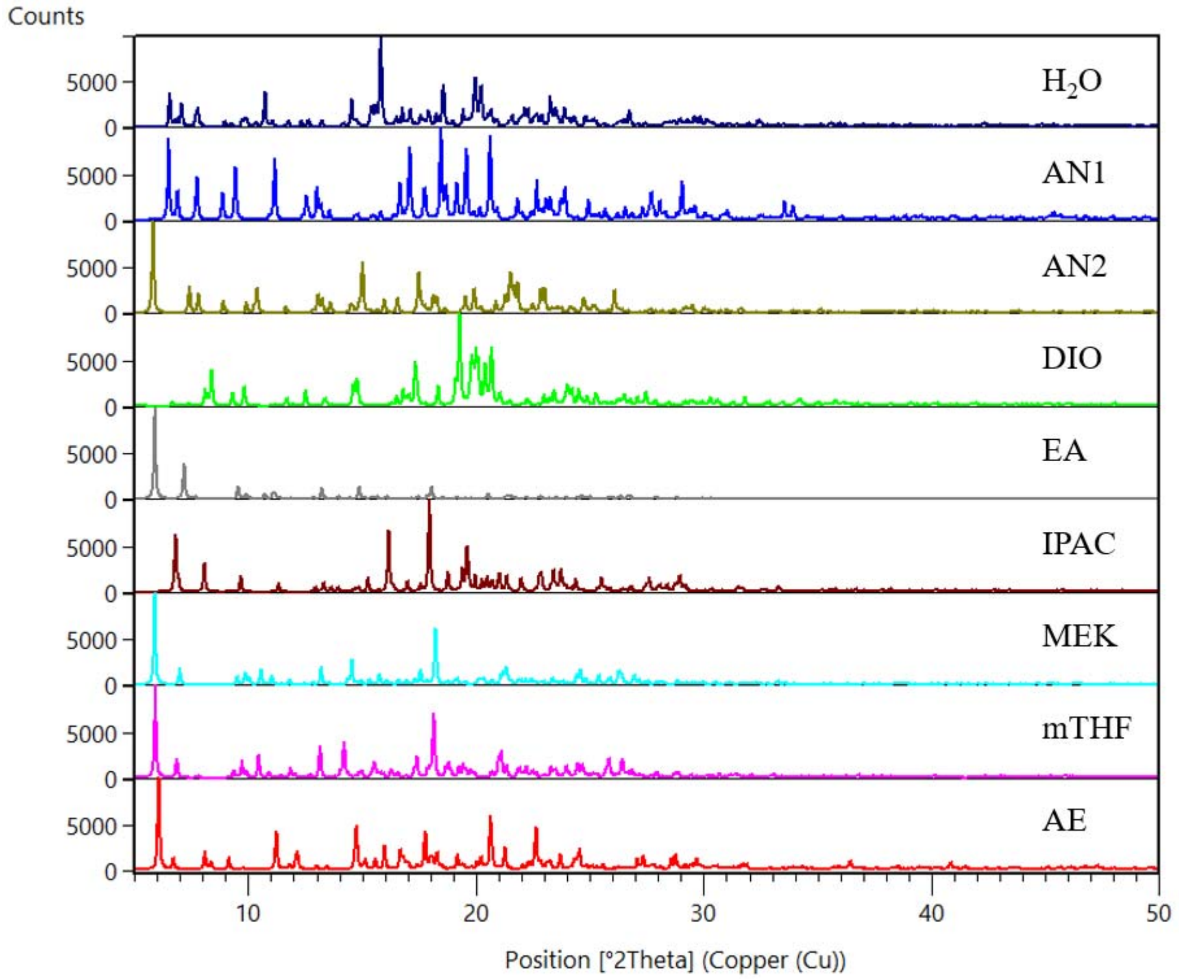

Fig. S1 XRPD patterns calculated from the crystal structures of venetoclax solvates

	2143_publish.cif 0	ven hyd AQUPEK.cif 1	ven_ACN1_publish.cif 2	ven_diox_publish.cif 3	ven_EtAc_publish.cif 4	ven_iPrAc_publish.cif 5	ven_MEK_publish.cif 6	ven_MHF_publish.cif 7
0: 2143_publish.cif	0.0000	137.8535	208.3394	234.7497	251.0175	228.9593	248.6554	244.3040
1: ven hyd AQUPEK.cif	137.8535	0.0000	218.6298	193.0204	176.0397	195.4475	179.1322	176.9382
2: ven_ACN1_publish.cif	208.3394	218.6298	0.0000	239.7048	140.8303	172.8550	139.8780	139.3494
3: ven_diox_publish.cif	234.7497	193.0204	239.7048	0.0000	230.5089	205.6107	226.4380	222.4121
4: ven_EtAc_publish.cif	251.0175	176.0397	140.8303	230.5089	0.0000	176.5633	0.6030	1.8436
5: ven_iPrAc_publish.cif	228.9593	195.4475	172.8550	205.6107	176.5633	0.0000	164.1450	167.6805
6: ven_MEK_publish.cif	248.6554	179.1322	139.8780	226.4380	0.6030	164.1450	0.0000	0.6868
7: ven_MHF_publish.cif	244.3040	176.9382	139.3494	222.4121	1.8436	167.6805	0.6868	0.0000

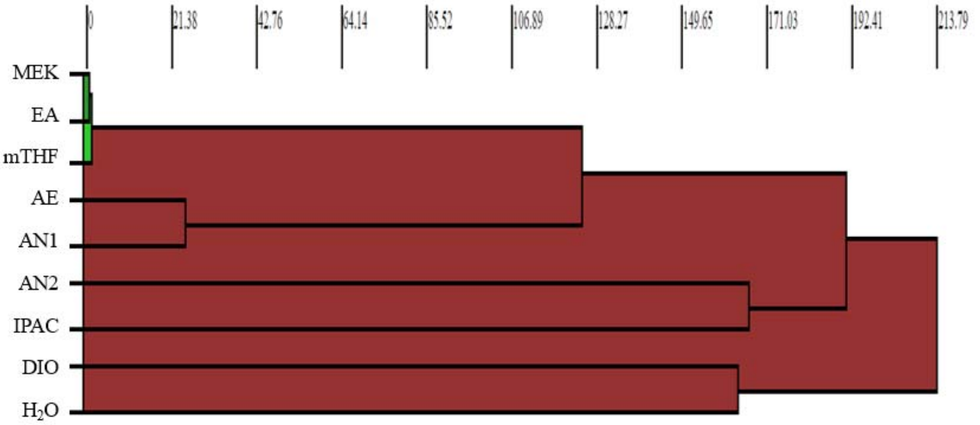

Fig. S2: Similarity matrix and tree diagram of venetoclax molecular packing in all available structures. Calculated by CrystalCMP.

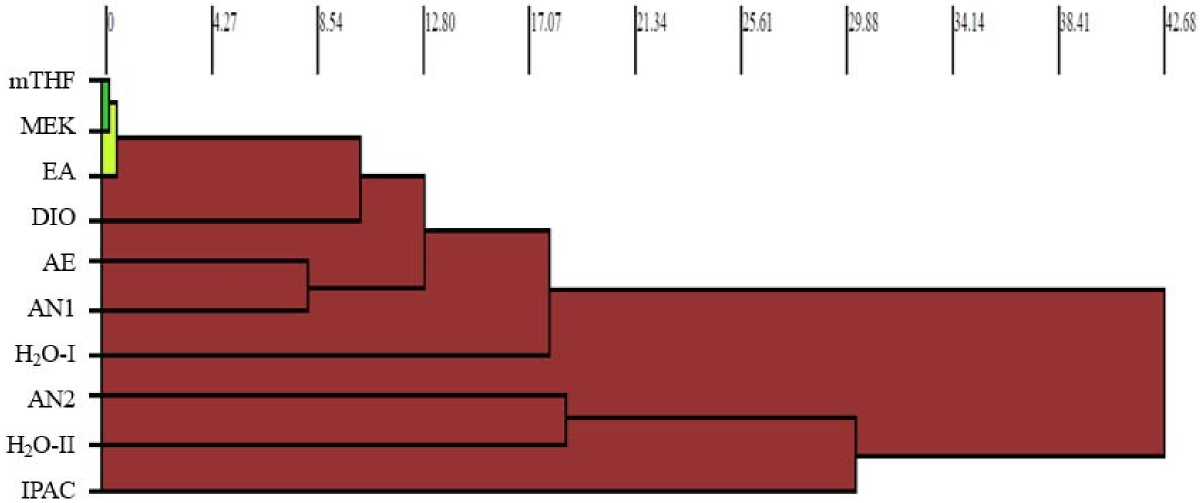

Fig. S3: Similarity tree diagram of venetoclax conformations in all available structures. Calculated by CrystalCMP.

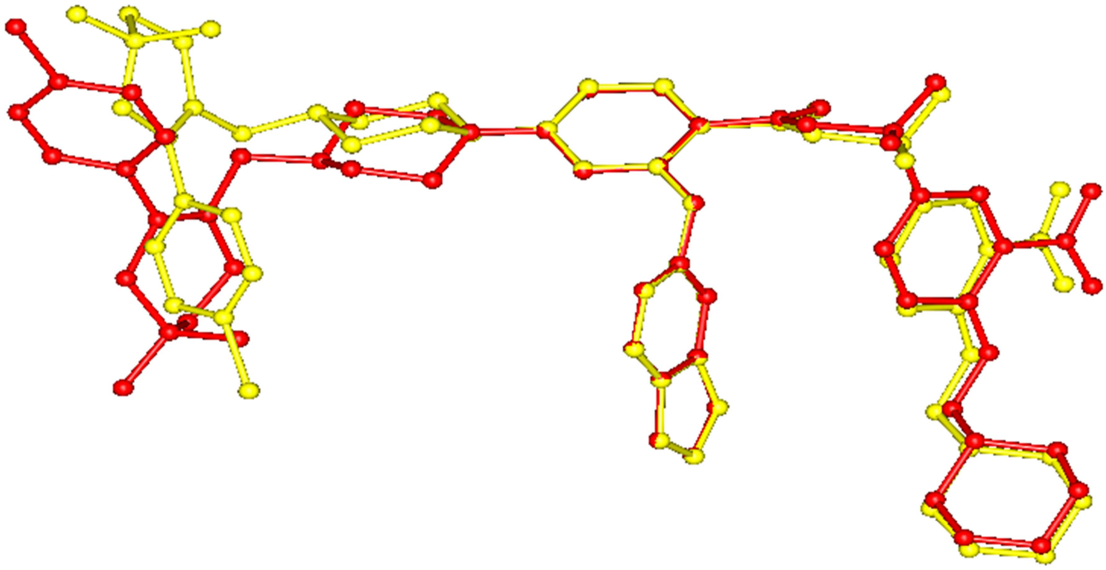

Fig. S4: Conformation comparison between VEN AE and VEN AN1

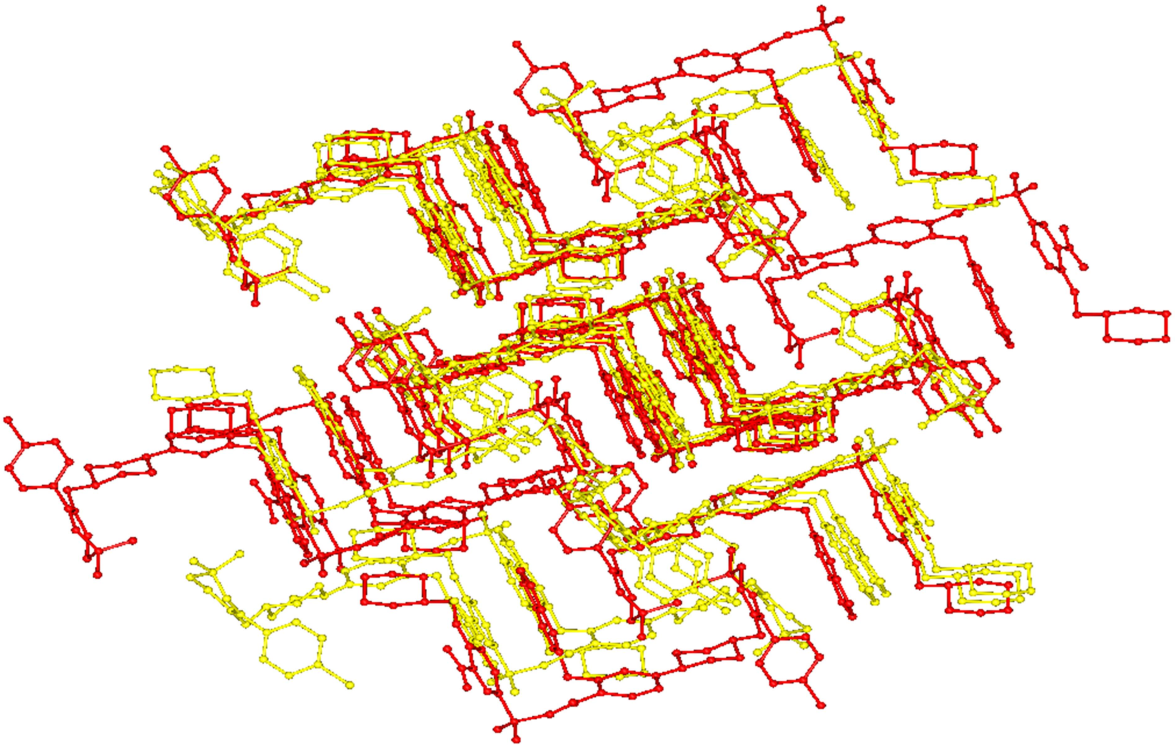

Fig. S5: Packing comparison between VEN AE and VEN AN1

<u>NMR spectra transcripts - Desolvated samples</u>

Acetone solvate – dried

^{1}H NMR (500 MHz, DMSO) δ 11.69 (s, 1H), 11.42 (s, 1H), 8.60 (d, *J* = 6.4 Hz, 1H), 8.56 (d, *J* = 2.3 Hz, 1H), 8.04 (d, *J* = 2.6 Hz, 1H), 7.83 – 7.77 (m, 1H), 7.56 – 7.46 (m, 3H), 7.37 – 7.31 (m, 2H), 7.11 (d, *J* = 9.3 Hz, 1H), 7.06 – 7.01 (m, 2H), 6.68 (dd, *J* = 9.2, 2.3 Hz, 1H), 6.39 (dd, *J* = 3.4, 1.9 Hz, 1H), 6.19 (d, *J* = 2.3 Hz, 1H), 3.88 – 3.81 (m, 2H), 3.30 (d, *J* = 6.7 Hz, 2H), 3.30 – 3.22 (m, 2H), 3.07 (s, 4H), 2.75 (s, 2H), 2.19 (s, 4H), 2.14 (s, 2H), 2.08 (s, 4H), 1.95 (s, 2H), 1.89 (ddd, *J* = 11.1, 7.4, 3.9 Hz, 1H), 1.61 (d, *J* = 12.7 Hz, 2H), 1.38 (t, *J* = 6.6 Hz, 2H), 1.25 (qd, *J* = 11.9, 4.5 Hz, 2H), 0.92 (s, 5H).

Acetonitrile solvate – dried

^{1}H NMR (500 MHz, DMSO) δ 11.68 (d, *J* = 2.5 Hz, 1H), 11.38 (s, 1H), 8.61 (t, *J* = 6.0 Hz, 1H), 8.56 (d, *J* = 2.3 Hz, 1H), 8.04 (d, *J* = 2.6 Hz, 1H), 7.80 (dd, *J* = 9.3, 2.3 Hz, 1H), 7.54 (d, *J* = 2.6

Hz, 1H), 7.52 – 7.46 (m, 2H), 7.37 – 7.31 (m, 2H), 7.11 (d, *J* = 9.3 Hz, 1H), 7.07 – 7.01 (m, 2H), 6.68 (dd, *J* = 9.0, 2.4 Hz, 1H), 6.39 (dd, *J* = 3.4, 1.9 Hz, 1H), 6.19 (d, *J* = 2.3 Hz, 1H), 3.85 (ddd, *J* = 11.4, 4.5, 1.9 Hz, 2H), 3.32 – 3.22 (m, 4H), 3.07 (s, 4H), 2.76 (s, 2H), 2.20 (s, 4H), 2.14 (t, *J* = 6.5 Hz, 2H), 1.95 (s, 2H), 1.89 (ddq, *J* = 11.4, 7.8, 3.8 Hz, 1H), 1.64 – 1.58 (m, 2H), 1.38 (t, *J* = 6.5 Hz, 2H), 1.25 (qd, *J* = 11.9, 4.5 Hz, 2H), 0.92 (s, 5H).

Dioxane solvate – dried

^{1}H NMR (500 MHz, DMSO) δ 11.69 (s, 1H), 11.46 (s, 1H), 8.61 (s, 1H), 8.56 (s, 1H), 8.04 (d, *J* = 2.6 Hz, 1H), 7.80 (d, *J* = 9.0 Hz, 1H), 7.54 (s, 1H), 7.52 – 7.46 (m, 2H), 7.37 – 7.32 (m, 2H), 7.11 (d, *J* = 9.3 Hz, 1H), 7.07 – 7.01 (m, 2H), 6.68 (dd, *J* = 9.3, 2.3 Hz, 1H), 6.39 (dd, *J* = 3.4, 1.9 Hz, 1H), 6.19 (d, *J* = 2.3 Hz, 1H), 3.85 (dd, *J* = 11.4, 4.2 Hz, 2H), 3.57 (s, 2H), 3.26 (dd, *J* = 13.2, 11.2 Hz, 3H), 3.07 (s, 4H), 2.74 (s, 2H), 2.18 (s, 4H), 2.14 (s, 2H), 1.95 (s, 2H), 1.88 (dd, *J* = 11.2, 7.2 Hz, 1H), 1.61 (d, *J* = 11.7 Hz, 2H), 1.38 (t, *J* = 6.5 Hz, 2H), 1.25 (qd, *J* = 12.1, 4.4 Hz, 2H), 0.92 (s, 6H).

Ethyl acetate solvate – dried

^{1}H NMR (500 MHz, DMSO) δ 11.68 (s, 1H), 11.38 (s, 1H), 8.60 (t, *J* = 6.0 Hz, 1H), 8.56 (d, *J* = 2.3 Hz, 1H), 8.04 (d, *J* = 2.6 Hz, 1H), 7.80 (dd, *J* = 9.2, 2.3 Hz, 1H), 7.55 – 7.46 (m, 3H), 7.37 – 7.31 (m, 2H), 7.11 (d, *J* = 9.3 Hz, 1H), 7.06 – 7.01 (m, 2H), 6.68 (dd, *J* = 9.0, 2.3 Hz, 1H), 6.39 (dd, *J* = 3.4, 1.9 Hz, 1H), 6.19 (d, *J* = 2.3 Hz, 1H), 3.85 (dt, *J* = 12.2, 2.7 Hz, 2H), 3.32 – 3.22 (m, 4H), 3.07 (s, 4H), 2.75 (s, 2H), 2.20 (s, 4H), 2.14 (s, 2H), 1.95 (s, 2H), 1.89 (dq, *J* = 11.0, 5.4 Hz, 1H), 1.61 (d, *J* = 12.9 Hz, 2H), 1.38 (t, *J* = 6.6 Hz, 2H), 1.25 (qd, *J* = 12.1, 4.4 Hz, 2H), 0.92 (s, 5H).

Isopropyl acetate solvate – dried

^{1}H NMR (500 MHz, DMSO) δ 11.69 (s, 1H), 11.44 (s, 1H), 8.61 (s, 1H), 8.56 (d, *J* = 2.3 Hz, 1H), 8.04 (d, *J* = 2.6 Hz, 1H), 7.80 (d, *J* = 8.8 Hz, 1H), 7.56 – 7.46 (m, 3H), 7.37 – 7.32 (m, 2H), 7.11 (d, *J* = 9.4 Hz, 1H), 7.06 – 7.01 (m, 2H), 6.68 (dd, *J* = 9.0, 2.3 Hz, 1H), 6.39 (dd, *J* = 3.4, 1.9 Hz, 1H), 6.19 (d, *J* = 2.3 Hz, 1H), 3.88 – 3.81 (m, 2H), 3.32 – 3.25 (m, 3H), 3.28 – 3.22 (m, 1H), 3.07 (s, 4H), 2.75 (s, 2H), 2.19 (s, 5H), 2.14 (s, 2H), 1.95 (s, 2H), 1.93 – 1.85 (m, 1H), 1.61 (d, *J* = 12.7 Hz, 2H), 1.38 (t, *J* = 6.5 Hz, 2H), 1.25 (qd, *J* = 12.1, 4.4 Hz, 2H), 1.20 – 1.13 (m, 1H), 0.92 (s, 5H).

Methyl ethyl ketone solvate – dried

^{1}H NMR (500 MHz, DMSO) δ 11.68 (s, 1H), 11.39 (s, 1H), 8.60 (t, *J* = 6.0 Hz, 1H), 8.56 (d, *J* = 2.3 Hz, 1H), 8.04 (d, *J* = 2.6 Hz, 1H), 7.80 (dd, *J* = 9.2, 2.3 Hz, 1H), 7.55 – 7.46 (m, 3H), 7.37 – 7.31 (m, 2H), 7.11 (d, *J* = 9.3 Hz, 1H), 7.07 – 7.01 (m, 2H), 6.68 (dd, *J* = 9.0, 2.4 Hz, 1H), 6.39 (dd, *J* = 3.4, 1.9 Hz, 1H), 6.19 (d, *J* = 2.3 Hz, 1H), 3.88 – 3.81 (m, 2H), 3.32 – 3.22 (m, 3H), 3.07 (s, 3H), 2.75 (s, 2H), 2.22 – 2.03 (m, 6H), 1.95 (s, 2H), 1.89 (ddd, *J* = 11.2, 7.3, 3.9 Hz, 1H), 1.66 – 1.58 (m, 2H), 1.38 (t, *J* = 6.6 Hz, 2H), 1.34 – 1.25 (m, 1H), 1.23 (dd, *J* = 11.9, 4.5 Hz, 1H), 0.92 (s, 5H), 0.92 – 0.76 (m, 1H).

2-methyl tetrahydrofuran – dried

^{1}H NMR (500 MHz, DMSO) δ 11.68 (s, 1H), 11.43 (s, 1H), 8.60 (s, 1H), 8.56 (d, *J* = 2.2 Hz, 1H), 8.04 (d, *J* = 2.6 Hz, 1H), 7.83 – 7.77 (m, 1H), 7.55 – 7.46 (m, 3H), 7.37 – 7.31 (m, 2H), 7.11 (d, *J* = 9.4 Hz, 1H), 7.07 – 7.01 (m, 2H), 6.68 (dd, *J* = 9.0, 2.4 Hz, 1H), 6.39 (dd, *J* = 3.4, 1.9 Hz, 1H), 6.19 (d, *J* = 2.3 Hz, 1H), 3.88 – 3.81 (m, 2H), 3.32 – 3.25 (m, 3H), 3.28 – 3.22 (m, 1H), 3.07 (s, 4H), 2.75 (s, 2H), 2.19 (s, 4H), 2.14 (s, 2H), 1.95 (s, 2H), 1.89 (ddd, *J* = 11.3, 7.5, 3.9 Hz, 1H), 1.61 (d, *J* = 12.3 Hz, 2H), 1.38 (t, *J* = 6.5 Hz, 2H), 1.25 (qd, *J* = 12.1, 4.5 Hz, 2H), 0.92 (s, 5H).

Tab. S2: Solvent content in the dried samples

Solvent	^{1}H NMR residual solvent mol. eq.
AE	0.9
AN	0.09
DIO	0.5
EA	0
IPAC	0.13
MEK	0.2
mTHF	0.06

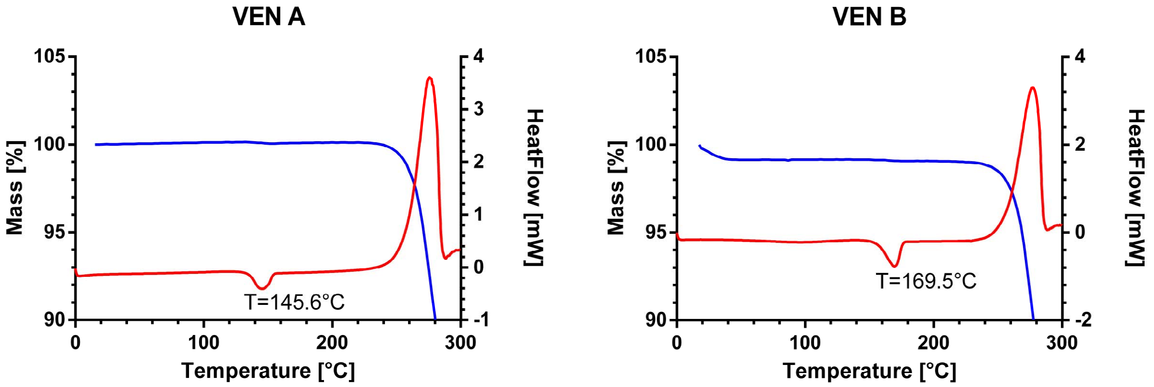

Fig. S6: TGA and DSC of venetoclax desolvated forms, VEN A, VEN B

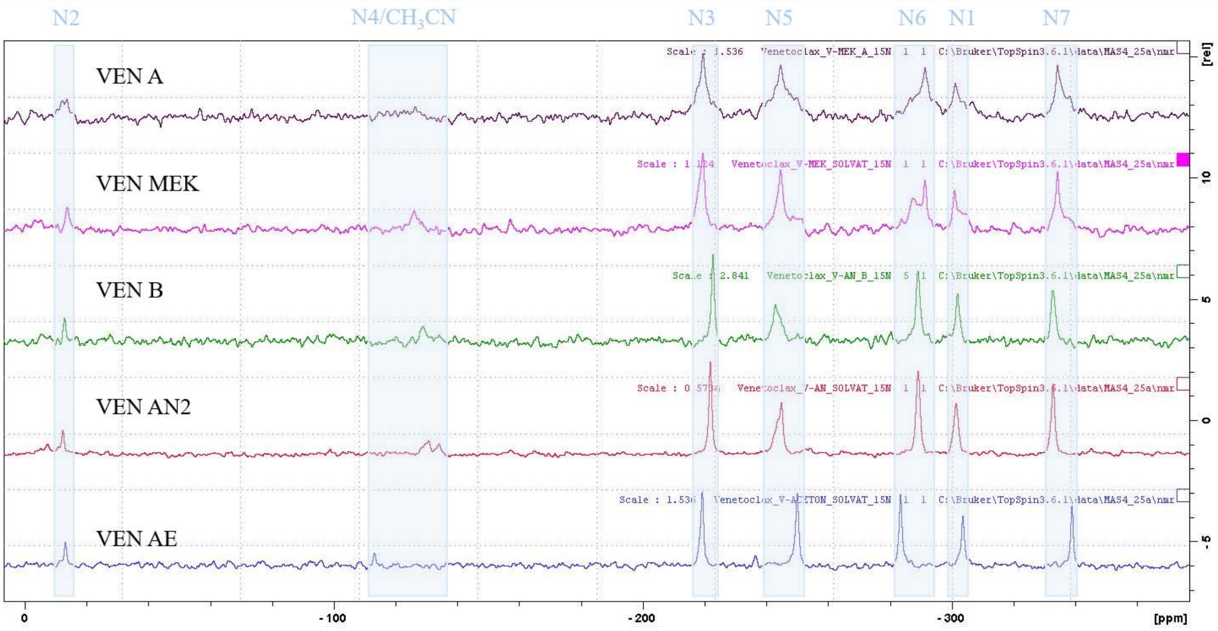

Fig. S7 ^{15}N ssNMR spectra

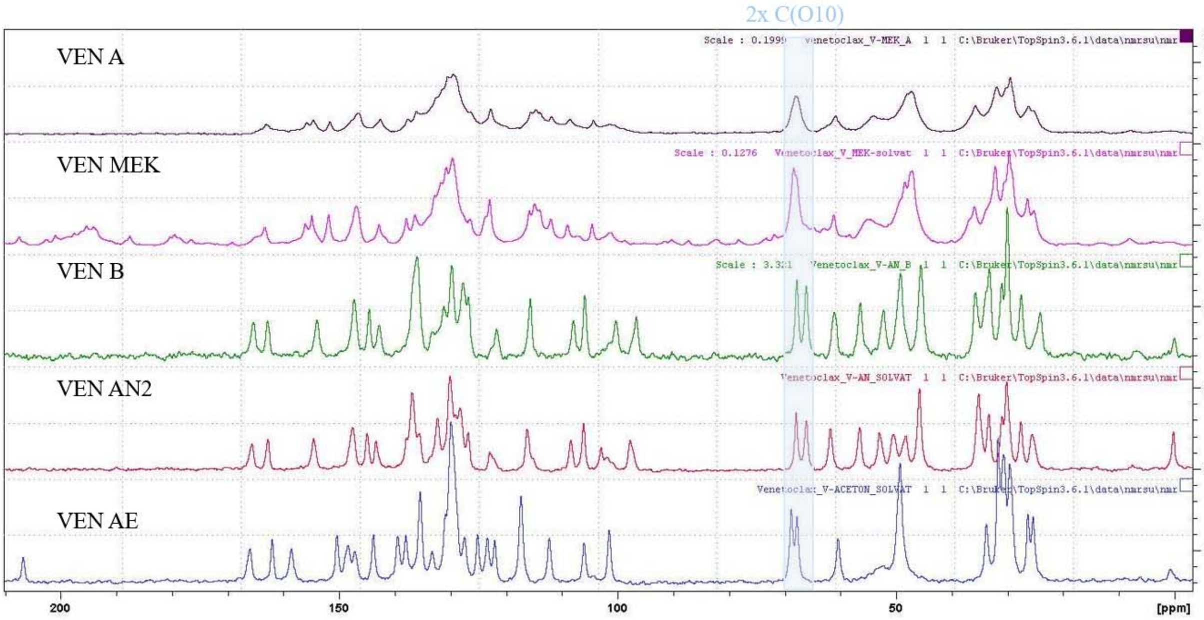

Fig. S8 ^{13}C ssNMR spectra

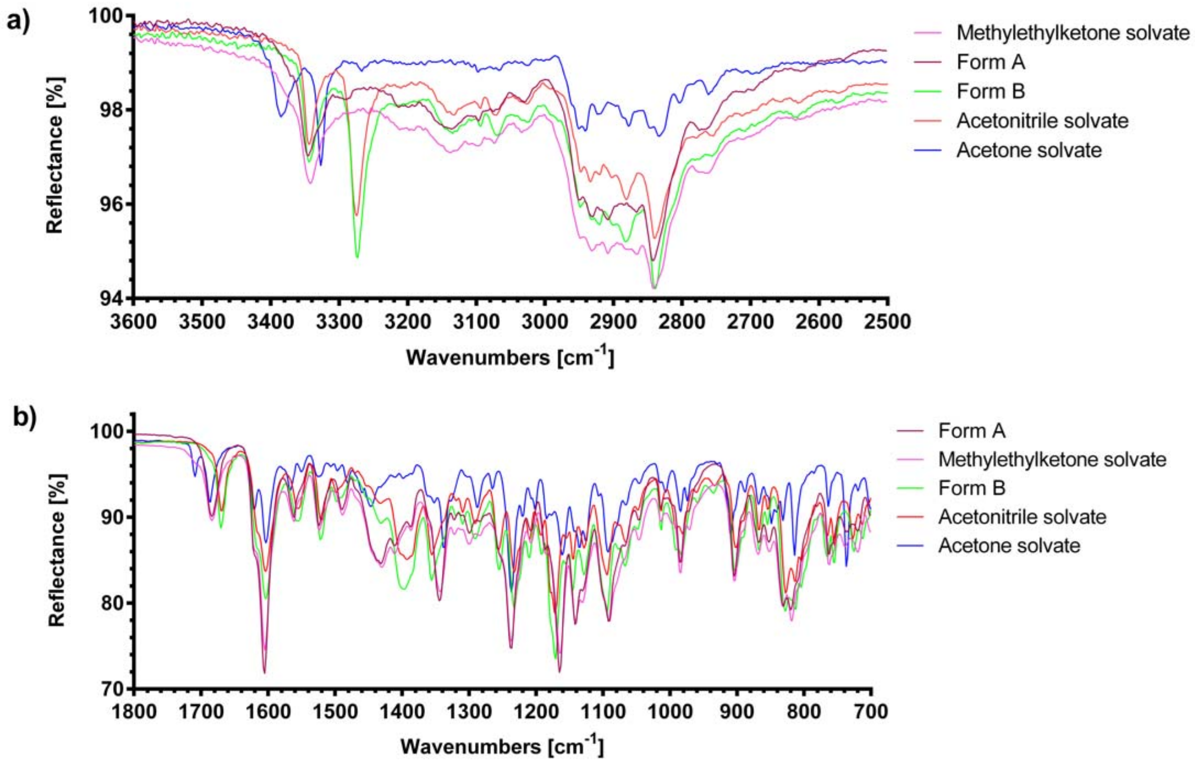

Fig. S9 FTIR spectra

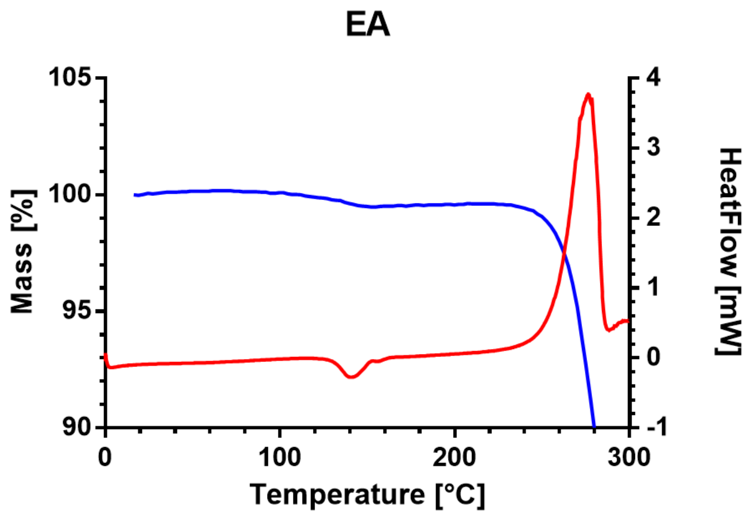

Fig. S10 TGA/DSC analysis of VEN EA

Tab. S3 TGA DSC and 1H NMR analysis of VEN EA

Solvent	DSC	TGA			1H NMR
	peak temp. endothermic [°C]	temp. range [°C]	mass loss [%]	residual solvent mol. eq.	residual solvent mol. eq.
EA	140.6	100 – 156<br>156 – 235	2.4<br>1.7	0.2<br>0.2	0.5
