# Supplementary material for: Unravelling venetoclax solvate behaviour: insights from crystal structures and computational surface analysis
Source: IUCrJ. 2025 Aug 28;12(Pt 5):595–609. doi: 10.1107/S2052252525006785 (PMC12403167; doi:10.1107/S2052252525006785)

checkCIF/PLATON report

Structure factors have been supplied for datablock(s) I

THIS REPORT IS FOR GUIDANCE ONLY. IF USED AS PART OF A REVIEW PROCEDURE FOR PUBLICATION, IT SHOULD NOT REPLACE THE EXPERTISE OF AN EXPERIENCED CRYSTALLOGRAPHIC REFEREE.

No syntax errors found.   CIF dictionary   Interpreting this report

Datablock: I

Bond precision: C-C = 0.0022 A   Wavelength=1.54180

Cell:	a=12.4625(1)	b=13.1131(2)	c=15.3650(2)
	alpha=93.2334(11)	beta=96.3237(10)	gamma=113.9359(12)
Temperature:	95 K		

	Calculated	Reported
Volume	2266.90(5)	2266.90(5)
Space group	P -1	P -1
Hall group	-P 1	?
Moiety formula	C45 H50 Cl N7 O7 S, C2 H3 N	C45 H50 Cl N7 O7 S, C2 H3 N
Sum formula	C47 H53 Cl N8 O7 S	C47 H53 Cl1 N8 O7 S1
Mr	909.48	909.51
Dx,g cm-3	1.332	1.332
Z	2	2
Mu (mm-1)	1.675	1.675
F000	960.0	960.0
F000'	964.12	
h,k,lmax	15,16,19	15,16,18
Nref	9145	8998
Tmin,Tmax	0.792,0.892	0.400,0.890
Tmin'	0.727	

Correction method= # Reported T Limits: Tmin=0.400 Tmax=0.890
AbsCorr = MULTI-SCAN

Data completeness= 0.984   Theta(max)= 73.465

R(reflections)= 0.0397(8362)   wR2(reflections)= 0.1032(8998)

S = 1.000   Npar= 788

The following ALERTS were generated. Each ALERT has the format
test-name_ALERT_alert-type_alert-level.
Click on the hyperlinks for more details of the test.

Alert level B

```
PLAT430_ALERT_2_B Short Inter D...A Contact  O44      ..O44      .       2.79 Ang.
                                                 -x,1-y,1-z  =      2_566 Check
```

Author Response: This interaction is based on the nature of non-bonding interactions between neighbouring molecules.

Alert level C

```
PLAT041_ALERT_1_C Calc. and Reported SumFormula    Strings  Differ     Please Check
              Calc: C47 H53 Cl N8 O7 S
              Rep.: C47 H53 Cl1 N8 O7 S1
PLAT213_ALERT_2_C Atom O61             has ADP max/min Ratio .....        3.4 prolat
PLAT213_ALERT_2_C Atom C9              has ADP max/min Ratio .....        3.5 prolat
PLAT244_ALERT_4_C Low    'Solvent' Ueq as Compared to Neighbors of        C85 Check
PLAT245_ALERT_2_C U(iso) H51       Smaller than U(eq) C5        by      0.027 Ang**2
PLAT245_ALERT_2_C U(iso) H71       Smaller than U(eq) C7        by      0.026 Ang**2
PLAT245_ALERT_2_C U(iso) H821      Smaller than U(eq) C82       by      0.017 Ang**2
PLAT250_ALERT_2_C Large U3/U1 Ratio for <U(i,j)> Tensor(Resd    1)        2.3 Note
PLAT334_ALERT_2_C Small <C-C> Benzene Dist.   C4       -C83      .       1.37 Ang.
PLAT430_ALERT_2_C Short Inter D...A Contact  O28      ..O44      .       2.89 Ang.
                                                 -x,1-y,1-z  =      2_566 Check
```

Author Response: This interaction is based on the nature of non-bonding interactions between neighbouring molecules.

```
PLAT737_ALERT_1_C D...A   Calc    3.031(2), Rep    3.031(6) ......        3.0 s.u.-R
              C37      -O54             1_555   1_445 ........     #    1 Check
PLAT790_ALERT_4_C Centre of Gravity not Within Unit Cell: Resd.  #          1 Note
              C45 H50 Cl N7 O7 S
PLAT911_ALERT_3_C Missing FCF Refl Between Thmin & STh/L=    0.600          2 Report
               -5 -9 12,   2-11 13,
```

Alert level G

```
PLAT002_ALERT_2_G Number of Distance or Angle Restraints on AtSite         54 Note
PLAT003_ALERT_2_G Number of Uiso or Uij Restrained non-H Atoms ...         10 Report
PLAT177_ALERT_4_G The CIF-Embedded .res File Contains DELU Records          3 Report
PLAT178_ALERT_4_G The CIF-Embedded .res File Contains SIMU Records          3 Report
PLAT188_ALERT_3_G A Non-default SIMU Restraint Value has been used     0.0051 Report
PLAT188_ALERT_3_G A Non-default SIMU Restraint Value has been used     0.0051 Report
PLAT188_ALERT_3_G A Non-default SIMU Restraint Value has been used     0.0051 Report
PLAT192_ALERT_3_G A Non-default DELU Restraint Value for First Par     0.0051 Report
PLAT192_ALERT_3_G A Non-default DELU Restraint Value for First Par     0.0051 Report
PLAT192_ALERT_3_G A Non-default DELU Restraint Value for First Par     0.0051 Report
PLAT230_ALERT_2_G Hirshfeld Test Diff for    Cl1      --C3       .        5.5 s.u.
```

```
PLAT230_ALERT_2_G Hirshfeld Test Diff for    C9       --C80      .        6.0 s.u.
PLAT301_ALERT_3_G Main Residue  Disorder ..............(Resd    1)        36% Note
PLAT410_ALERT_2_G Short Intra H...H Contact  H212     ..H261     .       1.90 Ang.
                                                  x,y,z  =     1_555 Check
PLAT410_ALERT_2_G Short Intra H...H Contact  H261     ..H222     .       1.94 Ang.
                                                  x,y,z  =     1_555 Check
PLAT410_ALERT_2_G Short Intra H...H Contact  H631     ..H641     .       2.06 Ang.
                                                  x,y,z  =     1_555 Check
PLAT410_ALERT_2_G Short Intra H...H Contact  H631     ..H651     .       1.79 Ang.
                                                  x,y,z  =     1_555 Check
PLAT432_ALERT_2_G Short Inter X...Y Contact  O43      ..C67      .       3.00 Ang.
                                              -x,-y,1-z  =     2_556 Check
PLAT769_ALERT_4_G CIF Embedded Explicitly Supplied Scattering Data     Please Note
PLAT790_ALERT_4_G Centre of Gravity not Within Unit Cell: Resd.  #          2 Note
              C2 H3 N
PLAT811_ALERT_5_G No ADDSYM Analysis: Too Many Excluded Atoms ....          ! Info
PLAT860_ALERT_3_G Number of Least-Squares Restraints ............         374 Note
PLAT882_ALERT_1_G No Datum for _diffrn_reflns_av_unetI/netI ......     Please Do !
PLAT912_ALERT_4_G Missing # of FCF Reflections Above STh/L=  0.600        137 Note
PLAT960_ALERT_3_G Number of Intensities with I  <   - 2*sig(I) ...          1 Check
PLAT969_ALERT_5_G The 'Henn et al.' R-Factor-gap value ..........       5.31 Note
              Predicted wR2: Based on SigI**2  1.94 or SHELX Weight 10.80
```

---

0 **ALERT level A** = Most likely a serious problem - resolve or explain
1 **ALERT level B** = A potentially serious problem, consider carefully
13 **ALERT level C** = Check. Ensure it is not caused by an omission or oversight
26 **ALERT level G** = General information/check it is not something unexpected

3 ALERT type 1 CIF construction/syntax error, inconsistent or missing data
18 ALERT type 2 Indicator that the structure model may be wrong or deficient
10 ALERT type 3 Indicator that the structure quality may be low
7 ALERT type 4 Improvement, methodology, query or suggestion
2 ALERT type 5 Informative message, check

It is advisable to attempt to resolve as many as possible of the alerts in all categories. Often the minor alerts point to easily fixed oversights, errors and omissions in your CIF or refinement strategy, so attention to these fine details can be worthwhile. In order to resolve some of the more serious problems it may be necessary to carry out additional measurements or structure refinements. However, the purpose of your study may justify the reported deviations and the more serious of these should normally be commented upon in the discussion or experimental section of a paper or in the "special_details" fields of the CIF. checkCIF was carefully designed to identify outliers and unusual parameters, but every test has its limitations and alerts that are not important in a particular case may appear. Conversely, the absence of alerts does not guarantee there are no aspects of the results needing attention. It is up to the individual to critically assess their own results and, if necessary, seek expert advice.

Publication of your CIF in IUCr journals

A basic structural check has been run on your CIF. These basic checks will be run on all CIFs submitted for publication in IUCr journals (*Acta Crystallographica*, *Journal of Applied Crystallography*, *Journal of Synchrotron Radiation*); however, if you intend to submit to *Acta Crystallographica Section C* or *E* or *IUCrData*, you should make sure that full publication checks are run on the final version of your CIF prior to submission.

Publication of your CIF in other journals

Please refer to the *Notes for Authors* of the relevant journal for any special instructions relating to CIF submission.

PLATON version of 06/01/2024; check.def file version of 05/01/2024

Datablock I - ellipsoid plot

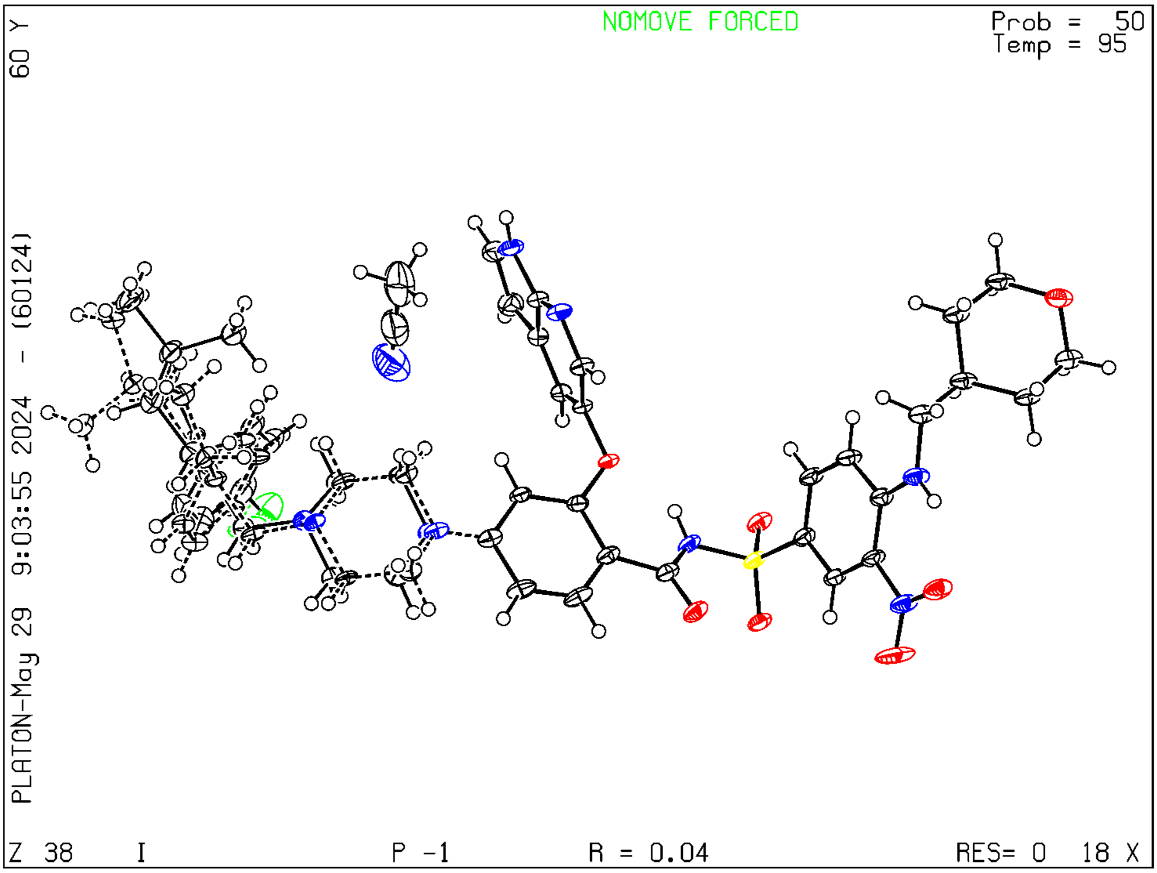

Supplement: Supplementary file 1 [file ACNp1_2.pdf › str for CCDC/ven acn2 final/ACNp1_2.pdf]
