# Supplementary material for: Unravelling venetoclax solvate behaviour: insights from crystal structures and computational surface analysis
Source: IUCrJ. 2025 Aug 28;12(Pt 5):595–609. doi: 10.1107/S2052252525006785 (PMC12403167; doi:10.1107/S2052252525006785)

checkCIF/PLATON report

Structure factors have been supplied for datablock(s) I

THIS REPORT IS FOR GUIDANCE ONLY. IF USED AS PART OF A REVIEW PROCEDURE FOR PUBLICATION, IT SHOULD NOT REPLACE THE EXPERTISE OF AN EXPERIENCED CRYSTALLOGRAPHIC REFEREE.

No syntax errors found. CIF dictionary Interpreting this report

Datablock: I

Bond precision:	C-C = 0.0047 A		Wavelength=1.54184
Cell:	a=12.0250(5)	b=13.6215(6)	c=15.7475(8)
	alpha=93.425(4)	beta=110.937(5)	gamma=102.318(4)
Temperature:	95 K		

	Calculated	Reported
Volume	2327.4(2)	2327.4(2)
Space group	P -1	P -1
Hall group	-P 1	?
Moiety formula	C45 H50 Cl N7 O7 S, C3 H6 O	C48 H56 Cl1 N7 O8 S1
Sum formula	C48 H56 Cl N7 O8 S	C48 H56 Cl1 N7 O8 S1
Mr	926.51	926.53
Dx,g cm-3	1.322	1.322
Z	2	2
Mu (mm-1)	1.650	1.651
F000	980.0	980.0
F000’	984.20	
h,k,lmax	14,16,19	14,16,19
Nref	9358	8525
Tmin,Tmax	0.860,0.961	0.650,0.960
Tmin’	0.808	

Correction method= # Reported T Limits: Tmin=0.650 Tmax=0.960
AbsCorr = MULTI-SCAN

Data completeness= 0.911 Theta(max)= 73.568

R(reflections)= 0.0560(5262) wR2(reflections)= 0.1483(8523)

S = 0.889 Npar= 586

The following ALERTS were generated. Each ALERT has the format
test-name_ALERT_alert-type_alert-level.
Click on the hyperlinks for more details of the test.

Alert level A

```
PLAT029_ALERT_3_A _diffrn_measured_fraction_theta_full value Low .      0.925 Why?
```

Author Response: Low intensity at high angles and twinned sample.

Alert level B

```
RINTA01_ALERT_3_B  The value of Rint is greater than 0.18
            Rint given   0.198
```

Author Response: Sample twinned. Rint from all data 0.0861. Twinning has to be considered. Without it, model could not be reasobanbly refined.

```
PLAT911_ALERT_3_B Missing FCF Refl Between Thmin & STh/L=    0.600        606 Report
                 1  1  0,    2  1  0,    4  1  0,    1  2  0,    2  2  0,    3  2  0,
                 4  2  0,    1  3  0,    2  3  0,    3  3  0,    4  3  0,    5  3  0,
                 6  3  0,    2  4  0,    3  4  0,    4  4  0,    5  4  0,    1  5  0,
                 2  5  0,    3  5  0,    4  5  0,    5  5  0,    6  5  0,   11  5  0,
                 0  6  0,    1  6  0,    2  6  0,    3  6  0,    4  6  0,    5  6  0,
                 6  6  0,    1  7  0,    2  7  0,    3  7  0,    4  7  0,    5  7  0,
                 6  7  0,    8  7  0,   10  7  0,    1  8  0,    2  8  0,    3  8  0,
                 4  8  0,    5  8  0,    6  8  0,    8  8  0,    9  8  0,    2  9  0,
                 3  9  0,    4  9  0,    5  9  0,    6  9  0,    7  9  0,    8  9  0,
                 9  9  0,    3 10  0,    4 10  0,    5 10  0,    7 10  0,    8 10  0,
                 1 11  0,    3 11  0,    4 11  0,    5 11  0,    6 11  0,    7 11  0,
                 3 12  0,    4 12  0,    5 12  0,    3 13  0,    4 13  0,    5 13  0,
                -5-13  1,   -4-13  1,   -6-12  1,   -5-12  1,   -4-12  1,   -7-11  1,
                -6-11  1,   -5-11  1,   -4-11  1,   -3-11  1,   -2-11  1,   -8-10  1,
                -7-10  1,   -5-10  1,   -4-10  1,   -3-10  1,   -2-10  1,   -1-10  1,
                -9 -9  1,   -8 -9  1,   -6 -9  1,   -5 -9  1,   -4 -9  1,   -3 -9  1,
```

Author Response: Twin1 component of the data used for refinement.

Alert level C

```
PLAT041_ALERT_1_C Calc. and Reported SumFormula    Strings  Differ     Please Check
              Calc: C48 H56 Cl N7 O8 S
              Rep.: C48 H56 Cl1 N7 O8 S1
PLAT042_ALERT_1_C Calc. and Reported MoietyFormula Strings  Differ     Please Check
              Calc: C45 H50 Cl N7 O7 S, C3 H6 O
              Rep.: C48 H56 Cl1 N7 O8 S1
PLAT340_ALERT_3_C Low Bond Precision on  C-C Bonds ..............    0.00475 Ang.
PLAT906_ALERT_3_C Large K Value in the Analysis of Variance ......     5.206 Check
PLAT934_ALERT_3_C Number of (Iobs-Icalc)/Sigma(W) > 10 Outliers ..         1 Check
```

```
          -10 -4 13,
```

Alert level G

```
PLAT007_ALERT_5_G Number of Unrefined Donor-H Atoms .............          3 Report
              H131  H141  H171
PLAT020_ALERT_3_G The Value of Rint is Greater Than 0.12 .........      0.198 Report
PLAT380_ALERT_4_G Incorrectly? Oriented X(sp2)-Methyl Moiety .....        C64 Check
PLAT769_ALERT_4_G CIF Embedded Explicitly Supplied Scattering Data     Please Note
PLAT790_ALERT_4_G Centre of Gravity not Within Unit Cell: Resd.  #          2 Note
              C3 H6 O
PLAT912_ALERT_4_G Missing # of FCF Reflections Above STh/L=  0.600        229 Note
PLAT960_ALERT_3_G Number of Intensities with I  <   - 2*sig(I) ...          3 Check
PLAT969_ALERT_5_G The 'Henn et al.' R-Factor-gap value ..........      4.611 Note
              Predicted wR2: Based on SigI**2  3.22 or SHELX Weight 16.67
```

---

1 **ALERT level A** = Most likely a serious problem - resolve or explain
2 **ALERT level B** = A potentially serious problem, consider carefully
5 **ALERT level C** = Check. Ensure it is not caused by an omission or oversight
8 **ALERT level G** = General information/check it is not something unexpected

2 ALERT type 1 CIF construction/syntax error, inconsistent or missing data
0 ALERT type 2 Indicator that the structure model may be wrong or deficient
8 ALERT type 3 Indicator that the structure quality may be low
4 ALERT type 4 Improvement, methodology, query or suggestion
2 ALERT type 5 Informative message, check

It is advisable to attempt to resolve as many as possible of the alerts in all categories. Often the minor alerts point to easily fixed oversights, errors and omissions in your CIF or refinement strategy, so attention to these fine details can be worthwhile. In order to resolve some of the more serious problems it may be necessary to carry out additional measurements or structure refinements. However, the purpose of your study may justify the reported deviations and the more serious of these should normally be commented upon in the discussion or experimental section of a paper or in the "special_details" fields of the CIF. checkCIF was carefully designed to identify outliers and unusual parameters, but every test has its limitations and alerts that are not important in a particular case may appear. Conversely, the absence of alerts does not guarantee there are no aspects of the results needing attention. It is up to the individual to critically assess their own results and, if necessary, seek expert advice.

Publication of your CIF in IUCr journals

A basic structural check has been run on your CIF. These basic checks will be run on all CIFs submitted for publication in IUCr journals (*Acta Crystallographica*, *Journal of Applied Crystallography*, *Journal of Synchrotron Radiation*); however, if you intend to submit to *Acta Crystallographica Section C* or *E* or *IUCrData*, you should make sure that full publication checks are run on the final version of your CIF prior to submission.

Publication of your CIF in other journals

Please refer to the *Notes for Authors* of the relevant journal for any special instructions relating to CIF submission.

PLATON version of 13/05/2024; check.def file version of 04/05/2024

Datablock I - ellipsoid plot

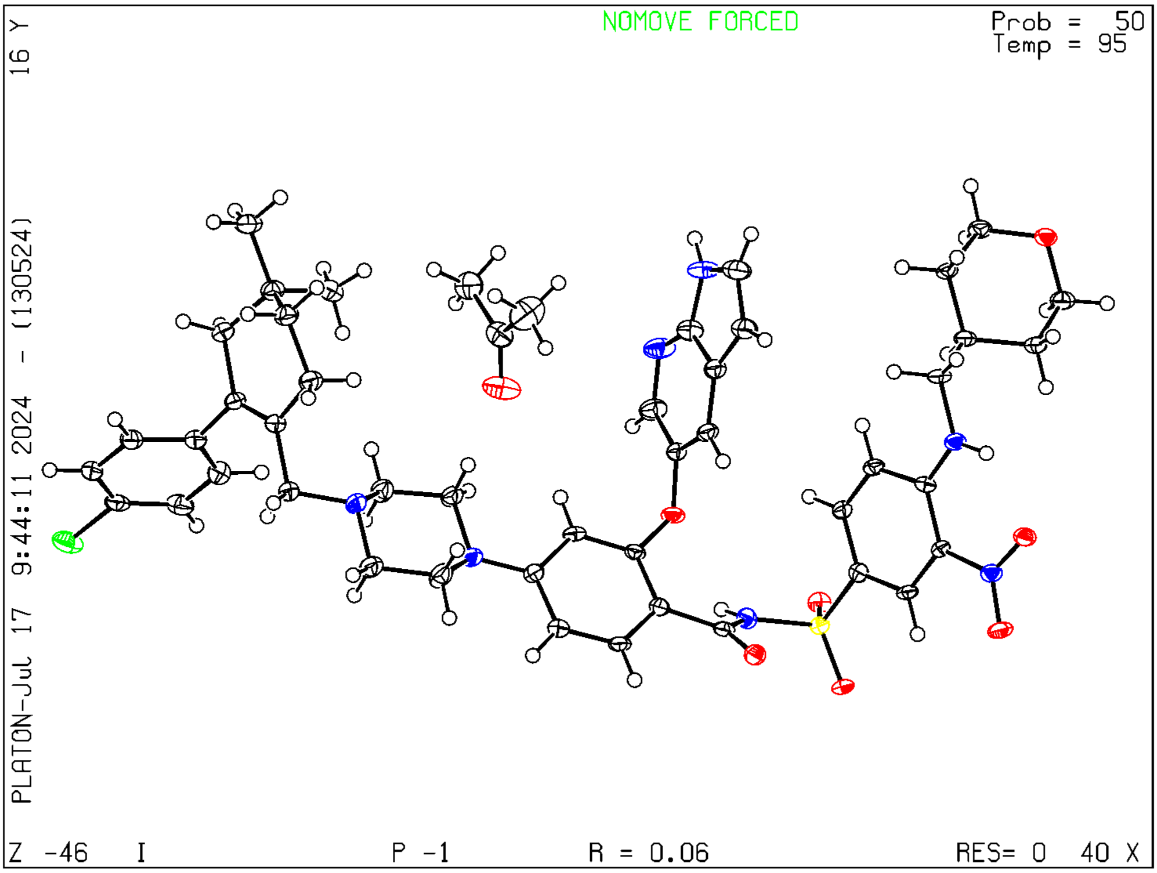

Supplement: Supplementary file 1 [file checkcif (1).pdf › str for CCDC/ven AE final/checkcif (1).pdf]
