# Supplementary material for: Unravelling venetoclax solvate behaviour: insights from crystal structures and computational surface analysis
Source: IUCrJ. 2025 Aug 28;12(Pt 5):595–609. doi: 10.1107/S2052252525006785 (PMC12403167; doi:10.1107/S2052252525006785)

checkCIF/PLATON report

Structure factors have been supplied for datablock(s) I

THIS REPORT IS FOR GUIDANCE ONLY. IF USED AS PART OF A REVIEW PROCEDURE FOR PUBLICATION, IT SHOULD NOT REPLACE THE EXPERTISE OF AN EXPERIENCED CRYSTALLOGRAPHIC REFEREE.

No syntax errors found. CIF dictionary Interpreting this report

Datablock: I

Bond precision:	C-C = 0.0046 A		Wavelength=1.54180
Cell:	a=14.6928(1)	b=12.3178(1)	c=29.8810(2)
	alpha=90	beta=93.2176(6)	gamma=90
Temperature:	120 K		

	Calculated	Reported
Volume	5399.43(7)	5399.43(7)
Space group	P 21/n	P 21/n
Hall group	-P 2yn	?
Moiety formula	C45 H50 Cl N7 O7 S, C5 H9 O, C5 H10 O	C55 H69 Cl1 N7 O9 S1
Sum formula	C55 H69 Cl N7 O9 S	C55 H69 Cl1 N7 O9 S1
Mr	1039.68	1039.71
Dx,g cm-3	1.279	1.279
Z	4	4
Mu (mm-1)	1.493	1.493
F000	2212.0	2212.0
F000'	2221.07	
h,k,lmax	17,14,35	17,14,35
Nref	9780	9722
Tmin,Tmax	0.746,0.821	0.790,0.820
Tmin'	0.644	

Correction method= # Reported T Limits: Tmin=0.790 Tmax=0.820
AbsCorr = MULTI-SCAN

Data completeness= 0.994 Theta(max)= 67.748

R(reflections)= 0.0667(8904) wR2(reflections)= 0.1973(9722)

S = 0.977 Npar= 677

```
The following ALERTS were generated. Each ALERT has the format
       test-name_ALERT_alert-type_alert-level.
Click on the hyperlinks for more details of the test.
```

Alert level B

```
PLAT250_ALERT_2_B Large U3/U1 Ratio for <U(i,j)> Tensor(Resd    2)        7.5 Note
```

Author Response: There is a disorder in one of the solvent sites. Two main positions were succesfully modeled, but it is likely that more overlapped positions are present, with a very low occupancy. These, it was impossible to model. To get a stable refinement, the ADPs of this molecule were restrained to be similar. This causes this alert.

Alert level C

```
DIFMX02_ALERT_1_C  The maximum difference density is > 0.1*ZMAX*0.75
            The relevant atom site should be identified.
PLAT041_ALERT_1_C Calc. and Reported SumFormula    Strings  Differ     Please Check
              Calc: C55 H69 Cl N7 O9 S
              Rep.: C55 H69 Cl1 N7 O9 S1
PLAT042_ALERT_1_C Calc. and Reported MoietyFormula Strings  Differ     Please Check
              Calc: C45 H50 Cl N7 O7 S, C5 H9 O, C5 H10 O
              Rep.: C55 H69 Cl1 N7 O9 S1
PLAT097_ALERT_2_C Large Reported Max.  (Positive) Residual Density       1.31 eA-3
PLAT234_ALERT_4_C Large Hirshfeld Difference O68      --C73      .       0.23 Ang.
PLAT234_ALERT_4_C Large Hirshfeld Difference C1       --C70      .       0.18 Ang.
PLAT234_ALERT_4_C Large Hirshfeld Difference C70      --C69      .       0.18 Ang.
PLAT234_ALERT_4_C Large Hirshfeld Difference C72      --C73      .       0.16 Ang.
PLAT243_ALERT_4_C High   'Solvent' Ueq as Compared to Neighbors of        O68 Check
PLAT244_ALERT_4_C Low    'Solvent' Ueq as Compared to Neighbors of        C70 Check
PLAT244_ALERT_4_C Low    'Solvent' Ueq as Compared to Neighbors of        C73 Check
PLAT250_ALERT_2_C Large U3/U1 Ratio for <U(i,j)> Tensor(Resd    3)        2.8 Note
```

Author Response: There is a disorder in one of the solvent sites. Two main positions were succesfully modeled, but it is likely that more overlapped positions are present, with a very low occupancy. These, it was impossible to model. To get a stable refinement, the ADPs of this molecule were restrained to be similar. This causes this alert.

```
PLAT260_ALERT_2_C Large Average Ueq of Residue Including       O68      0.251 Check
PLAT260_ALERT_2_C Large Average Ueq of Residue Including       O62      0.105 Check
PLAT340_ALERT_3_C Low Bond Precision on  C-C Bonds ..............    0.00464 Ang.
PLAT362_ALERT_2_C Short  C(sp3)-C(sp2) Bond  C72       - C73      .       1.40 Ang.
PLAT410_ALERT_2_C Short Intra H...H Contact  H471      ..H582     .       1.94 Ang.
                                                       x,y,z  =      1_555 Check
PLAT906_ALERT_3_C Large K Value in the Analysis of Variance ......      2.601 Check
PLAT911_ALERT_3_C Missing FCF Refl Between Thmin & STh/L=    0.600          41 Report
               16  6  0,  16  6  1,  16  6  2,   8 13  4, -10 12  5,  -8 13  5,
              -10 12  6,  -8 13  6,  15  7  6,  14  8  8, -11 11  9,   9 12  9,
```

```
               -7 13 10,  -3 14 10,  -9 12 11,   1 14 11,  10 11 11,  16  3 11,
                8 12 12,  14  7 12,  16  0 12,  16  1 12,  16  1 13,  15  0 17,
              -12  8 19,  -9 10 20,  -7 11 20,   5 11 21,  11  7 21,  -5 11 22,
                4 11 22, -10  8 23,  -7 10 23,  12  4 23,  -6 10 24,  -3  9 28,
               -2  8 30,   4  7 30,   4  4 33,   0  3 35,   2  2 35,
PLAT918_ALERT_3_C Reflection(s) with I(obs) much Smaller I(calc) .          1 Check
PLAT934_ALERT_3_C Number of (Iobs-Icalc)/Sigma(W) > 10 Outliers ..          1 Check
               -3  2  1,
```

Alert level G

```
PLAT002_ALERT_2_G Number of Distance or Angle Restraints on AtSite         14 Note
PLAT003_ALERT_2_G Number of Uiso or Uij Restrained non-H Atoms ...          4 Report
PLAT007_ALERT_5_G Number of Unrefined Donor-H Atoms .............           3 Report
              H251  H361  H531
PLAT072_ALERT_2_G SHELXL First  Parameter in WGHT  Unusually Large       0.12 Report
PLAT083_ALERT_2_G SHELXL Second Parameter in WGHT  Unusually Large       9.05 Why ?
PLAT142_ALERT_4_G s.u. on b - Axis Small or Missing .............     0.00010 Ang.
PLAT143_ALERT_4_G s.u. on c - Axis Small or Missing .............     0.00020 Ang.
PLAT175_ALERT_4_G The CIF-Embedded .res File Contains SAME Records          2 Report
PLAT177_ALERT_4_G The CIF-Embedded .res File Contains DELU Records          1 Report
PLAT178_ALERT_4_G The CIF-Embedded .res File Contains SIMU Records          1 Report
PLAT188_ALERT_3_G A Non-default SIMU Restraint Value has been used     0.0100 Report
PLAT231_ALERT_4_G Hirshfeld Test (Solvent)   O68      --C69      .        7.1 s.u.
PLAT302_ALERT_4_G Anion/Solvent/Minor-Residue Disorder (Resd    2)        33% Note
PLAT410_ALERT_2_G Short Intra H...H Contact  H721     ..H711     .       2.13 Ang.
                                                   x,y,z  =      1_555 Check
PLAT413_ALERT_2_G Short Inter XH3 .. XHn     H122     ..H151     .       1.85 Ang.
                                              2-x,-y,1-z  =      2_756 Check
PLAT413_ALERT_2_G Short Inter XH3 .. XHn     H133     ..H152     .       1.73 Ang.
                                              2-x,-y,1-z  =      2_756 Check
PLAT413_ALERT_2_G Short Inter XH3 .. XHn     H133     ..H712     .       1.55 Ang.
                                              2-x,-y,1-z  =      2_756 Check
PLAT769_ALERT_4_G CIF Embedded Explicitly Supplied Scattering Data     Please Note
PLAT860_ALERT_3_G Number of Least-Squares Restraints ............         148 Note
PLAT909_ALERT_3_G Percentage of I>2sig(I) Data at Theta(Max) Still        83% Note
PLAT912_ALERT_4_G Missing # of FCF Reflections Above STh/L=  0.600         17 Note
PLAT960_ALERT_3_G Number of Intensities with I  <   - 2*sig(I) ...          1 Check
PLAT969_ALERT_5_G The 'Henn et al.' R-Factor-gap value ..........       12.31 Note
              Predicted wR2: Based on SigI**2  1.60 or SHELX Weight 20.93
```

---

 0 **ALERT level A** = Most likely a serious problem - resolve or explain
 1 **ALERT level B** = A potentially serious problem, consider carefully
21 **ALERT level C** = Check. Ensure it is not caused by an omission or oversight
23 **ALERT level G** = General information/check it is not something unexpected

 3 ALERT type 1 CIF construction/syntax error, inconsistent or missing data
15 ALERT type 2 Indicator that the structure model may be wrong or deficient
 9 ALERT type 3 Indicator that the structure quality may be low
16 ALERT type 4 Improvement, methodology, query or suggestion
 2 ALERT type 5 Informative message, check

It is advisable to attempt to resolve as many as possible of the alerts in all categories. Often the minor alerts point to easily fixed oversights, errors and omissions in your CIF or refinement strategy, so attention to these fine details can be worthwhile. In order to resolve some of the more serious problems it may be necessary to carry out additional measurements or structure refinements. However, the purpose of your study may justify the reported deviations and the more serious of these should normally be commented upon in the discussion or experimental section of a paper or in the "special_details" fields of the CIF. checkCIF was carefully designed to identify outliers and unusual parameters, but every test has its limitations and alerts that are not important in a particular case may appear. Conversely, the absence of alerts does not guarantee there are no aspects of the results needing attention. It is up to the individual to critically assess their own results and, if necessary, seek expert advice.

Publication of your CIF in IUCr journals

A basic structural check has been run on your CIF. These basic checks will be run on all CIFs submitted for publication in IUCr journals (*Acta Crystallographica*, *Journal of Applied Crystallography*, *Journal of Synchrotron Radiation*); however, if you intend to submit to *Acta Crystallographica Section C* or *E* or *IUCrData*, you should make sure that full publication checks are run on the final version of your CIF prior to submission.

Publication of your CIF in other journals

Please refer to the *Notes for Authors* of the relevant journal for any special instructions relating to CIF submission.

PLATON version of 06/01/2024; check.def file version of 05/01/2024

Datablock I - ellipsoid plot

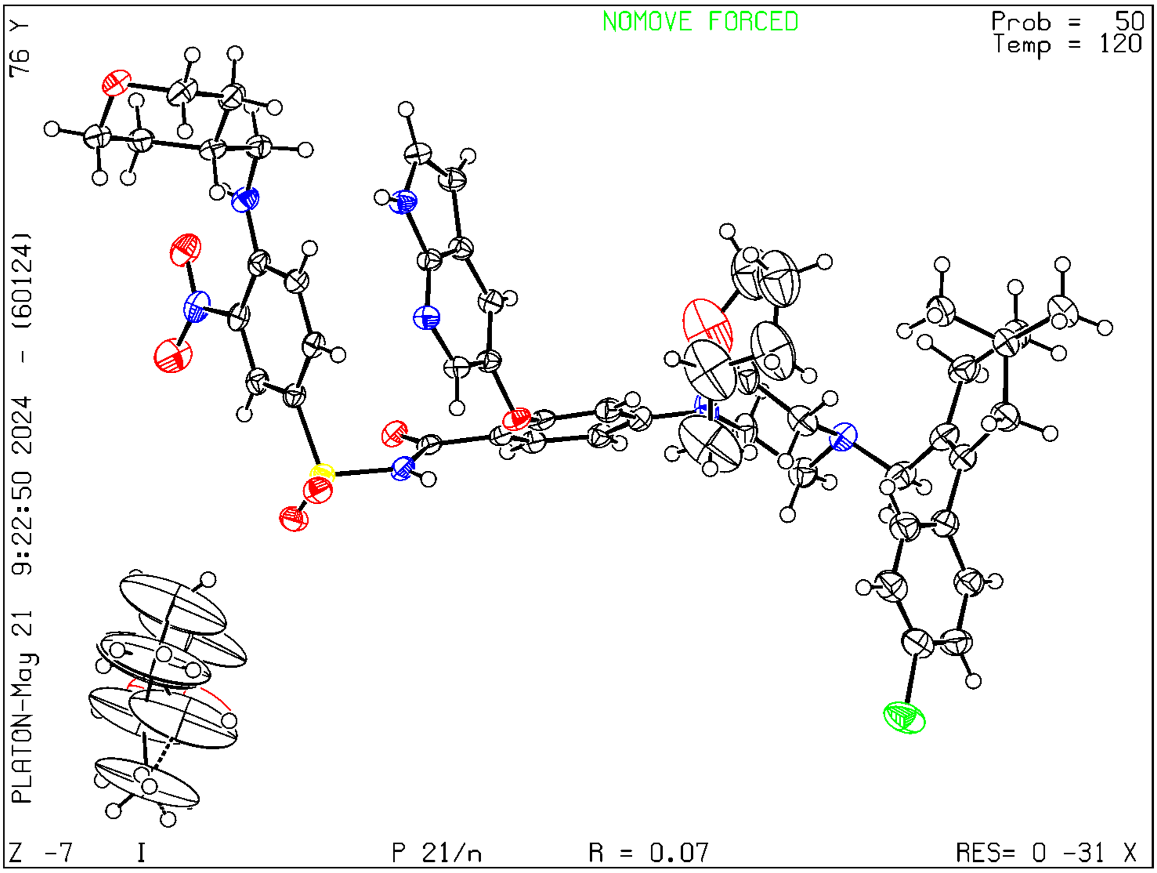

Supplement: Supplementary file 1 [file checkcif (1).pdf › str for CCDC/ven MHF final/checkcif (1).pdf]
